# Tumor Small Extracellular Vesicle‐Transmitted LncRNA CATED Promotes Platinum‐Resistance in High‐Grade Serous Ovarian Cancer

**DOI:** 10.1002/advs.202505963

**Published:** 2025-06-10

**Authors:** Yi Liu, Hanyuan Liu, Chenchen Zhu, Yan Yang, Zhen Shen, Ge Shan, Liang Chen, Ying Zhou

**Affiliations:** ^1^ Department of Obstetrics and Gynecology Core Facility Center The First Affiliated Hospital of USTC Division of Life Sciences and Medicine University of Science and Technology of China Hefei Anhui 230001 China; ^2^ Hefei National Laboratory for Physical Sciences at Microscale School of Basic Medical Sciences Division of Life Sciences and Medicine University of Science and Technology of China Hefei 230027 China; ^3^ Department of Obstetrics and Gynecology The First Affiliated Hospital of USTC The RNA Institute School of Basic Medical Sciences Division of Life Sciences and Medicine University of Science and Technology of China Hefei 230027 China; ^4^ Department of Cardiology, The First Affiliated Hospital of USTC The RNA Institute Division of Life Sciences and Medicine University of Science and Technology of China Hefei 230027 China

**Keywords:** DHX36, lncRNAs, ovarian cancer, platinum resistance, small extracellular vesicles

## Abstract

High‐grade serous ovarian cancer (HGSOC) is the most lethal type of gynecological cancer, and platinum‐resistance is a serious challenge in its treatment. Long non‐coding RNAs (lncRNAs) play critical regulatory roles in the occurrence and development of cancers. Here, using RNA sequencing of tumor small extracellular vesicles (sEVs) from HGSOC patients, the lncRNA CATED is identified as significantly upregulated in both tumors and tumor‐derived sEVs in platinum‐resistant HGSOC, and low CATED levels correlate with good prognosis. Functionally, CATED enhances cisplatin resistance by promoting cell proliferation and inhibiting apoptosis in vitro and in vivo. These effects could be transferred via CATED‐overexpressing sEVs from donor cells and HGSOC tumor sEVs. Mechanistically, CATED binds to and upregulates DHX36 via PIAS1‐mediated SUMOylation at the K105 site, and elevated DHX36 levels increase downstream RAP1A protein levels by enhancing RAP1A mRNA translation, consequently activating the MAPK pathway to promote platinum‐resistance in HGSOC. Antisense oligonucleotide mediated knockdown of CATED reverse platinum‐resistance in sEV‐transmitted mouse models via the DHX36‐RAP1A‐MAPK pathway. This study newly identifies a sEV‐transmitted lncRNA CATED in driving HGSOC platinum‐resistance and elucidates the mechanism it regulates the interacting protein through SUMOylation. These findings also provide a novel strategy for improving chemotherapy in HGSOC by targeting CATED.

## Graphical abstract

Overcoming platinum resistance in high‐grade serous ovarian cancer (HGSOC) is critical for improving outcomes. This study identifies lncRNA CATED, elevated in platinum‐resistant small extracellular vesicles (sEVs) and tumors, which drives cisplatin resistance by enhancing DHX36 SUMOylation, boosting RAP1A translation, and activating MAPK signaling. Targeting CATED with antisense oligonucleotides reverses resistance, offering a novel therapeutic strategy for HGSOC.

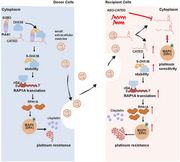

## 1 Introduction

Epithelial ovarian cancer (EOC) is the third most prevalent and fatal malignancy of the female reproductive system, with high‐grade serous ovarian cancer (HGSOC) being the predominant subtype.^[^ ^1^, ^2^ ^]^ The first‐line standard of care for HGSOC includes surgery followed by platinum‐based chemotherapy. However, ≈70% of patients with advanced HGSOC develop recurrence with platinum resistance following chemotherapeutic treatment and exhibit a poor prognosis.^[^ ^3^ ^]^ Chemoresistance poses a formidable challenge in HGSOC treatment. Platinum resistance in HGSOC remains multifactorial and is influenced by diverse environmental and genetic factors. The major mechanisms of platinum resistance in HGSOC include abnormal transmembrane transport, alterations in DNA damage repair, small extracellular vesicle (sEV)‐mediated intercellular communication and dysregulation of cancer‐associated signaling pathways such as the MAPK pathway.^[^ ^4^ ^]^ Exploring strategies and identifying critical targets to overcome platinum resistance in HGSOC represents a critical scientific endeavor to improve patient prognosis.

Growing evidence has highlighted the essential regulatory roles of long non‐coding RNAs (lncRNAs) in key cancer hallmarks such as metastasis, genomic instability, and drug resistance. For example, upregulated lncRNA HOTAIR in colorectal cancer is associated with live metastasis and reprogramming of PRC2 function;^[^ ^5^ ^]^ lncRNA MATAT1 regulates a set of metastasis‐associated genes, and antisense oligonucleotide (ASO) targeting MALAT1 prevents metastasis formation after tumor implantation, which practically proves the efficacy of targeting oncogenic lncRNAs in vivo using ASOs.^[^ ^6^ ^]^ Multiple lncRNAs have also been reported to play a role in drug resistance in cancers, as is exemplified by the lncRNA HIF1A‐AS1, which contributes to gemcitabine resistance in pancreatic cancer by upregulating glycolysis.^[^ ^7^ ^]^ A few studies have indicated the potential involvement of lncRNAs in HGSOC platinum resistance;^[^ ^8^, ^9^ ^]^ for example, lncRNA PLADE modulates R‐loop dynamics to enhance chemosensitivity.^[^ ^8^ ^]^ However, lncRNAs with strong functions in HGSOC chemoresistance and the underlying mechanisms have rarely been reported.

Small extracellular vesicles (sEVs), with diameters <200 nm, function as intermediaries in cell‐to‐cell communication and play a crucial role in tumor progression, invasion, metastasis, and resistance to chemotherapy.^[^ ^10^, ^11^, ^12^ ^]^ Several studies have demonstrated that sEVs deliver lncRNAs to regulate pathophysiological processes.^[^ ^13^ ^]^ For example, tumor‐associated macrophage derived sEV‐transmitted LINC01232 promotes glioma immune escape by regulating the E2F2/NBR1/MHC‐I axis, enhancing tumor growth.^[^ ^14^ ^]^ However, an in‐depth understanding of the functional roles of sEV‐transmitted lncRNAs in chemoresistance, their capability to trigger tumorigenesis of adjacent tissues, and the underlying mechanisms remain to be fully elucidated, warranting further exploration.

To explore the roles of sEV‐transmitted lncRNAs in HGSOC platinum‐resistance, we utilized RNA‐seq for differential expression of lncRNAs in HGSOC platinum‐resistant and platinum‐sensitive HGSOC patient's tumor‐derived sEVs and identified a novel lncRNA, CATED (**C**isplatin‐**A**ssociated **T**umor small **E**xtracellular Vesicle‐**D**erived LncRNA, ENSG00000267058) upregulated in tumor‐derived sEVs from patients with platinum‐resistant HGSOC. Through a combination of bioinformatics, molecular, biochemical, and cellular analysis, we provided evidence supporting the role of CATED in promoting HGSOC cisplatin resistance, either by direct cell overexpression or sEV‐mediated delivery. Our findings revealed that CATED could bind to and upregulate DHX36 by PIAS1‐mediated SUMOylation, thus promoting downstream RAP1A translation, consequently leading to activation of the MAPK pathway to increase cisplatin resistance. Targeting CATED with ASO effectively restores chemosensitivity, providing a potential therapeutic approach to prevent platinum resistance, as it serves as both a predictive marker and a therapeutic target.

## 2 Results

### 2.1 LncRNA CATED is Upregulated in the Tumor‐Derived sEVs of Platinum‐Resistant HGSOC Patients

**Figure 1:**
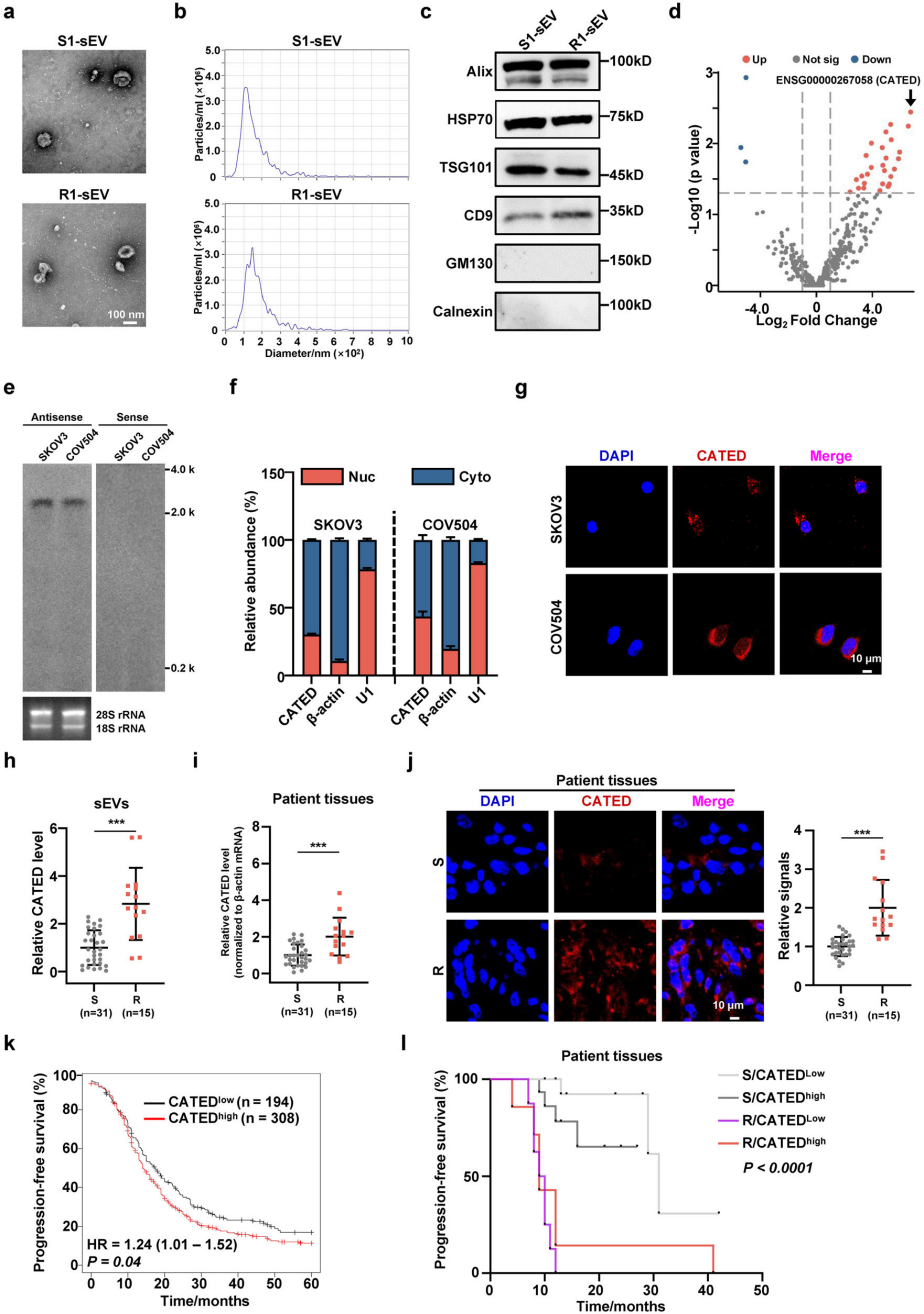
Identification and characterization of CATED in HGSOC. a) Representative images of isolated small extracellular vesicles (sEVs) from HGSOC tumor, captured using transmission electron microscopy (TEM), with a scale bar of 100 nm. S, sensitive; R, resistant; sEV, small extracellular vesicle. b) Size analysis of the isolated tumor‐derived sEVs using Nanoparticle Tracking Analysis (NTA). c) Western blotting results demonstrating the presence of sEV markers Alix, HSP70, TSG101, and CD9, with GM130 and Calnexin serving as negative controls. d) Volcano plots showing differential expression of lncRNAs in tumor‐derived sEVs between platinum‐sensitive (n = 4) and ‐resistant (n = 3) samples. Blue dots indicate significantly down‐regulated lncRNAs, while red dots represent significantly upregulated lncRNAs. e) Northern blot analysis of CATED expression in SKOV3 and COV504 cells. An antisense probe targeting CATED was used for detection, with a sense probe serving as the negative control. The 28S and 18S rRNA bands are shown as loading controls. f) RT‐qPCR analysis of CATED in the nuclear/cytoplasmic fraction of SKOV3 and COV504 cells. Nuc, nuclear; Cyto, cytoplasmic. g) Showcase FISH images of CATED (red) in SKOV3 and COV504 cells. DAPI staining of nuclei. Scale bar: 10 µm. h) Levels of CATED in tumor sEVs from platinum‐sensitive patients (n = 31) and platinum‐resistant patients (n = 15). i) Levels of CATED in HGSOC tumor from platinum‐sensitive patients (n = 31) and platinum‐resistant patients (n = 15). j) FISH images of CATED in HGSOC tumors from platinum‐sensitive patients (n = 31) and platinum‐resistant patients (n = 15) and quantification of relative levels of CATED FISH are shown. Scale bar: 10 µm. k,l) Kaplan–Meier analysis of progression‐free survival for Kaplan‐Meier Plotter Database (k) and 46 HGSOC patients (l). Data are shown as mean ± SD from at least three independent experiments. *p*‐values are from two‐sided unpaired Student's *t*‐test for h‐j. *P*‐values are calculated by the log‐rank test for k, l. ***p < 0.001.

To investigate the functional roles of lncRNAs in sEVs derived from the tumors of platinum‐resistant HGSOC patients, we first isolated and purified sEVs from the tumors of platinum‐resistant and platinum‐sensitive HGSOC patients (Figure S1a, Table S1, Supporting Information). The isolated sEVs were characterized and quantified using Transmission Electron Microscopy (TEM), Nanoparticle Tracking Analysis (NTA), and western blotting (**Figure** **1**a–c; Figure S1b–d, Supporting Information). Ribosomal RNA‐depleted RNA‐seq of seven tumor‐derived sEVs specimens was performed, of which three were from platinum‐resistant patients and the other four were from platinum‐sensitive patients. A total of 409 lncRNAs were identified as differentially expressed, and the lncRNA (ENSG00000267058), named CATED (Cisplatin‐**A**ssociated **T**umor small **E**xtracellular Vesicle‐**D**erived LncRNA CATED), was the most upregulated lncRNA in platinum‐resistant patients compared to platinum‐sensitive patients (Figure 1d). CATED is comprised of three exons, located on chromosome 19 (Figure S2a, Supporting Information), and is found to be expressed across multiple tumors as is analyzed in the TCGA database (Figure S2b, Supporting Information). The 5’ and 3’ Rapid‐amplification of cDNA ends (RACE) assays and northern blotting both confirmed the full length of the CATED transcript in SKOV3 and COV504 cell lines (Figure 1e; Figure S2c, Table S2, Supporting Information). CATED was predicted to lack the coding potential (Figure S2d, Supporting Information), and sucrose gradient fractionation polysome profiling revealed that CATED was not enriched in either the low molecular weight (LMW) fraction or high molecular weight (HMW) fraction (Figure S2e, Supporting Information). Three open reading frames (ORF) were predicted by ORFfinder, and the GFP sequence inserted before the stop codon of these ORFs produced undetected GFP products, further confirming its lack of coding potential (Figure S2f, Supporting Information).

Fluorescence in situ Hybridization (FISH) analysis of CATED and cell fractionation with subsequent subcellular RT‐qPCR analysis demonstrated that CATED was mainly localized in the cytoplasm (Figure 1f,g). We grouped 46 HGSOC patients as platinum‐resistant (n = 15) and platinum‐sensitive (n = 31) based on their clinical response to platinum treatment and collected their tumor specimens and tumor‐derived sEVs. We observed significantly higher CATED levels in both tumors and tumor‐derived sEVs from platinum‐resistant patients than in those from platinum‐sensitive patients (Figure 1h,i, Table S3, Supporting Information). Higher levels of CATED were also observed by FISH in platinum‐resistant HGSOC specimens than in the platinum‐sensitive group (Figure 1j). Progression‐free survival (PFS) analysis using the Kaplan‐Meier Plotter Database showed that a high CATED level predicted poor prognosis (Figure 1k). We further classified platinum‐resistant and platinum‐sensitive groups from our 46 HGSOC patient cohort into four subgroups depending on CATED levels, and Kaplan‐Meier analysis showed that higher CATED levels correlated with shorter PFS (Figure 1l).

Collectively, these results indicate that elevated CATED levels were closely associated with platinum resistance and poor prognosis in HGSOC, as corroborated by the data from clinical specimens.

### 2.2 CATED Promotes HGSOC Proliferation and Inhibits Apoptosis In Vitro and In Vivo

**Figure 2:**
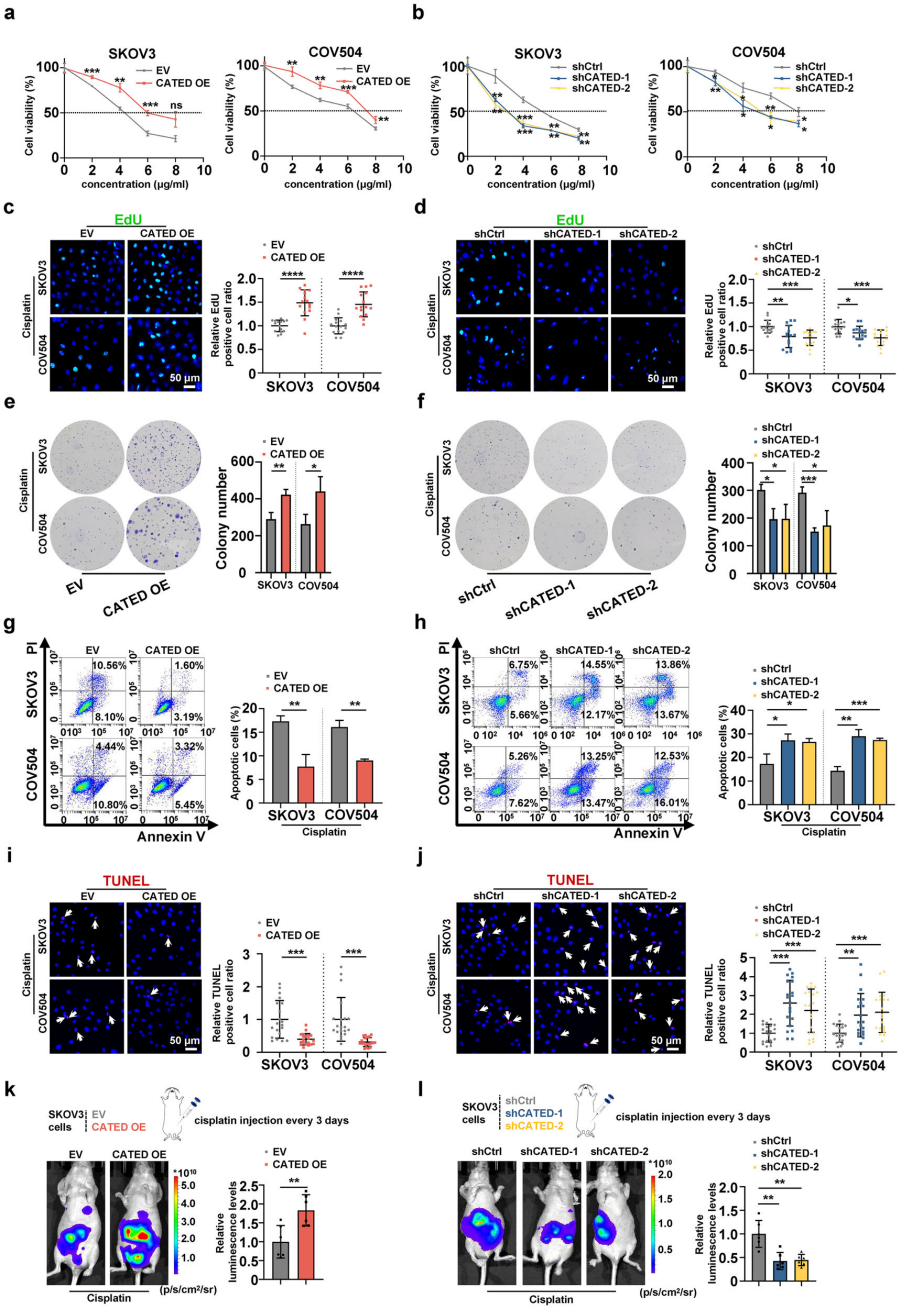
CATED promotes cell proliferation and inhibits apoptosis in HGSOC. a,b) Cell viability assessed by CCK8 assay following CATED overexpression or knockdown in SKOV3 and COV504 cells. c,d) EdU assays of SKOV3 and COV504 cells overexpressed or knocked down CATED. Scale bar: 50 µm. e,f) Colony formation assays for cells with CATED overexpression or knockdown. g,h) Apoptosis analysis was conducted using flow cytometry following CATED overexpression or knockdown in SKOV3 and COV504 cells. i,j) TUNEL staining of CATED overexpression or knockdown cells. k) Representative images (left) and quantification (right) of intraperitoneal tumor‐bearing nude mice injected with SKOV3 CATED OE cells with luciferase and treated with cisplatin at the specified time (n = 6 mice per group). l) Representative images (left) and quantification (right) of intraperitoneal tumor‐bearing nude mice injected with SKOV3 CATED knockdown cells with luciferase and treated with cisplatin at the specified time points (n = 6 mice per group). All assays above were conducted under conditions where cells were treated with cisplatin at the indicated concentrations for 36 h. Dated are shown as mean ± SD for the bar figure. *p* values were determined with two‐sided Student's *t*‐test. *p < 0.05, **p < 0.01, ***p < 0.001, ****p < 0.0001. All data were from at least three repeats.

To explore the function of CATED in HGSOC platinum resistance, we established stable cell lines with CATED overexpression and knockdown in SKOV3 and COV504 cells (Figure S3a, Supporting Information), which are two cell lines commonly used in ovarian cancer research.^[^ ^15^, ^16^ ^]^ Increased cell viability was observed in the CCK8 assay upon CATED overexpression, whereas CATED knockdown decreased cell viability (**Figure** **2**a,b). Upon treatment with cisplatin, EdU assays and colony formation showed that CATED overexpression significantly promoted cell proliferation, whereas CATED knockdown significantly suppressed these phenotypes (Figure 2c–f). Cisplatin generally exerts cytotoxic effects by triggering irreversible cell apoptosis.^[^ ^17^ ^]^ We investigated the association between CATED and apoptosis. Flow cytometry (FCM) and TUNEL assays showed that overexpression of CATED suppressed apoptosis in both cell lines, while CATED knockdown increased apoptosis (Figure 2g–j), with apoptotic markers, cleaved‐Caspase 3 and cleaved PARP confirmed the above results (Figure S3b,c, Supporting Information). These data from stable cell lines proved that CATED promoted cell proliferation and inhibited apoptosis.

We further confirmed the role of CATED in promoting cisplatin resistance in vivo using a mouse model. Stable CATED‐overexpressing SKOV3 cells or corresponding control cells were injected into nude mice intraperitoneally with cisplatin treatment at the indicated time points. We observed that CATED overexpression promoted tumor progression as measured by bioluminescence imaging (Figure 2k; Figure S3d, Supporting Information). Mouse tumors were collected and CATED levels were markedly elevated in the CATED‐overexpressing group (Figure S3e, Supporting Information). Immunohistochemical (IHC) of the tumors revealed that Ki‐67 levels were increased and cleaved‐Caspase 3 levels were decreased in the CATED overexpression group compared with those in the control group (Figure S3f,g, Supporting Information). Additionally, stable CATED‐knockdown SKOV3 cells or corresponding control cells were injected into nude mice intraperitoneally with cisplatin treatment at the indicated time points. Bioluminescence imaging revealed that CATED knockdown inhibited tumor progression (Figure 2l; Figure S3h, Supporting Information). The levels of CATED and Ki‐67 in tumor tissues decreased, with the levels of cleaved‐Caspase 3 increased in the CATED‐knockdown groups compared to the control group (Figure S3i–k, Supporting Information). These results confirmed that CATED exerted its pro‐tumor functions both in vitro and in vivo.

### 2.3 CATED Functions in Promoting Cisplatin‐Resistance can be Transferred by sEVs

**Figure 3:**
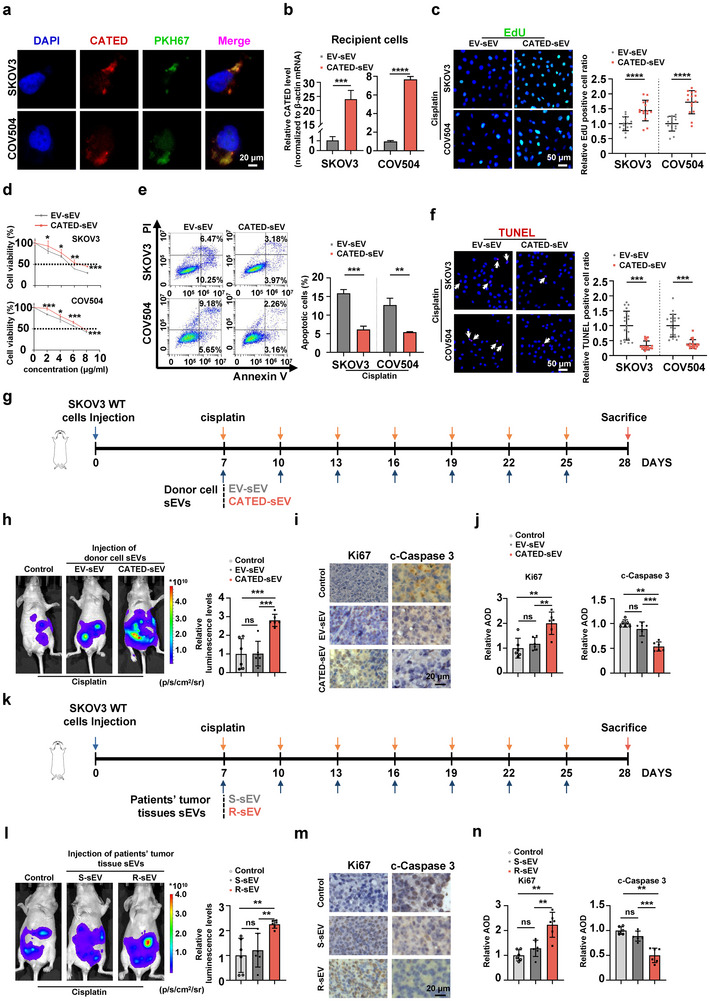
sEV‐mediated CATED overexpression enhanced HGSOC platinum resistance. a) Co‐localization visualization using FISH for CATED and IF staining with PKH67 in SKOV3 and COV504 cells. CATED (red) was co‐localized with PKH67‐labeled sEVs (green). Nuclei are stained with DAPI (blue). Scale bar: 20 µm. PKH67 was used as a fluorescent dye for labeling and visualizing sEVs. b) Evaluation of the efficiency of sEV‐mediated CATED overexpression after 24 h of incubation with recipient cells. CATED‐sEV: sEV‐mediated CATED overexpression; EV‐sEV: sEV‐mediated corresponding empty control. c) EdU assays of SKOV3 and COV504 cells overexpressed CATED mediated by sEVs. Scale bar: 50 µm. d) Cell viability was assessed by CCK8 assay following sEV‐mediated CATED overexpression in SKOV3 and COV504 cells. e) Apoptosis analysis was conducted using flow cytometry following sEV‐mediated CATED overexpression in SKOV3 and COV504 cells. f) TUNEL staining of CATED overexpression mediated by sEVs. Scale bar: 50 µm. For assays in c, e, f, cells were treated with cisplatin at indicated concentrations for 36 h. g) Schematic illustration of the generation of an intraperitoneal tumor‐bearing mouse ovarian cancer model, followed by administration of sEVs from donor cells. h) Representative images (left) and quantification (right) of the xenografts intraperitoneally established by SKOV3 WT cells with luciferase and injected with sEVs from CATED overexpression and control cell cultures. The mice were administered with indicated sEVs and cisplatin every 3 days (n = 6 mice per group). The control group was only injected with cisplatin. i, j) IHC analysis of the specified markers in tumors resected from each group, with quantification presented in the bar figures, is illustrated with a scale bar of 20 µm. k) Schematic illustration of the generation of an intraperitoneal tumor‐bearing mouse ovarian cancer model, followed by administration of sEVs from tumor tissues. S, sensitive; R, resistant. l) Representative images (left) and quantification (right) of the xenografts established intraperitoneally using SKOV3 WT cells with luciferase injected with sEVs derived from patients’ tumor tissues. The mice received the indicated sEVs and cisplatin every 3 days (n = 6 mice per group). The control group was only injected with cisplatin. m,n) IHC analysis of the specified markers in tumors excised from each group is reported, along with quantification represented in the accompanying bar figures. Scale bar: 20 µm. Dated are shown as mean ± SD. *p* values were determined with two‐tailed Student's *t*‐test. *p < 0.05, **p < 0.01, ***p < 0.001, ns. Not significant. All data were from at least three repeats.

To further verify that the CATED‐pro‐resistant effects could be transferred via sEVs, we first isolated and purified sEVs from the supernatant of CATED‐overexpressing stable SKOV3 and COV504 cells (donor cells), as previously described (Figure S4a, Supporting Information).^[^ ^8^ ^]^ The quality of these sEVs, as well as that of control cells, was characterized by TEM (Figure S4b, Supporting Information). The CATED levels in sEVs were validated (Figure S4c, Supporting Information). The sEVs were then incubated with SKOV3 and COV504 WT cells (recipient cells). The sEVs were fluorescently visualized using PKH67, a fluorescent dye used to label the sEVs. The distribution of PKH67 was simultaneously observed using FISH to visualize CATED. Green fluorescently labeled sEVs were shown to be absorbed by recipient cells, with co‐localization of PKH67 and CATED FISH signals, demonstrating that CATED was successfully transferred to recipient cells by donor cell sEVs (**Figure** **3**a; Figure S4a, Supporting Information). Levels of CATED in recipient cells were also measured, and significantly increased levels of CATED were validated compared to those in the empty control group (Figure 3b). These results indicated that CATED could be packaged into sEVs and transferred from donor‐overexpressing cells to wildtype (WT) recipient cells.

To investigate whether sEV‐mediated transfer of CATED promotes cisplatin resistance, we assessed the proliferation and apoptosis of recipient cells after incubation with sEVs from CATED‐overexpressing donor cells and empty control donor cells. After cisplatin treatment, EdU and CCK8 assays showed that sEV‐mediated CATED overexpression significantly promoted the proliferation of recipient cells (Figure 3c,d), while apoptotic cells were decreased as evaluated by FCM and TUNEL assays (Figure 3e,f), compared to the empty control group. Therefore, we investigated the role of sEV‐mediated CATED transfer in cisplatin resistance in vivo. SKOV3 WT cells were first injected intraperitoneally into nude mice, and the mice were treated with isolated sEVs of SKOV3 CATED‐overexpressing stable cells or empty control SKOV3 cells, with cisplatin treatment every 3 days (Figure 3g). The results showed that CATED levels were significantly elevated in the tumors of the group treated with isolated sEVs of SKOV3 CATED‐overexpressing stable cells compared with the empty control group and the tumors progressed significantly, indicating that in sEV‐transmitted CATED enhanced cisplatin resistance (Figure 3h; Figure S4d,e, Supporting Information). Consistently, Ki‐67 levels were increased in the sEV‐mediated CATED overexpression group while cleaved‐Caspase 3 levels were decreased (Figure 3i,j). To further validate these effects, we utilized sEVs derived from clinical tumor tissues of HGSOC patients for subsequent treatment. SKOV3 WT cells were injected intraperitoneally into nude mice, which were subsequently treated with sEVs isolated from the tumor tissues of platinum‐resistant or platinum‐sensitive HGSOC patients along with cisplatin treatment (Figure 3k). CATED levels were preliminarily verified, and the CATED levels in sEVs derived from platinum‐resistant tumor tissues were significantly higher than those in sEVs from platinum‐sensitive tissues (Figure S4f, Supporting Information). Consistent with previous findings, we observed that tumors in the group treated with sEVs derived from platinum‐resistant HGSOC tumors progressed significantly compared with those in the group treated with sEVs from platinum‐sensitive tissues (Figure 3l; Figure S4g, Supporting Information). CATED levels were significantly higher in tumors from the group treated with sEVs derived from platinum‐resistant HGSOC (Figure S4h, Supporting Information), and Ki‐67 levels were also elevated in this group, while cleaved‐Caspase 3 levels were reduced (Figure 3m,n). Taken together, our findings demonstrated that the pro‐resistance effects exerted by CATED can be transferred via sEVs both in vitro and in vivo.

### 2.4 CATED Interacts with DHX36 Protein

**Figure 4:**
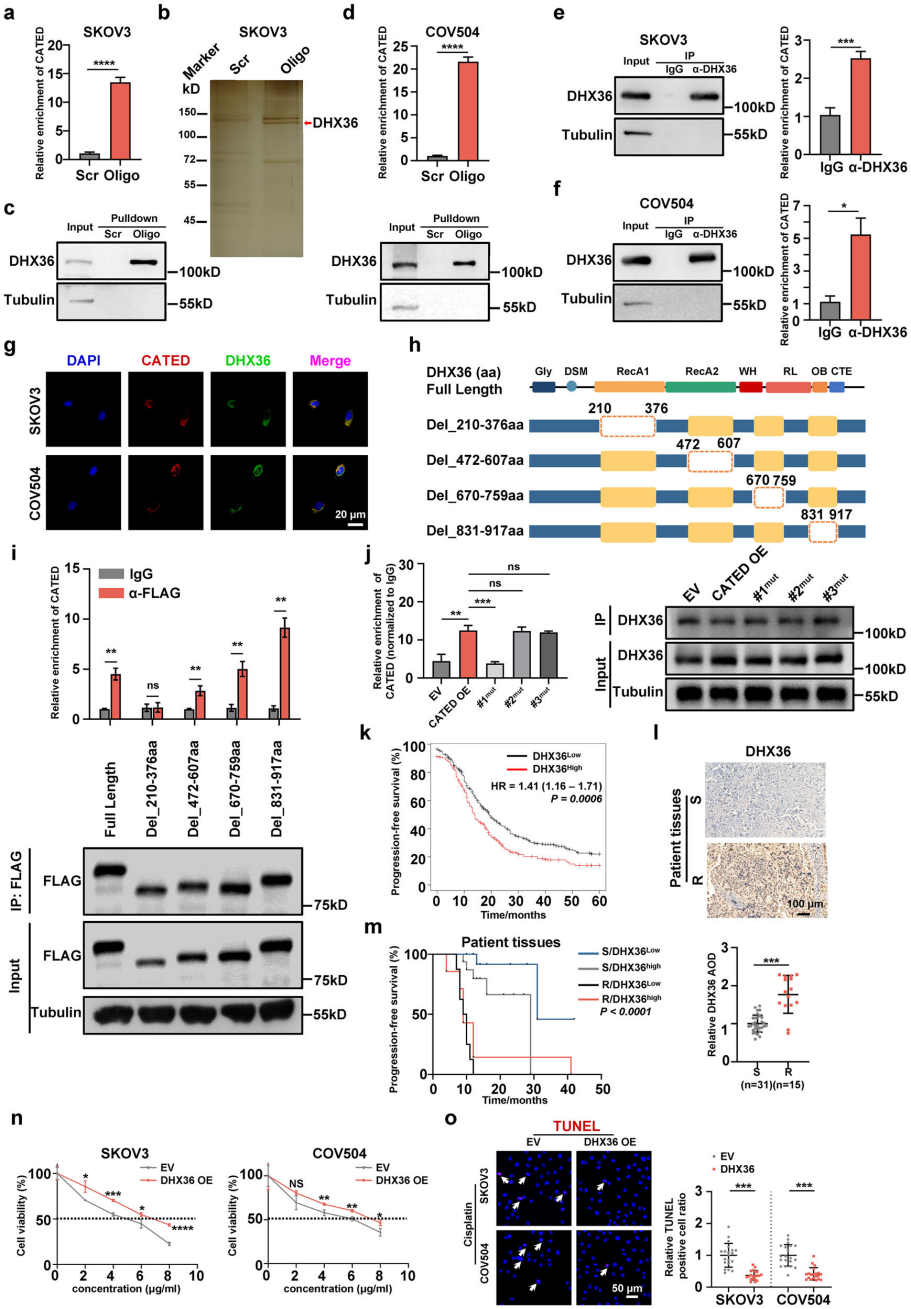
CATED interacts with DHX36. a) Pulldown efficiency of CATED in the SKOV3 cells. Scr: represents the negative control using a biotin‐labeled oligo with scrambled sequences; Oligo: represents the biotin‐labeled oligo with antisense sequences to the CATED. b) Silver staining reveals proteins co‐pulled down, with the red arrow highlighting the band identified as DHX36 by MS (SKOV3). c) Western blot analysis of DHX36 identified that RNA pulldown of CATED co‐pulls down DHX36 in the cells. d) Pulldown efficiency of CATED in the COV504 cells (up). Western blot analysis of DHX36 identified that RNA pulldown of CATED co‐pulls down DHX36 in the cells (low). e,f) RIP with an antibody against DHX36 in the SKOV3 and COV504 cells pull down CATED. Western blots showing efficient pulldown of DHX36. RT‐qPCR analyzes show the enrichment of RIP RNAs. g) Representative images of CATED FISH together with DHX36 IF in SKOV3 and COV504 cells. Scale bar = 20 µm. h) Schematic diagram illustrating the full‐length DHX36 and various truncated forms of DHX36, with dotted rectangles indicating the truncated domains. i) Association of CATED was examined using RT‐qPCR in conjunction with RIP of FLAG‐tagged full‐length and truncated forms of DHX36 in SKOV3 cells. The presence of full‐length and truncated DHX36 was verified by Western blotting with an anti‐FLAG antibody, with tubulin serving as a loading control for the Western blot. j) DHX36 RIP in the SKOV3 cells for CATED and its mutants. Western blots show the RIP efficiency of DHX36. RT‐qPCR analysis shows the enrichment of CATED and its mutants, normalizing to IgG. k) Kaplan–Meier analysis of progression‐free survival for patients collected from the Kaplan‐Meier Plotter Database. l) Employing IHC to evaluate and quantify the expression levels of DHX36 in tumor tissues derived from platinum‐sensitive (n = 31) and platinum‐resistant (n = 15) patients. m) Kaplan–Meier analysis of progression‐free survival for 46 HGSOC patients. n) Cell viability was evaluated using the CCK8 assay following DHX36 overexpression in SKOV3 and COV504 cells. o) TUNEL staining was performed on cells with DHX36 overexpression following treatment with cisplatin. Scale bar: 50 µm. Dated are shown as mean ± SD. *P* values were determined with two‐tailed Student's *t*‐test or log‐rank test. *p < 0.05, **p < 0.01, ***p < 0.001, ****p < 0.0001, ns. Not significant. All data were from at least three repeats.

Next, we investigated the functional mechanisms of CATED by exploring its potential interacting molecules. We first assessed whether CATED functioned as miRNA sponge owing to its cytoplasmic localization by the RNA immunoprecipitation (RIP) of AGO2, which is crucial for facilitating the interaction between the target RNA and miRNA.^[^ ^18^ ^]^ The results demonstrated no enrichment of CATED from AGO2 RIP in SKOV3 cells (Figure S5a, Supporting Information). We have predicted that CATED would have no translational potential (Figure S2d–f, Supporting Information). To investigate whether CATED functions by interacting with RNA‐binding proteins (RBPs), we performed an RNA pulldown assay with a biotin‐labeled oligo against CATED, where CATED was effectively pulled down (**Figure** **4**a) and proteins co‐pulled down with CATED were separated using SDS‐PAGE followed by silver staining and mass spectrometry (MS) (Figure 4b). Using silver staining and western blotting, DHX36, also known as RHAU or G4R1,^[^ ^19^ ^]^ was found to interact with CATED (Figure 4b,c; Figure S5b, Table S4, Supporting Information). This interaction was further validated in COV504 cells (Figure 4d). AGO2 was not detected in the precipitates, further confirming that CATED did not function as miRNA sponge (Figure S5c, Supporting Information). The DHX36 RIP assay further validated the interaction between CATED and DHX36 in the SKOV3 and COV504 cells (Figure 4e,f). CATED FISH combined with immunofluorescence (IF) against DHX36 revealed that CATED co‐localized with DHX36 in the cytoplasm (Figure 4g). To determine the critical DHX36 region responsible for its interaction with CATED, we used catRAPID, a tool for predicting the protein‐interacting region with RNAs, in which four regions of DHX36 were predicted to potentially interact with CATED (Figure 4h). Thus, full‐length FLAG‐tagged DHX36, together with the four truncated forms, were overexpressed in SKOV3 cells, followed by FLAG RIP. CATED enrichment results revealed that all but truncation in the 210–376 aa (amino acids) in the RecA1 domain of DHX36 enriched CATED, indicating that the 210–376 aa of DHX36 might be indispensable for CATED binding (Figure 4i). To determine the binding sites of DHX36 on CATED, we have analyzed the DHX36 PAR‐CLIP data (GSE105171) from HEK293 cells, as most RBP CLIP signals are preserved across cell types for similarly expressed genes.^[^ ^20^ ^]^ We first identified the universal DHX36 binding motifs on RNAs (Figure S5d, Supporting Information). Three DHX36 binding sites on CATED were identified, and these motifs were then mapped to these binding sites, respectively (Figure S5e, Supporting Information). We thus mutated these motifs in the respective three binding sites, and results showed that mutation of Motif 3, 8, and 6 in #1 binding site significantly decreased the interaction between CATED and DHX36 (Figure 4j; Figure S5e, Supporting Information), indicating that these motifs in #1 binding site were necessary for the binding DHX36 to CATED.

We then analyzed the clinical relevance of DHX36. The Kaplan‐Meier Plotter Database demonstrated that high levels of DHX36 protein were associated with reduced PFS (Figure 4k). DHX36 IHC analysis of HGSOC tissues from our 46 HGSOC patient's cohort showed that DHX36 levels were significantly higher in platinum‐resistant patients than in platinum‐sensitive patients (Figure 4l), and PFS deduced from our 46 HGSOC patient’ cohort also confirmed this trend (Figure 4m). Subsequently, we overexpressed DHX36 in the SKOV3 and COV504 cells (Figure S5f, Supporting Information). Functional experiments demonstrated that DHX36 overexpression promotes cell proliferation and inhibits apoptosis (Figure 4n,o).

### 2.5 CATED Facilitates PIAS1‐Mediated SUMOylation of DHX36

**Figure 5:**
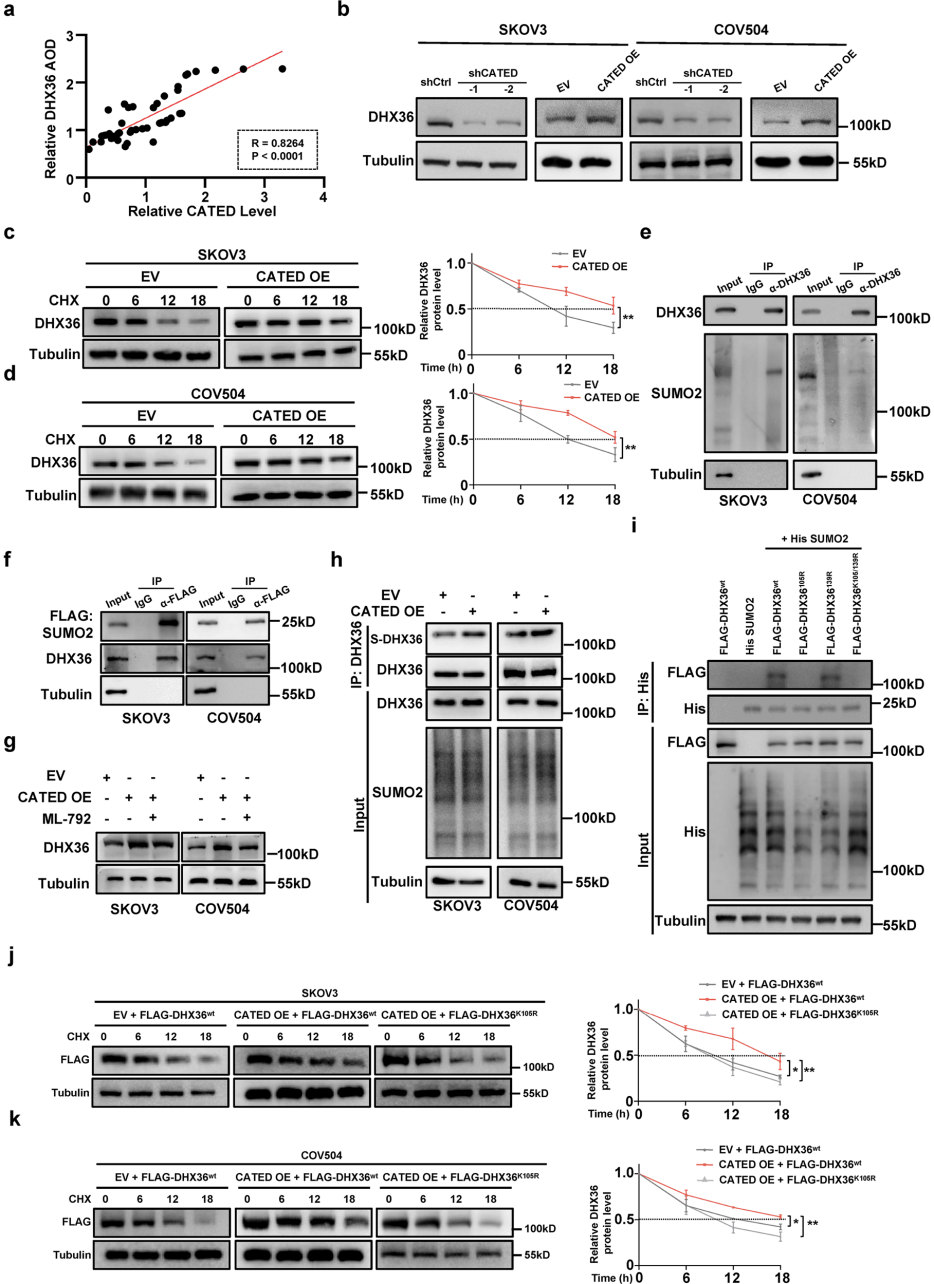
CATED promoted the stability of DHX36 through SUMOylation. a) Correlation coefficient analysis of CATED and DHX36 expression was conducted, with the Pearson correlation coefficient (R) and *p*‐value (*p*) reported. b) DHX36 protein levels were assessed by Western blot analysis in CATED overexpression and knockdown cells. c,d) CHX assay of DHX36 protein stability. CATED‐overexpressing cells were treated with CHX (100 µg mL^−1^) and harvested at specified time points for Western blot analysis. The band intensity of DHX36 was quantified from three independent replicates, with the intensity at 0 h normalized to 1. CHX, Cycloheximide. e) Co‐IP assay utilizing anti‐DHX36 was conducted to evaluate the conjunction of SUMO2 on DHX36 in SKOV3 and COV504 cells. f) Co‐IP assay using anti‐FLAG was performed to assess the conjunction of FLAG‐labeled SUMO2 on DHX36 in cells. g) DHX36 protein levels were assessed by Western blot analysis in CATED overexpression alone or with ML‐792 (MCE, HY‐108702). h) Co‐IP assay using anti‐DHX36 was performed to assess the conjunction of SUMO2 on DHX36 in cells after CATED overexpression. S‐DHX36: SUMOylated DHX36. i) WT and mutants of DHX36 with His‐SUMO2 were transfected into SKOV3 cells as indicated. SUMO2 conjugated DHX36 proteins were immunoblotted. K, lysine; R, arginine; WT, wild type. j,k) CHX assay of FLAG‐tagged DHX36 protein and its mutant stability upon CATED‐overexpressing in SKOV3 and COV504 cells. CHX, Cycloheximide. *p* values were determined with ANOVA test. *p < 0.05, **p < 0.01. All data were from three repeats.

To further elucidate the reciprocal regulation of the CATED‐DHX36 interaction, a correlation coefficient was calculated, which showed a positive association between the DHX36 protein levels and CATED (**Figure** **5**a). Overexpression or knockdown of DHX36 did not affect the CATED levels (Figures S5f, and S6a–c, Supporting Information). In contrast, CATED overexpression increased DHX36 protein levels, whereas CATED knockdown decreased DHX36 protein levels (Figure 5b). DHX36 mRNA levels were not affected by changes in CATED levels (Figure S6d,e, Supporting Information). Given that both CATED and DHX36 play roles in the proliferation and apoptosis of HGSOC (Figures 2 and 3; Figure 4n,o), we investigated whether CATED exerts its pro‐resistance functions via DHX36. Knockdown of DHX36 eliminated the effects of CATED on the promotion of cell proliferation and inhibition of apoptosis (Figure S6f–h, Supporting Information), indicating that CATED is involved in cisplatin resistance by interacting with DHX36 in HGSOC.

We then focused on how CATED promoted DHX36 protein levels. Cellular protein stability and degradation are orchestrated by post‐translational regulation, such as the ubiquitin‐proteasome system.^[^ ^21^ ^]^ We first investigated whether levels of DHX36 protein were regulated by ubiquitination and whether this regulation was modulated by CATED. Our findings demonstrated that DHX36 could be subjected to ubiquitin‐mediated regulation (Figure S7a, Supporting Information). However, modulation of CATED levels did not appear to affect the DHX36 ubiquitination status (Figure S7b, Supporting Information). We thus sought to investigate whether CATED regulated DHX36 levels by stabilizing its protein. Overexpression of CATED significantly increased the half‐life of DHX36 protein in SKOV3 and COV504 cells, suggesting that CATED could stabilize DHX36 protein (Figure 5c,d). To investigate how CATED regulates the stability of DHX36 protein, we performed co‐immunoprecipitation (co‐IP) with anti‐DHX36 antibody followed by MS, and several SUMOylation‐related proteins were detected (eg. SUMO2, SAE1, UBA2) (Figure S7c, Table S5, Supporting Information). SUMOylation is known to promote protein stability.^[^ ^22^, ^23^ ^]^ To validate whether the CATED‐promoted DHX36 protein levels were dependent on SUMOylation‐mediated stabilization, we conducted a co‐IP assay with an anti‐DHX36 antibody and observed that SUMO2 specifically covalently bound to DHX36 using western blotting with an anti‐SUMO2 antibody (Figure 5e). Consistently, DHX36 co‐immunoprecipitated with FLAG‐SUMO2 (Figure 5f). To further verify whether CATED increased DHX36 levels by promoting SUMOylation, cells were treated with ML‐792, a highly selective and potent SUMO E1 enzyme inhibitor,^[^ ^24^ ^]^ where the increase in DHX36 levels promoted by CATED overexpression was abolished by ML‐792 (Figure 5g). DHX36 co‐IP assays demonstrated that overexpression of CATED enhanced the SUMO2 conjugation of DHX36 (S‐DHX36), indicating that CATED promoted SUMOylation of DHX36 (Figure 5h). To determine the potential SUMOylation site (s) on DHX36, GPS‐SUMO,^[^ ^25^ ^]^ a tool for analyzing SUMOylation sites, was employed to predict the putative SUMOylation sites of DHX36. Two lysine residues, K105 and K139, predicted in silico^[^ ^26^ ^]^ and reported as SUMOylated sites by SUMO2 were also predicted by GPS‐SUMO. We mutated these two sites for subsequent analysis (Figure S7d–f, Supporting Information). Co‐IP assays demonstrated that only the mutation of lysine 105 to arginine (K105R) markedly inhibited DHX36 SUMOylation, whereas mutations at the other site (K139R) had no such effect (Figure 5i; Figure S7g, Supporting Information). Furthermore, we observed that CATED overexpression failed to maintain the stability of the FLAG‐tagged K105R‐mutated DHX36 variant, demonstrating that CATED stabilizes DHX36 through K105 SUMOylation (Figure 5j,k).

**Figure 6:**
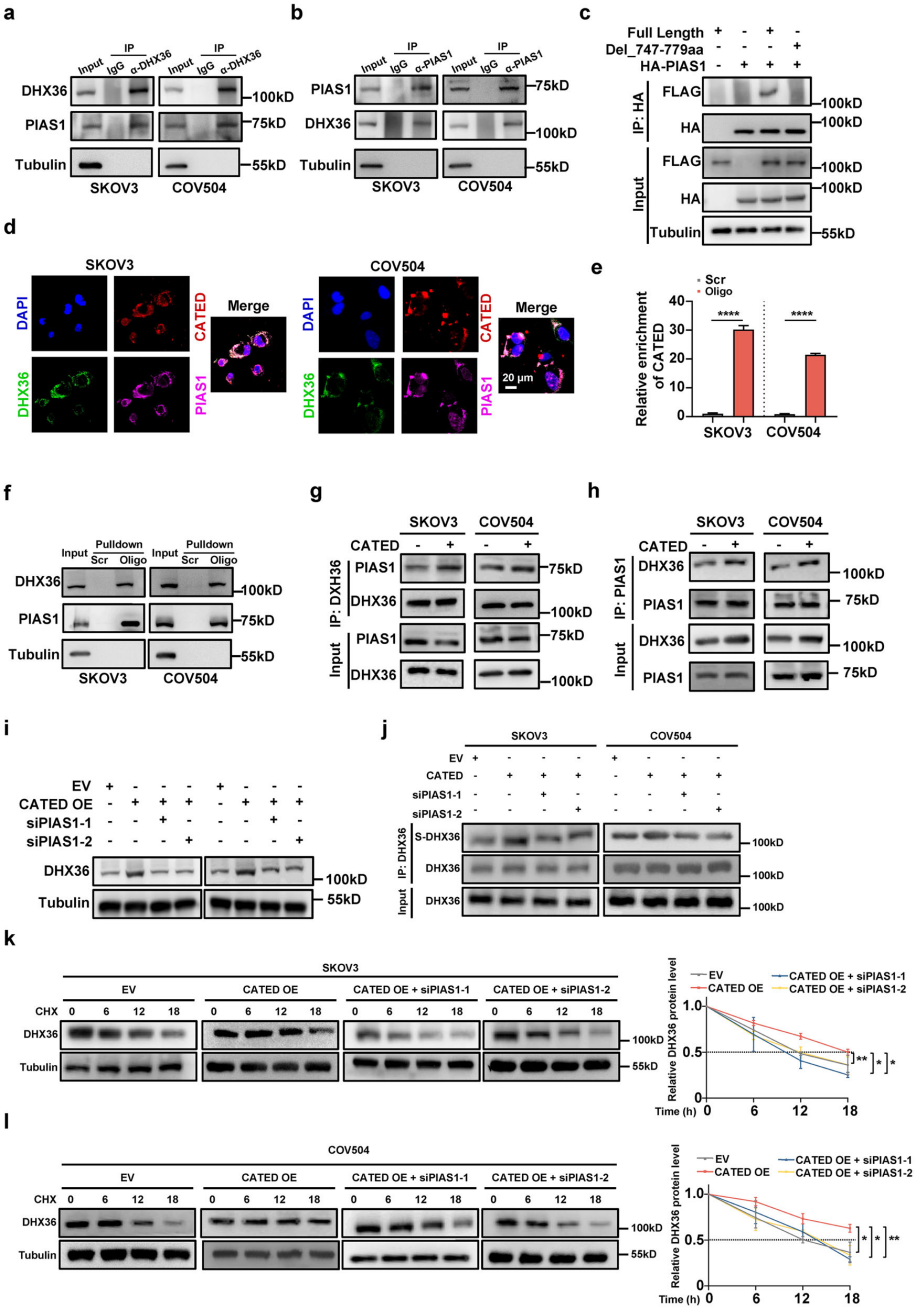
CATED serves as a scaffold in the interaction between PIAS1 and DHX36. a,b) Interaction between PIAS1 and DHX36 was examined by co‐IP using anti‐DHX36 antibody and anti‐PIAS1 antibody. c) WT and truncated mutant of DHX36 with HA‐PIAS1 were transfected into SKOV3 cells as indicated. Co‐IP assays using an anti‐HA antibody followed by FLAG‐tagged DHX36 proteins were immunoblotted. d) Representative images of CATED FISH together with DHX36 and PIAS1 IF in SKOV3 and COV504 cells. Scale bar = 20 µm. e,f) CATED pulldown of PIAS1 in SKOV3 and COV504 cells was performed. The efficiency of CATED pulldown is depicted in the bar figure. The Western blot results of the pulled‐down PIAS1 are presented. g) Co‐IP assay using anti‐DHX36 was performed to detect PIAS1 levels upon CATED overexpression. h) Co‐IP assay using anti‐PIAS1 was performed to detect DHX36 levels upon CATED overexpression. i) DHX36 protein levels were analyzed by Western blot in the context of CATED overexpression, with or without PIAS1 knockdown. j) SUMO2 conjugation of DHX36 protein levels was analyzed by Western blot upon CATED overexpression, with or without PIAS1 knockdown. k,l) CHX assay of DHX36 protein was assessed after CATED overexpression with or without PIAS1 knockdown, in SKOV3 and COV504 cells. Dated are shown as mean ± SD. *p* values were determined with two‐tailed Student's *t*‐test or ANOVA test. *p < 0.05, **p < 0.01. All data were from three repeats.

To further identify the DHX36‐specific SUMO E3 ligase, we found that PIAS1 co‐immunoprecipitated with DHX36 in the aforementioned MS results (Figure S8a; Table S5, Supporting Information). Co‐IP assays, followed by western blotting demonstrated that DHX36 and PIAS1 could be mutually co‐immunoprecipitated (**Figure** **6**a,b), indicating that PIAS1 could potentially mediate DHX36 SUMOylation. To identify the critical domain of DHX36 for its interaction with PIAS1, we predicted the potential binding sites via the AlphaFold 3 server, and the residues 747, 772, and 779 aa located at the ratchet‐like (RL) domain of DHX36 are predicted critical for DHX36‐PIAS1 binding (Figure S8b, Supporting Information). To validate the prediction, we constructed the DHX36 truncation in the 747–779 aa, and FLAG co‐IP assay in SKOV3 cells showed that the 747–779 aa deletion failed to co‐IP PIAS1 (Figure 6c), indicating that 747–779 aa region at the RL domain of DHX36 is critical for its interaction with PIAS1.

Since DHX36 can be SUMOylated, and this modification is enhanced by CATED, we aimed to further elucidate the mechanism by which CATED promotes DHX36 SUMOylation. Overexpression of CATED had little or no impact on the relative mRNA and protein levels of MS‐detected SUMOylation‐associated proteins (Figure S8c–e, Supporting Information). IF of proteins and CATED FISH showed co‐localization of CATED, DHX36, and PIAS1 (Figure 6d). We then investigated whether CATED acted as a scaffold to facilitate efficient SUMOylation of PIAS1 at its target. CATED pulldown analysis revealed that CATED interacted with PIAS1, and PIAS1 RIP assay also significantly enriched CATED (Figure 6e,f; Figure S8f,g, Supporting Information). Co‐IP of DHX36 revealed that CATED overexpression enhanced the recruitment of PIAS1. Conversely, co‐IP of PIAS1 showed that CATED overexpression also promoted the interaction of PIAS1 with DHX36, indicating that CATED acts as a scaffold to facilitate the interaction between DHX36 and PIAS1 (Figure 6g,h). PIAS1 knockdown suppressed the CATED‐induced upregulation of DHX36 protein (Figure 6i; Figure S8h, Supporting Information). Additionally, PIAS1 knockdown reversed the CATED‐mediated DHX36 SUMOylation (Figure 6j) and attenuated the effects of CATED overexpression in the stabilization of DHX36 protein (Figure 6k,l). Collectively, these results indicate that CATED might function as a scaffold for DHX36 and PIAS1 to facilitate the efficient PIAS1‐mediated SUMOylation of DHX36, which in turn stabilizes and upregulates the DHX36 protein to promote HGSOC cisplatin resistance.

### 2.6 DHX36 Binds to the mRNA of RAP1A

**Figure 7:**
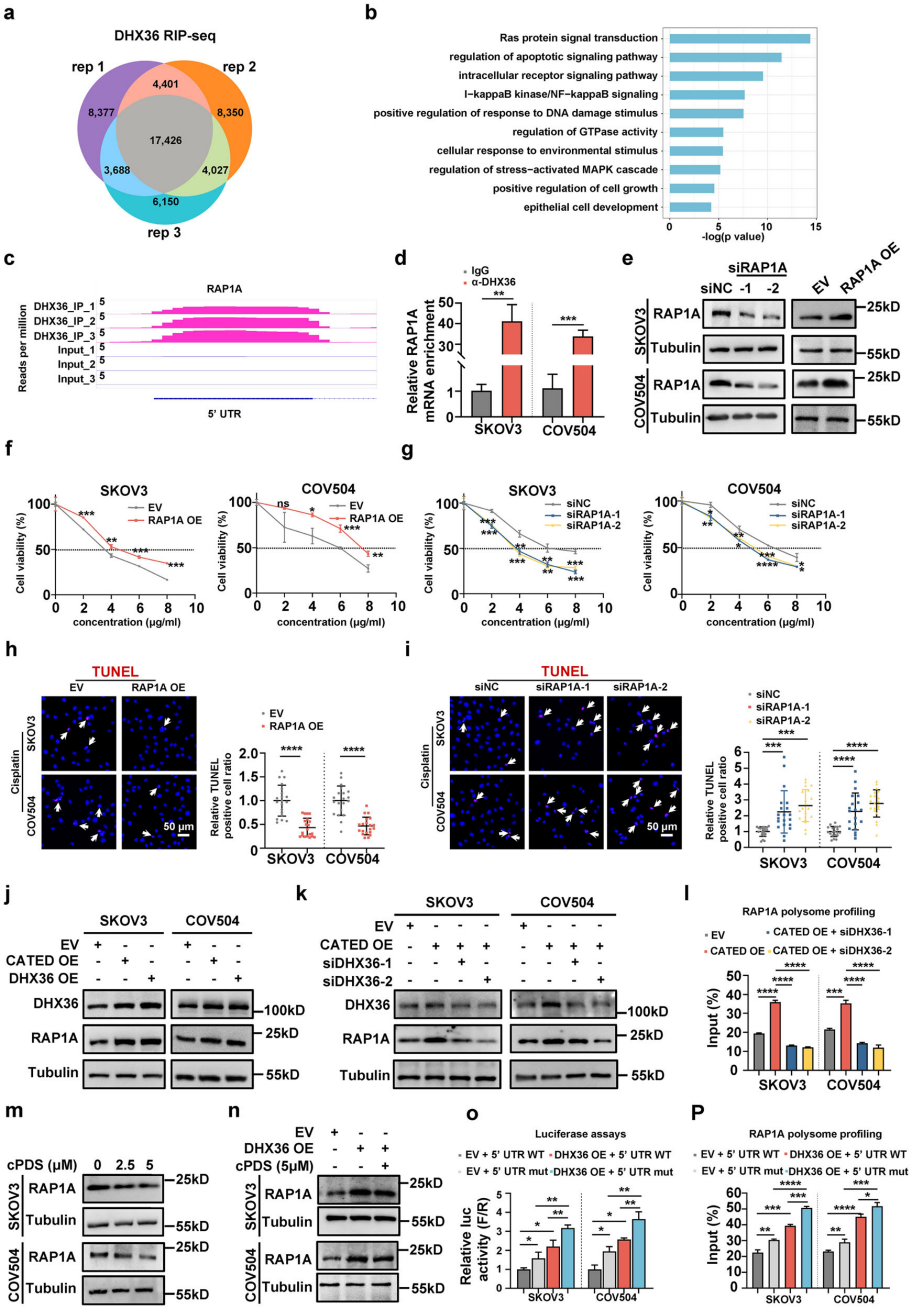
RAP1A is the downstream target of the CATED‐DHX36 axis. a) A large number of identified binding sites (17,426) were shared in the three biological replicates. Rep, replicates. b) Results of the GO analysis for all genes bound by DHX36. c) DHX36 binding signals on RAP1A's 5′ UTR in SKOV3 cells. d) RT‐qPCR analysis showed the enrichment of RAP1A mRNA in RIP using an antibody against DHX36. e) Validation of RAP1A overexpression and knockdown efficiency in SKOV3 and COV504 cells. NC: normal control. f,g) Cell viability was evaluated using the CCK8 assay following RAP1A overexpression or knockdown in SKOV3 and COV504 cells. h,i) TUNEL staining was performed after RAP1A overexpression or knockdown, along with cisplatin treatment in SKOV3 and COV504 cells. Scale bar: 50 µm. j) DHX36 and RAP1A protein levels were analyzed by Western blot in cells with either CATED or DHX36 overexpression. k) DHX36 and RAP1A protein levels were assessed by Western blot in cells with CATED overexpression, with or without DHX36 knockdown. l) Polysome profiling in cells with CATED overexpression, with or without DHX36 knockdown, was performed using sucrose gradient fractionation. RAP1A mRNA translation efficiency was analyzed based on its distribution in the LMW and HMW fractions in cells. m) SKOV3 and COV504 cells were treated with 2.5 or 5 µM cPDS, and RAP1A protein levels were assessed by Western blot in the same cells. n) RAP1A protein levels were examined by Western blot analysis in DHX36 overexpression alone or with cPDS. o) A dual‐luciferase assay was performed to evaluate the effect of DHX36 on the translation efficiency of a transcript containing the RAP1A 5’ UTR. The 5’ UTR of RAP1A mRNA or its mutation form was used as the 5’ UTR of firefly luciferase mRNA, with the corresponding DHX36 protein overexpressed. p) Polysome profiling was performed to analyze the translation of RAP1A mRNA in the context of RAP1A 5' UTR mutations, with overexpression of the corresponding DHX36 protein. Cells overexpressing RAP1A contained either the wild‐type (WT) or mutant 5' UTR of RAP1A. Dated are shown as mean ± SD. *P* values were determined with two‐tailed Student's *t*‐test. *p < 0.05, **p < 0.01, ***p < 0.001, ****p < 0.0001. All data were from three repeats.

To gain deeper insights into the molecular mechanisms of CATED‐DHX36 in HGSOC platinum resistance, we conducted a RIP assay using DHX36 antibody targeting endogenously expressed DHX36 in SKOV3 cells, followed by RNA‐seq (RIP‐seq) analysis of the co‐precipitated RNAs (Figure S9a, Supporting Information). Among the 17,426 overlapping peaks across the three RIP replicates (**Figure** **7**a), most of the binding peaks (15,550, 89.23%) were mapped to protein‐coding mRNAs (Figure S9b, Supporting Information). The mRNA peaks were mostly mapped in coding sequences (CDS), followed by the 3’ UTR, intron, and 5’ UTR (Figure S9c, Supporting Information). DHX36 signals were detected at CATED, with peaks mostly appearing at exon 1, consistent with our analysis of PAR‐CLIP and RNA motif mutation validation (Figure 4j; Figures S5e and S9d, Supporting Information). Gene ontology (GO) analysis of the overlapping genes highlighted most enrichment in pathways such as “Ras protein signal transduction,” “regulation of apoptotic signaling pathway,” “positive regulation of response to DNA damage stimulus” and “regulation of stress‐activated MAPK cascade” (Figure 7b). Notably, RAP1A in “Ras protein signal transduction,” a GTPase from the Ras superfamily that alternates between an inactive GDP‐bound state and an active GTP‐bound state, was implicated in multiple other pathways in our analysis, including “regulation of GTPase activity” and “epithelial cell development.” Analysis of the RIP‐seq data also revealed that the DHX36 signal was highly enriched at the 5’ UTR of RAP1A mRNA (Figure 7c), consistent with a previous report that DHX36 regulates mRNA translation or destabilization by binding to the 5’ UTR or 3’ UTR.^[^ ^19^, ^27^ ^]^ DHX36 RIP experiments revealed that DHX36 effectively captured the RAP1A mRNA (Figure 7d; Figure S9e, Supporting Information). We then evaluated the functional roles of RAP1A in HGSOC platinum resistance by modulating RAP1A levels through either overexpression or knockdown (Figure 7e). Overexpression of RAP1A enhanced cell proliferation and inhibited apoptosis in SKOV3 and COV504 cells upon cisplatin treatment, whereas knockdown of RAP1A exhibited the opposite effects (Figure 7f–i). These results demonstrated that RAP1A may function as a downstream target of the CATED‐DHX36 axis in HGSOC platinum resistance.

To examine whether CATED‐DHX36 regulated the mRNA and protein levels of RAP1A, we found that overexpression of either CATED or DHX36 resulted in an increase in the protein levels of RAP1A, but did not affect its mRNA levels (Figure 7j; Figure S9f, Supporting Information). Additionally, siRNA knockdown of DHX36 abolished the promoting effect of CATED on RAP1A protein levels but not the RAP1A mRNA level, suggesting that CATED regulates RAP1A protein levels by upregulating DHX36 (Figure 7k; Figure S9g, Supporting Information). We also observed that the effects of CATED in promoting cell proliferation and inhibiting apoptosis were abolished by RAP1A siRNA knockdown (Figure S9h–l, Supporting Information). Taken together, these results demonstrate that RAP1A is the downstream target of the CATED‐DHX36 functional axis, and elevated DHX36 enhances RAP1A translation by unwinding rG4 structures, leading to increased cell proliferation and reduced apoptosis.

Considering that DHX36 binds to the 5’ UTR of RAP1A mRNA without altering its mRNA levels, and combining this with a previous report that DHX36 functions as a post‐transcriptional or translational regulator by binding to and unwinding RNA G‐quadruplex (rG4) structures,^[^ ^27^ ^]^ we next explored whether DHX36 regulates RAP1A translation by binding to and unwinding the rG4 structures in the RAP1A 5’ UTR. Polysome profiling for RAP1A mRNA translation efficiency revealed that CATED overexpression promoted RAP1A translation, whereas DHX36 knockdown reversed the promoting role of CATED in RAP1A translation (Figure 7l; Figure S10a, Supporting Information). G4Hunter, a G4 prediction tool, was used to identify the potential rG4 structures in the 5’ UTR of RAP1A (Figure S10b, Supporting Information). The protein levels of RAP1A were significantly decreased when cells were treated with cPDS (carboxyPDS, a derivative of PDS that stabilizes rG4),^[^ ^27^ ^]^ whereas the mRNA levels were slightly elevated, consistent with a previous report (Figure 7m; Figure S10c, Supporting Information). Additionally, treatment with cPDS attenuated the effects of DHX36 on promoting RAP1A protein levels (Figure 7n), suggesting that DHX36 may enhance RAP1A levels by unwinding rG4 structures to facilitate translation. To further validate these results, we mutated the rG4 structure sequences located in the 5’ UTR (Figure S10d, Supporting Information). Using the 5’ UTR of RAP1A mRNA as the firefly luciferase mRNA 5' UTR in a dual‐luciferase reporter assay, with or without rG4 mutations, we found that DHX36 overexpression enhanced RAP1A protein levels through the rG4 structure. (Figure 7o, Figure S10d,e, Supporting Information). We next constructed RAP1A overexpression vectors with or without RAP1A 5’ UTR mutation, and polysome profiling revealed that DHX36 overexpression enhanced RAP1A translation upon RAP1A 5’ UTR mutation, suggesting that DHX36 promoted RAP1A translation by unwinding the rG4 structure in its 5' UTR (Figure 7p; Figure S10d,f, Supporting Information). These results demonstrated that DHX36 could promote RAP1A translation via unwinding rG4 structure in the 5’ UTR of RAP1A mRNA.

### 2.7 CATED‐DHX36‐RAP1A Activates the MAPK Pathway to Promote Platinum‐Resistance

**Figure 8:**
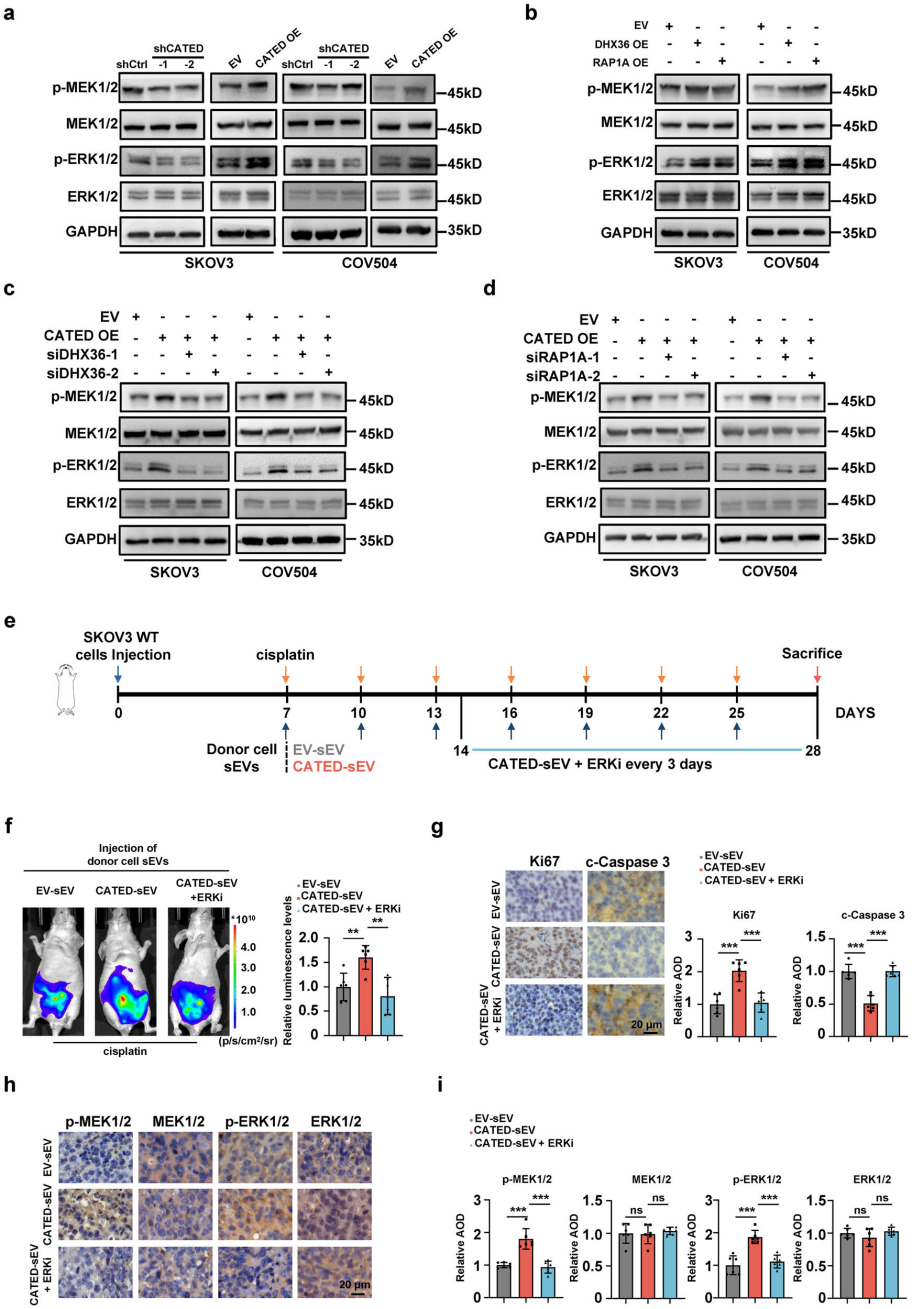
CATED activated MAPK pathway both in vitro and in vivo. a–d) Levels of p‐MEK1/2, MEK1/2, p‐ERK1/2, and ERK1/2 proteins within diverse cell types were investigated. GAPDH was used as a loading control. e) Schematic illustration for generating the intraperitoneal tumor‐bearing mouse ovarian cancer model. ERKi were injected after 2 weeks. ERKi, ERK inhibitors. f) Representative images (left) and quantitative analysis (right) of xenografts established via intraperitoneal injection using SKOV3 WT cells with luciferase. The sEVs derived from donor cells were injected solely or with sEVs accompanied by simultaneous injection of ERKi (six mice in each group). g–i) IHC analysis of the indicated markers in tumors from each group. The quantification of these markers across the different groups is illustrated in the bar figures. Scale bar = 20 µm. Dated are shown as mean ± SD. *p* values were determined with two‐tailed Student's *t*‐test. **p < 0.01, ***p < 0.001, ns. Not significant. All data were from at least three repeats.

To further investigate the downstream effects of the CATED‐DHX36‐RAP1A axis, stable CATED‐overexpressing SKOV3 cells were subjected to RNA‐seq. Differentially expressed genes affected by CATED overexpression were analyzed and subjected to GO analysis, either upregulated or downregulated, where “cellular response to cisplatin,” “cell population proliferation,” and “intrinsic apoptotic signaling pathway in response to DNA damage” are listed. Notably, “regulation of MAPK cascade” was present in both the upregulated and downregulated GO pathways (Figure S11a, Supporting Information), and “regulation of stress‐activated MAPK cascade” were also listed in the GO of DHX36 RIP‐seq (Figure 7b). Thus, it is tempting to speculate that the MAPK/ERK signaling pathway may be the downstream pathway that CATED exerts in this context. The results showed that CATED overexpression activated the MAPK/ERK signaling pathway, whereas CATED knockdown exhibited the opposite effects (**Figure** **8**a). Overexpression of DHX36 or RAP1A enhanced the MAPK/ERK signaling pathway activation (Figure 8b), and CATED‐induced activation of the MAPK/ERK pathway was reversed by knockdown of either DHX36 or RPA1A (Figure 8c,d).

To validate that the functions of CATED‐DHX36‐RAP1A in cisplatin resistance were dependent on the MAPK/ERK pathway and that these effects could be transferred by sEVs, we injected sEVs isolated from CATED‐overexpressing stable SKOV3 cells, along with cisplatin treatment, into nude mice, followed by treatment with intraperitoneal injections of ERKi (ERK inhibitor) (Figure 8e). We observed that enhanced tumor progression in the group treated with sEVs isolated form CATED‐overexpressing cells was reversed upon the administration of ERKi (Figure 8f; Figure S11b, Supporting Information), whereas CATED expression, as well as DHX36 and RAP1A levels, were significantly elevated in the CATED‐overexpressing sEV group and were not affected by ERKi (Figure S11c–e, Supporting Information). Mice injected with CATED overexpression sEVs exhibited significantly higher levels of Ki‐67, p‐MEK, and p‐ERK, and markedly decreased cleaved‐Caspase 3 in tumor sections, whereas these effects were reversed by ERKi (Figure 8g–i). Collectively, we demonstrated that MAPK/ERK is the downstream pathway of the CATED‐DHX36‐RAP1A axis, and that these effects could be validated through sEV‐mediated transfer of CATED in vivo.

### 2.8 ASO Targeting CATED Inhibits HGSOC Cisplatin Resistance In Vivo

**Figure 9:**
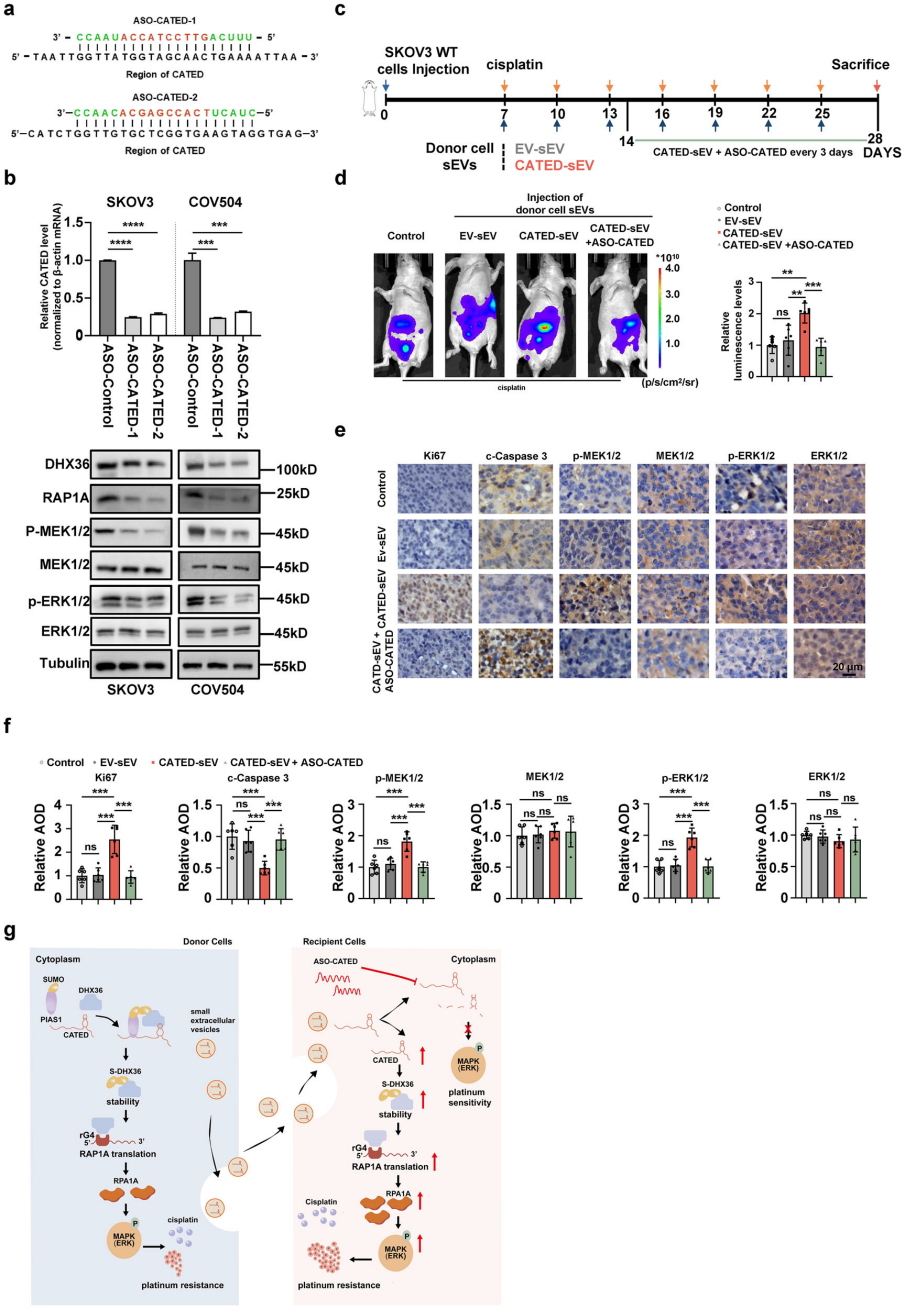
ASO targeting CATED restores cisplatin sensitivity. a) The ASO sequences targeting the CATED. The central DNA sequences of the Gapmer ASO were marked red. b) RT‐qPCR analysis of CATED expression in CATED knockdown cells after ASO‐mediated knockdown of CATED. Protein levels of DHX36, RAP1A, p‐MEK1/2, MEK1/2, p‐ERK1/2, and ERK1/2 were examined by western blotting in CATED knockdown cells, which were treated with ASO‐targeting CATED.ASO‐CATED, ASO‐targeting CATED. c) A schematic illustration for generating the intraperitoneal tumor‐bearing mice ovarian cancer model. ASO targeting CATED were injected after 2 weeks. d) Representative images (left) and quantitative analysis (right) were conducted regarding the xenografts, which were established through intraperitoneal injection of SKOV3 WT cells with luciferase. The SKOV3 WT cells were subjected to either sEVs solely obtained from donor cells or sEVs in combination with simultaneous injection of ASO‐CATED (n = 6 mice per group). The control group was only injected with cisplatin. e,f) IHC analysis was performed for the specified markers within the tumors of each group. Scale bar: 20 µm. The quantification of these markers among the different groups was depicted in the bar graphs. g) A concise summary and a proposed model concerning the roles and functional mechanisms of CATED in ovarian cancer. Dated are shown as mean ± SD. *P* values were determined with a two‐tailed Student's *t*‐test. **p < 0.01, ***p < 0.001, ****p < 0.0001, ns. Not significant. All data were from at least three repeats.

ASO drugs have attracted considerable attention because of their ability to target a diverse array of RNAs with high efficiency, a functionality that has been validated in vivo.^[^ ^28^, ^29^ ^]^ To explore the clinical potential of CATED inhibitors for the treatment of platinum‐resistant HGSOC, we designed two Gapmer ASOs targeting different regions of CATED (**Figure** **9**a), which replaced the central sequence of the ASO with DNA, and were modified by 2‐methoxy ribose and a phosphorothioate backbone to enhance ASO stability and bioactivity. Both ASOs efficiently knocked down CATED in SKOV3 and COV504 cells without affecting the levels of multiple well‐characterized lncRNAs. (Figure 9b; Figure S12a,b, Supporting Information). The levels of DHX36 and RAP1A were significantly decreased and the MAPK‐ERK pathway was inactivated upon ASO‐CATED treatment of SKOV3 and COV504 cells (Figure 9b). Knockdown of CATED using ASO‐CATED also significantly reduced the half‐life of DHX36 protein in SKOV3 and COV504 cells (Figure S12c,d, Supporting Information).

We further investigated the therapeutic effects of ASO‐CATED in nude mice injected with sEVs isolated from stable CATED‐overexpressing SKOV3 cells along with cisplatin treatment (Figure 9c). A pilot assessment revealed that ASO‐CATED‐1 administration yielded sustained suppression of CATED levels in tumor tissues of the mouse intraperitoneal xenograft model (Figure S12e, Supporting Information). Subsequent in vivo experiments demonstrated that ASO‐CATED effectively reversed the pro‐resistance effects in group treated with isolated sEVs of SKOV3 CATED‐overexpressing stable cells, confirming its ability to prevent platinum‐resistance mediated by sEVs in vivo (Figure 9c,d; Figure S12f, Supporting Information). Levels of CATED, DHX36, and RAP1A were significantly decreased in the ASO‐CATED group, while no changes were observed in the levels of other well‐characterized lncRNAs (Figure S12g–j, Supporting Information). Meanwhile, mice injected with ASO‐CATED exhibited significantly lower levels of Ki‐67, p‐MEK, and p‐ERK, and significantly higher levels of cleaved‐Caspase 3 in tumors (Figure 9e,f).

## 3 Discussion

LncRNAs in cancers have been extensively studied,^[^ ^8^, ^30^ ^]^ although a more comprehensive understanding of lncRNAs with significant functions and intricate mechanisms is required. Here, we prove that upregulation of CATED, derived from tumor sEVs, contributes to platinum resistance in HGSOC in vitro and in vivo, and is associated with shorter PFS. These effects can be transferred by sEV‐mediated CATED overexpression in ovarian cancer cells and mouse models. We identified that CATED binds to and upregulates DHX36 through PIAS1‐mediated SUMOylation at the K105 site. Evidence from DHX36 RIP‐seq revealed that RAP1A, which is involved in Ras protein signal transduction, is a downstream target of DHX36. DHX36 enhances RAP1A translation by unwinding rG4 structures, which triggers the MAPK signaling pathway that exerts pro‐resistance functions. Furthermore, targeting CATED expression with a specific ASO restored tumor sensitivity to cisplatin in a DHX36‐RAP1A‐MAPK manner, indicating that CATED may serve as a potential therapeutic target for the treatment of platinum resistance in HGSOC (Figure 9g).

LncRNAs are highly cancer‐type specific compared to protein‐coding genes and the functions of specific lncRNAs may differ according to the various contexts of tumors.^[^ ^31^, ^32^ ^]^ For example, lncRNA H19 is often implicated as an oncogene in multiple cancer types, yet, many conflicting studies have suggested that H19 functions to suppress tumor development.^[^ ^33^ ^]^ We have proved with TCGA data that CATED could be universally expressed in multiple cancer types (Figure S2b, Supporting Information), suggesting CATED might potentially function with similar or distinct mechanisms, which requires future studies to explore its functional diversity. Of note, in a meta‐analysis of available gene expression profiling studies from EOC patients, lncRNA ENSG00000267058 (CATED in this study) was generally downregulated in different EOC cohorts, uncovering the complexity of CATED in different cancer types, including EOC.^[^ ^34^ ^]^ It is reasonable to speculate that CATED may exert its functions as an oncogene or tumor suppressor depending on the different cancer subtypes (eg. HGSOC) and context of cancers such as platinum treatment. Further, lncRNAs are versatile molecules that function through various mechanisms, and this could be due to cancer heterogeneity, and the use of different cancer cell lines and testing conditions. We have also confirmed with evidence that CATED lacks coding potential (Figure S2d–f, Supporting Information), which means CATED largely functions as a noncoding RNA. Other means of validation may also be used to explore its coding potential, such as in vitro transcription and translation assay, to more comprehensively unveil the functional and mechanistic complexity of CATED in the future.

Patients with HGSOC who develop platinum resistance, either due to intrinsic or acquired resistance mechanisms, contribute to poor prognosis.^[^ ^35^ ^]^ There is an urgent need to identify novel targets or biomarkers, as well as the underlying mechanisms for the diagnosis and prognosis of platinum resistance in HGSOC. LncRNAs are implicated in multiple cancer types through diverse mechanisms.^[^ ^36^, ^37^ ^]^ However, sEV‐transmitted lncRNAs with strong functions in platinum resistance are limited. Our findings prove that tumor sEV‐transmitted CATED is closely associated with platinum resistance and poor PFS and provide novel in‐depth mechanistic insights into the role of lncRNAs in modulating platinum resistance in HGSOC. More clinical samples are needed to validate the functions and functional mechanisms of CATED, and more importantly, to facilitate cancer therapeutic research both in pre‐clinical and clinical studies.

Evidence indicates that tumor‐derived sEVs play a critical role in modulating the tumor microenvironment (TME), which is a major source of drug resistance. The sEVs act as potent signaling molecules between cancer cells and surrounding cells, and sEV‐mediated cell‐cell communication is an emerging mechanism underlying drug resistance.^[^ ^38^, ^39^ ^]^ Here, we demonstrated with multiple evidence that utilizing sEVs from donor cells for CATED overexpression or from platinum‐resistant HGSOC patients’ tumors could lead to chemoresistance. Notably, our previous study demonstrated that PLADE, a lncRNA derived from HGSOC ascites sEVs, enhances cisplatin sensitivity by inducing predominant R‐loops to impact cellular homeostasis.^[^ ^8^ ^]^ Compared to those from ascites, sEVs directly isolated from HGSOC tumor tissue are used to provide a more accurate reflection of the pathophysiological conditions,^[^ ^40^ ^]^ as lncRNAs from tumor sEVs are more specific to the TME and its characteristics, while ascites sEVs are more complex and may dilute tumor‐specific signals. In conclusion, this study is one step further to investigate sEV‐transmitted lncRNAs and their associated signaling pathways in the chemoresistance of HGSOC.

The lncRNA‐protein interaction is one of the major functional mechanisms of lncRNAs and has been extensively studied. In this study, we explored the role of CATED in increasing DHX36 protein levels. We first noticed that E3 ubiquitin ligases were also detected in CATED pulldown and DHX36 co‐IP, which led us to suspect that CATED regulates DHX36 ubiquitination. However, CATED overexpression did not affect DHX36 ubiquitination, indicating that the elevated levels of DHX36 during CATED overexpression might be mediated by other mechanisms. Furthermore, the detection of SUMO led us to provide evidence that CATED upregulates and stabilizes DHX36 levels via PIAS1‐mediated SUMOylation. SUMOylation is a key mechanism by which cells respond to stress and is frequently upregulated in cancers.^[^ ^23^, ^41^ ^]^ However, the understanding of lncRNAs in mediating SUMOylation of interacting proteins is limited.^[^ ^42^ ^]^ Therefore, our findings expand our knowledge of lncRNAs exerting critical post‐transcriptional roles along with co‐factors in modulating the SUMOylation of proteins. Additionally, our data demonstrated that CATED overexpression enhanced the DHX36‐PIAS1 interaction, suggesting CATED may function as a molecular scaffold to facilitate the binding of DHX36 and PIAS1. The precise binding for DHX36 and PIAS1 on CATED, including the different binding sites on CATED, would facilitate our understanding of CATED acting as a scaffold for its dynamic interactions of DHX36 and PIAS1. On the other hand, endeavors in exploring whether DHX36 interacts with PIAS1 directly or dependently on RNAs such as CATED require further investigation, and this will shed light on the functional mechanisms and the potential therapeutic use of CATED in the future.

DHX36 has emerged as a critical RBP that binds multiple RNAs.^[^ ^27^, ^43^ ^]^ CLIP‐seq analysis and RIP‐seq data in our study further demonstrated that DHX36 binds various RNAs, including CATED (Figures S5e and S9d, Supporting Information).210‐376 aa region in RecA1 domain of DHX36, is confirmed indispensable for DHX36 binding with CATED. RecA domain is known for its role in DNA binding,^[^ ^44^ ^]^ and it is also involved in RNA binding, particularly for DEAD‐box family proteins, including DHX36.^[^ ^45^ ^]^ Meanwhile, DHX36 has been reported to exhibit robust G4 binding and unwinding activity and promote mRNA translation by unwinding rG4 structures formed at the 5’ UTR.^[^ ^27^, ^46^ ^]^ For example, DHX36 specifically controls Gnai2 mRNA translation by unwinding its 5’ UTR rG4 structures during skeletal muscle development.^[^ ^27^ ^]^ DHX36 unwinds the rG4 structure at the Nkx2‐5 mRNA 5’ UTR, thereby enhancing its translation.^[^ ^46^ ^]^ Consistent with previous studies, we also identified that elevated DHX36 promotes RAP1A translation by unwinding its 5’ UTR rG4 structures, without affecting its mRNA level. Nevertheless, owing to the nature of DHX36 post‐transcriptional regulation, we cannot exclude the possibility that DHX6 may also regulate additional downstream targets in cisplatin resistance. Recent studies have shown that DHX36 induces RNA structural remodeling at its binding sites and across the entire mRNA, particularly in the 3' UTR, promoting YTHDF1 binding to m6A sites and subsequent RNA degradation.^[^ ^43^ ^]^ Given its role in enhancing mRNA decay via AU‐rich elements in the 3' UTR,^[^ ^19^ ^]^ it would be valuable to investigate whether DHX36 influences the RNA abundance of other key downstream targets in HGSOC chemoresistance.

Among the multiple mechanisms of drug resistance in cancers, dysregulation of cancer‐associated signaling pathways remains a hotspot. We identified the MAPK signaling pathway in both RNA‐seq of CATED overexpression and DHX36 RIP, which primarily plays a role in promoting cell proliferation and anti‐apoptotic effects. The MAPK pathway is located downstream of many growth factor receptors, including the Ras family member RAP1A, and the RAP1A/MAPK pathway has been reported to have an array of known functions in HGSOC and other cancers.^[^ ^47^ ^]^ The MAPK pathway may not be the sole pathway involved in the CATED‐DHX36 axis because platinum resistance is a multifaceted process. Our mechanistic elucidation of the CATED‐DHX36‐RAP1A‐MAPK axis could serve as a proof‐of‐concept for a comprehensive understanding of the lncRNA‐mediated networks in HGSOC chemotherapy. Additionally, our data demonstrated that ASO knockdown of CATED exhibited strong effects in restoring chemosensitivity in vivo, indicating that either the use of a CATED inhibitor or blocking the CATED‐DHX36‐RAP1A‐MAPK axis could be an efficient measure to alleviate chemoresistance in HGSOC. Potential toxicity evaluation and additional assessment for off‐target effects should be elucidated further before ASO‐CATED can be used in pre‐clinical and clinical treatment for platinum resistance of HGSOC.

## 4 Experimental Section

### Clinical Specimens

All fresh HGSOC tumor tissues were obtained from The First Affiliated Hospital of the University of Science and Technology of China (USTC) and were approved by the Human Research Ethics Committee (2021‐KY108). The inclusion criteria were as follows: 1) Patients diagnosed with HGSOC who underwent primary surgery at the Department of Obstetrics and Gynecology, First Affiliated Hospital of USTC; 2) Disease staged II–IV according to the 2014 FIGO classification; 3) Exclusion of patients who received radiotherapy or chemotherapy prior to surgery; 4) Exclusion of patients with insufficient initial clinical or pathological data; 5) Exclusion of patients with incomplete clinical follow‐up data, who were not included in survival analysis. Informed consent was obtained from all patients. After surgery, samples were rapidly frozen in liquid nitrogen for storage.

### Isolation of sEVs from Tumor Tissues and Cell‐Cultured Medium

The tumor or cell culture medium (serum‐free) was sequentially centrifuged (500 × g for 10 min, 2000 × g for 20 min, 16 000 × g for 45 min, and 100 000 × g for 90 min (repeated once)) at 4 °C. The sEVs were resuspended in Phosphate Buffer Solution (PBS), and the supernatant obtained was filtered through a 0.22 µm filter (Biosharp, BS‐PES‐22).

### Co‐Immunoprecipitation

Co‐IP assays were performed as previously described.^[^ ^8^ ^]^ Briefly, cells were processed in lysis buffer (50 mM Tris‐HCl, pH 8.0, 150 mM NaCl, 1% NP40, 1 mM DTT, and 1 × Protease Inhibitor Cocktail). For SUMOylation analysis, 20 mM N‐ethylmaleimide (NEM) was used to stabilize SUMO conjugates.^[^ ^48^ ^]^ The lysates were centrifuged at 12 000 × g at 4 °C for 15 min, and the supernatant was incubated with antibodies for 4 h at room temperature. The protein complexes were incubated with 100 µL Dynabeads Protein G (Invitrogen) for 2 h, which was pre‐blocked with 5% BSA at room temperature for 30 min in advance. Finally, the protein complexes bound to the beads were washed with lysis buffer and analyzed using western blotting. All antibodies are listed in Table S6, Supporting Information.

### Immunofluorescence Staining

SKOV3 and COV504 cells were fixed and permeabilized using the same methods as for FISH but without RNase inhibitors. After blocking with 5% BSA for 30 min at room temperature, samples were incubated with anti‐DHX36 antibody or anti‐PIAS1 antibody for 4 h at room temperature. Following three 5‐min washes with PBST buffer, samples were incubated with Goat anti‐rabbit secondary antibody Alexa Fluor 488 or Goat anti‐mouse secondary antibody Alexa Fluor 647 (both 1:100 dilution in 5% BSA) for 2 h at room temperature, protected from light. After an additional three 5‐min washes with PBST buffer, nuclei were stained with DAPI. The images were captured using a confocal microscope (Zeiss LSM980 with Airyscan). All antibodies are listed in Table S6, Supporting Information.

### Northern Blotting

Northern blotting was performed following established protocols. DIG‐labeled RNA probes targeting the antisense strand of CATED were generated using the DIG Northern Starter Kit (Roche, 12039672910) following the manufacturer's instructions. The primers used for probe synthesis are detailed in Table S7, Supporting Information.

### Fluorescence In Situ Hybridization

A Cy3‐labeled CATED probe FISH kit (GenePharma Technology, Shanghai, China) was used according to the manufacturer's guidelines. Briefly, cells were seeded onto coverslips in each well of a six‐well plate and cultured until they reached 50% confluence. Coverslips with cells were washed twice with ice‐cold PBS and fixed with cold fixation buffer (methanol: acetic acid = 3:1) for 10 min. After removing the fixation buffer, the coverslips were immersed in PBS containing 1% Triton X‐100 and 0.5% RNase inhibitors (Abclonal, RK21401) on ice for 20 min, and then washed three times with PBST (PBS with 0.4% Tween‐20), each for 5 min. The CATED probes were denatured at 75 °C for 10 min and then incubated with Cy3 at 37 °C for 30 min. The Cy3‐labeled CATED probe was incubated with the cells overnight at 37 °C for 16 h, and the cell coverslips were washed with 2 × SSC for 10 min. Finally, the nuclei were stained with DAPI. Images were captured using a confocal microscope (Zeiss LSM980 with Airyscan) and the corresponding analysis was conducted using ImageJ. The sequences of the RNA FISH probes are listed in Table S7, Supporting Information.

### Polysome Profiling Assay

The polysome profiling assay was performed following a previously described protocol.^[^ ^49^ ^]^ Cells were treated with 100 µg mL^−1^ cycloheximide for 30 min. The cell pellets were lysed in 300 µL lysis buffer (100 mM KCl, 5 mM MgCl_2_, 10% glycerol, 50 mM HEPES, 0.1% Triton X‐100, 1 mM DTT, 20 U mL^−1^ RNase inhibitor, 1 × PIC, 1 × PPIC, 100 µg mL^−1^ Cycloheximide) and centrifuged at 15 000 rpm for 10 min to remove the nuclei and membrane debris. A 50 µL aliquot of the lysis was saved as input, while the remaining 250 µL was loaded onto a sucrose gradient (10–50% sucrose (w/v) prepared using Gradient Master, 100 mM NaCl, 5 mM MgCl_2_, 10 mM Tris‐HCl pH 7.5, 1 mM DTT). The gradient was centrifuged in a SW41 rotor (Beckman at 38, 000 rpm) for 3 h in 4 °C. The polysome profiling profile curve was generated by optical scanning at 254 nm using a Gradient Profiler (BioComp), and the LMW and HMW fractions were collected for subsequent RNA analysis.

### The sEVs Uptake Assay

The sEVs were labeled using a PKH‐67 labeling kit (Umibio UR52303) according to the manufacturer's guidelines and then cocultured with SKOV3 and COV504 cells for 24 h. For in vitro sEVs assays, 1 µg sEVs was added to 2 × 10^5^ recipient cells. PKH‐67‐labeling sEVs were incubated with recipient cells and subsequently stained using FISH to detect the co‐localization of CATED and sEVs. Images were obtained using a confocal microscope (Zeiss LSM980 with Airyscan).

### RNA Pulldown and RNA Immunoprecipitation

RNA pulldown using 5’‐biotinylated antisense oligos and RIP was performed as previously described with minor modifications.^[^ ^49^ ^]^ SKOV3 and COV504 cells were UV cross‐linked (a total of 0.4 J cm^−2^). Cells were harvested in ice‐cold lysis buffer (50 mM Tris‐HCl, pH 8.0, 150 mM NaCl, 5 mM EDTA, 1% NP40, 0.1% SDS, 1 mM DTT, 1 × Protease Inhibitor Cocktail and 0.1 U µL^−1^ RNase inhibitor) for 15 min on ice. The lysate was then sonicated on ice for 5 min using a Sonics Vibra‐Cell (3 s on, 6 s off, 30% energy), and the supernatant was collected by centrifugation at 12 000 × g for 15 min. Biotinylated oligos (100 pmol for pulldown) or 2 µg of antibody (for RIP) were added to the supernatant. After overnight rotation at 4 °C, 50 µL of M‐280 Streptavidin Dynabeads (Invitrogen, 11206D, for RNA pulldown) or Protein G Dynabeads (Invitrogen, 10004D, for RIP), which were blocked with 500 ng µL^−1^ yeast total RNA and 5% BSA for 1 h at room temperature, were added. Following another 2 h of rotation at room temperature, the beads were washed once with lysis buffer and twice with high salt lysis buffer (50 mM Tris‐HCl, pH 8.0, 500 mM NaCl, 5 mM EDTA, 1% NP40, 0.1% SDS, 1 mM DTT, 1 × Protease Inhibitor Cocktail and 0.1 U µL^−1^ RNase inhibitor). The purified RNAs were analyzed by RT‐qPCR, and the purified proteins were analyzed by mass spectrometry after silver staining using the Protein Stains K kit (Sangon Biotech, C500021‐0010) or western blotting. The biotinylated oligos are listed in Table S7, Supporting Information.

### RIP‐Seq and Analysis

RIP‐seq was performed as previously described.^[^ ^49^ ^]^ RNA from anti‐DHX36 RIP was extracted using a TRIzol reagent according to the manufacturer's protocol. For RIP‐seq cDNA library preparation, purified RNAs were ligated to adapters, reverse‐transcribed, and PCR‐amplified for ≈25 cycles. The resulting libraries were subjected to high‐throughput sequencing using an Illumina Novoseq platform with a 150‐nt run length. Sequencing reads were trimmed for adapters using fastp and mapped to the human genome (hg38) using Bowtie2 with default parameters.^[^ ^50^, ^51^ ^]^ To visualize sequencing reads across the genome, bam files were converted to bigWig files using bedtools and displayed in IGV. Peaks were then called using MACS2 and annotated using bedtools.^[^ ^52^, ^53^ ^]^

### In Vivo Experiments

5‐week‐old female BALB/c nude mice were purchased from SPF (Beijing) Biotechnology Co., Ltd., and housed under SPF‐level conditions. Mice were injected intraperitoneally with 5 × 10^6^ cells (SKOV3‐EV/SKOV3‐CATED OE or SKOV3‐shCtrl/SKOV3‐shCATED) with luciferase to establish an intraperitoneal tumor model (n = 6 per group). Mice were treated with cisplatin (5 mg kg^−1^ every 3 days). For treatment involving sEV‐mediated transfer of CATED, 5 × 10^6^ WT SKOV3 cells with luciferase were injected intraperitoneally into 5‐week‐old female nude mice. These mice were also treated with cisplatin and 10 µg of the indicated sEVs from cells or patient tumor tissues every 3 days. In the treatment involving ERKi (ERK inhibitor) or ASO‐CATED, ERKi (SCH772984 from MCE, at a dose of 25 mg kg^−1^ per mouse) was administered via intraperitoneal injection. Gapmer ASO‐targeting CATED was delivered through tail vein injection at a dose of 10 nmol (100 µL, 100 mM ASO in PBS) per mouse, and the administration was performed every 3 days. Mice were intraperitoneally injected with d‐Luciferin (50 mg kg^−1^) and observed using living image software. The average luminescence signals were measured. The mice were sacrificed after 4 weeks. Animal experiments were approved by the Animal Care and Use Committee of the First Affiliated Hospital of USTC (2024‐N(A)‐0121).

### Statistical Analysis

Statistical analysis was performed using GraphPad Prism 9 software. Data are presented as the mean ± SD from at least three independent replicates. Two‐tailed Student's *t*‐test, log‐rank test and ANOVA test were used to calculate *p* values, as specified in the relevant figure legends. *p* values < 0.05 were considered statistically significant. The detailed statistical method and the sample sizes (n) for relevant experiments are described in the figure legends.

## Conflict of Interest

The authors declare no conflict of interest.

## Author Contributions

Y. L. Conceptualization, Data curation, Formal analysis, Investigation, Methodology, Validation, Visualization, Writing‐original draft, Writing‐review and editing. H. L. Conceptualization, Data curation, Formal analysis, Validation, Writing‐review and editing. C. Z. Data curation, Formal analysis, Investigation, Methodology, Writing‐review and editing. Y. Y. Formal analysis, Methodology, Visualization, Writing‐review and editing. Z. S. Conceptualization, Resources, Investigation, Writing‐review and editing. G. S. Conceptualization, Funding acquisition, Investigation, Writing‐original draft, Writing‐review and editing. L. C. Conceptualization, Formal Analysis, Funding acquisition, Methodology, Project administration, Supervision, Writing‐original draft, Writing‐review and editing. Y. Z. Conceptualization, Data curation, Formal Analysis, Funding acquisition, Investigation, Methodology, Project administration, Resources, Supervision, Writing‐original draft, Writing‐review and editing.

## Supporting information



Supporting Information

Supporting Information

Supporting Information

## Data Availability

All original data generated from Next Generation Sequencing (NGS) were deposited in the Gene Expression Omnibus (GEO) database (GSE282004). All study data and materials have been included in the article and/or supplementary data.
