# Supplementary material for: Tumor Small Extracellular Vesicle‐Transmitted LncRNA CATED Promotes Platinum‐Resistance in High‐Grade Serous Ovarian Cancer
Source: Adv Sci (Weinh). 2025 Jun 10;12(31):e05963. doi: 10.1002/advs.202505963 (PMC12376677; doi:10.1002/advs.202505963)
Supplement: Supplementary file 1 — Supporting Information [file ADVS-12-e05963-s002.docx]

**Supplemental Figure Legends:**

**Figure S1.** Characterization of the isolated small extracellular vesicles (sEVs) from tumors of HGSOC patients. a) A standardized workflow for isolating tumor-derived sEVs in Patients with HGSOC. b) Representative images of isolated sEVs from HGSOC tumor using transmission electron microscopy (TEM). Scale bar: 100 nm. S, sensitive; R, resistant; sEV, small extracellular vesicle. c) Size analysis of the isolated tumor-derived sEVs using Nanoparticle Tracking Analysis (NTA). d) Western blotting results demonstrating the presence of sEVs markers Alix, HSP70, TSG101, and CD9, with GM130 and Calnexin serving as negative controls.

**Figure S2.** Characterization of CATED. a) Schematic illustration of the genomic locus of CATED on the chromosome, including neighboring protein-coding genes. b) Analysis of the TCGA database shows CATED expression across various tumor types. c) Up: Schematic diagram of the 5’/3’ RACE assays. Low: the CATED transcript sequence was validated by agarose gel electrophoresis (left) and Sanger sequencing (right) of the 5’ RACE and 3’ RACE product in SKOV3 cells. d) Coding potential of CATED predicted by Coding Potential Calculator. e) Represented polysome profiling curve and the expression of CATED in either LMW or HMW. LMW, low molecular weight fraction; HMW, high molecular weight fraction. β-actin mRNA was a positive control and lncRNA MALAT1 was a negative control. f) GFP tag was inserted before thee stop codon of the ORFs predicted by ORFfinder in CATED and performed Western blot analysis, with GFP serving as a positive control. The data were from three repeats.

**Figure S3.** CATED promotes cisplatin resistance both *in vivo* and *in vitro*. a) Evaluation of CATED overexpression and knockdown efficiency in SKOV3 and COV504 cells. EV: empty vector; OE: overexpression; Ctrl: control. b, c) Cleaved PARP and cleaved-Caspase 3 protein levels quantified in CATED overexpression or knockdown cells. EV: empty vector; OE: overexpression; Ctrl: control. c-PARP, Cleaved PARP; c-Caspase 3, cleaved-caspase 3. The assays were conducted under conditions where cells were treated with cisplatin at the indicated concentrations for 36 hours. d) Representative images of intraperitoneal tumor-bearing nude mice injected with SKOV3 CATED OE cells with luciferase and treated with cisplatin at the specified time (n = 6 mice per group; one mouse shown in the main figure and the remaining five mice shown in the supplementary figure). e) Analysis of CATED levels in tumor tissues derived from the various groups of mice. f, g) immunohistochemical (IHC) analysis of the indicated markers in tumors excised from each group. The quantification of these markers across the different groups is illustrated in the bar figures. Scale bar = 20 μm. c-Caspase 3, cleaved-Caspase 3. h) Representative images of intraperitoneal tumor-bearing nude mice injected with SKOV3 CATED knockdown cells with luciferase and treated with cisplatin at the specified time (n = 6 mice per group; one mouse shown in the main figure and the remaining five mice shown in the supplementary figure). i) Analysis of CATED levels in tumor tissues derived from the various groups of mice. j, k) IHC analysis of the indicated markers in tumors excised from each group. The quantification of these markers across the different groups is illustrated in the bar figures. Scale bar = 20 μm. Dated are shown as mean ± SD. *P* values were determined with two-tailed Student’s *t*-test. **P < 0.05,* ****P < 0.001,* *****P < 0.0001*. All data were from at least three repeats.

**Figure S4.** sEV-mediated CATED overexpression enhanced HGSOC platinum resistance. a) Schematic representation illustrates the preparation of sEV-mediated CATED overexpression during the transfer from donor cells to recipient cells. PKH67 was employed as a fluorescent dye for labeling and visualizing sEVs. b) Representative transmission electron microscopy (TEM) images of sEVs isolated from the culture media of donor cells are presented, with a scale bar of 100 nm. c) CATED levels were examined in sEVs derived from donor cells of CATED overexpression and corresponding control cells. sEV-CATED, sEVs from CATED overexpression cells; sEV-EV, sEVs from corresponding empty control cells. d) Representative images of the xenografts intraperitoneally established by SKOV3 WT cells with luciferase and injected with sEVs from CATED overexpression and control cell cultures. The mice were administered with indicated sEVs and cisplatin every three days. The control group was only injected with cisplatin (n = 6 mice per group; one mouse shown in the main figure and the remaining five mice shown in the supplementary figure). e) Analysis of CATED levels in tumor tissues derived from the various groups of mice using sEVs from donor cells. CATED-sEV: sEV-mediated CATED overexpression; EV-sEV: sEV-mediated corresponding empty control. f) CATED expression levels were analyzed in the sEVs derived from tumors of platinum-resistant and -sensitive patients for *in vivo* experiments. g) Representative images of the xenografts established intraperitoneally using SKOV3 WT cells with luciferase injected with sEVs derived from patients’ tumor tissues. The mice received the indicated sEVs and cisplatin every three days (n = 6 mice per group; one mouse shown in the main figure, and the remaining five mice shown in the supplementary figure). h) Analysis of CATED levels in tumor tissues derived from the various groups of mice using tumor-derived sEVs. Dated are shown as mean ± SD for c, e, f, h. *P* values were determined with two-tailed Student’s *t*-test. **P < 0.05,* ***P < 0.01,* ****P < 0.001,* ns. Not significant. All data were from at least three repeats.

**Figure S5.** CATED interacts with DHX36. a) AGO2 RIP assay with SKOV3 cells followed by the detection of CATED in the RIP materials. b) The identified peptide of DHX36 from MS assay. c) Western blot analysis of AGO2 identified that RNA pulldown of CATED co-pulls down in SKOV3 cell. Scr: represents the negative control using a biotin-labeled oligo with scrambled sequences; Oligo: represents the biotin-labeled oligo with antisense sequences to the CATED. d) Universal DHX36 binding motif on RNAs identified by PAR-seq analysis from HEK293 cells (GSE105171). e) Three DHX36 binding sites on CATED in HEK293 cells from the PAR-CLIP (GSE105171). Mutation of the DHX36 binding motifs in the respective three binding sites on CATED, as identified by PAR-CLIP data analysis, with the mutant sequences shown schematically. f) Validation of DHX36 overexpression efficiency in SKOV3 and COV504 cells. Dated are shown as mean ± SD. *P* values were determined with two-tailed Student’s *t*-test. ns. Not significant. All data were from three repeats.

**Figure S6.** CATED increases DHX36 level. a) Validation of DHX36 knockdown efficiency in SKOV3 and COV504 cells. b, c) RT-qPCR examined CATED levels in DHX36 overexpression and knockdown cells. NC: normal control. d, e) RT-qPCR examined DHX36 mRNA levels in CATED overexpression and knockdown cells. f) DHX36 protein levels were assessed by Western blot in cells with CATED overexpression, either alone or in conjunction with DHX36 knockdown. g) Cell viability was evaluated using the CCK8 assay following CATED overexpression alone or with DHX36 knockdown in SKOV3 and COV504 cells. h) TUNEL staining was assessed after CATED overexpression, either alone or in conjunction with DHX36 knockdown, in SKOV3 and COV504 cells. Cells were treated with cisplatin at indicated concentrations for 36 hours. Scale bar: 50 μm. Dated are shown as mean ± SD. *P* values were determined with two-tailed Student’s *t*-test. **P < 0.05,* ***P < 0.01,* ****P < 0.001,* *****P < 0.0001.* ns. Not significant. All data were from three repeats.

**Figure S7.** CATED promotes DHX36 SUMOylation. a) Protein levels were examined after incubation with MG132 (100 μM, 4 h, APExBIO, A2585), with PTBP1 and AUF1 used as positive controls. b) Co-IP assay using anti-DHX36 was performed to assess the conjunction of ubiquitination on DHX36 in cells after CATED overexpression. c) The identified peptides of SUMO2, SAE1, and UBA2 from MS assay. d) SUMOylaiton sites prediction on DHX36 using GPS-SUMO. e, f) Sequence of the DHX36^K105^ and DHX36^K139^ wild type and corresponding mutations. g) The domain structure of DHX36 and its amino acid sequences at positions 105 and 139. Data were from three repeats.

**Figure S8.** CATED serves as a scaffold in the interaction between PIAS1 and DHX36. a) The identified peptides of PIAS1 from MS assay. b) AlphaFold 3-predicted result that the specific binding sites (E747、R772 and D779) on DHX36 interact with PIAS1. The yellow dashed line in the figure represents an ionic bond. c, d) mRNA levels of SAE1, UBA2, UBC9, PIAS1, and SUMO2 in the CATED overexpression cells were measured. e) Protein levels of SAE1, UBA2, UBC9, PIAS1, and SUMO2 were analyzed in the CATED overexpression cells. f, g) RIP with an antibody against PIAS1 in the SKOV3 and COV504 cells pull down CATED. Western blots showing efficient pulldown of PIAS1. RT-qPCR analyses show the enrichment of CATED. h) Validation of PIAS1 knockdown efficiency in SKOV3 and COV504 cells. Dated are shown as mean ± SD for c, d, f, g. All data were from three repeats.

**Figure S9.** CATED increases RAP1A protein level via DHX36. a) Workflow of RIP-seq assay. b) The classification of DHX36-associated RNAs was shown. c) The distribution of overlapping sites across different mRNA regions. d) DHX36 binding signals on CATED in SKOV3 cells from our RIP-seq data. e) Western blot results showing efficient pulldown of DHX36 from RIP in SKOV3 and COV504 cells using an anti-DHX36 antibody. f) DHX36 and RAP1A mRNA levels were analyzed by RT-qPCR in cells with either CATED or DHX36 overexpression. g) RAP1A mRNA levels were examined by qPCR in CATED overexpression alone or with DHX36 knockdown. h) RAP1A protein levels were assessed by Western blot in cells with CATED overexpression, either alone or in conjunction with RAP1A knockdown. i, j) Cell viability was evaluated using the CCK8 assay following CATED overexpression alone or with RAP1A knockdown in SKOV3 and COV504 cells. k, l) TUNEL staining was assessed after CATED overexpression, either alone or in conjunction with RAP1A knockdown, in SKOV3 and COV504 cells. Cells were treated with cisplatin at indicated concentrations for 36 hours. Scale bar: 50 μm. Dated are shown as mean ± SD. *P* values were determined with two-tailed Student’s *t*-test. **P < 0.05,* ***P < 0.01,* ****P < 0.001,* *****P < 0.0001,* ns. Not significant. All data were from three repeats.

**Figure S10.** DHX36 increase RAP1A protein level via unwinding rG4 structure. a) Represented polysome profiling curve. LMW, low molecular weight fraction; HMW, high molecular weight fraction. b) Potential RNA G quadruplexes structures in the 5’ UTR of RAP1A predicted in G4Hunter (https://bioinformatics.ibp.cz). c) SKOV3 and COV504 cells were treated with 2.5 or 5 μM cPDS, and RAP1A mRNA expression was measured by RT-qPCR. d) The sequence of the wild type or mutant RAP1A mRNA 5’ UTR is displayed. Mutated Gs are highlighted in red. e) Schematic diagram of the 5’ UTR of RAP1A mRNA is shown as the 5’ UTR of firefly luciferase mRNA. f) Schematic diagram of the 5’ UTR of RAP1A and its mutant sequence, each constructed into plasmid with CDS of RAP1A. The diagram shows the design of specific primers for qPCR. Dated are shown as mean ± SD. *P* values were determined with two-tailed Student’s *t*-test. **P < 0.05,* ***P < 0.01,* ****P < 0.001*. The data were from three repeats.

**Figure S11.** CATED activates the MAPK pathway both *in vitro* and *in vivo*. a) GO analysis of upregulated and downregulated genes in CATED overexpression compared to the control. b) Representative images of xenografts established via intraperitoneal injection using SKOV3 WT cells with luciferase. The sEVs derived from donor cells were injected solely or with sEVs accompanied by simultaneous injection of ERKi. (n = 6 mice per group; one mouse shown in the main figure, and the remaining five mice shown in the supplementary figure). c-e) Analysis of CATED, DHX36, and RAP1A levels in tumor tissues derived from the various groups of mice using ERKi. ERKi: ERK inhibitors. Dated are shown as mean ± SD. *P* values were determined with two-tailed Student’s *t*-test. ****P < 0.001,* ns. Not significant. All data were from at least three repeats.

**Figure S12.** ASO-targeting CATED is proposed as a potential therapy. a, b) RT-qPCR analysis of other multiple well-characterized lncRNAs expression in CATED knockdown cells after ASO-mediated knockdown of CATED. c, d) DHX36 protein stability was assessed after CATED knockdown mediated by ASO-CATED in SKOV3 and COV504 cells. CHX, Cycloheximide. e) RT-qPCR detection of CATED levels in tumors following ASO-CATED-1 administration in a mouse intraperitoneal xenograft model. f) Representative images were conducted regarding the xenografts, which were established through intraperitoneal injection of SKOV3 WT cells with luciferase. The SKOV3 WT cells were subjected to either sEVs solely obtained from donor cells or sEVs in combination with simultaneous injection of ASO-CATED (n = 6 mice per group; one mouse shown in the main figure, and the remaining five mice shown in the supplementary figure). g-i) Analysis of CATED, DHX36, and RAP1A levels in tumor tissues derived from the various groups of mice treated with ASO targeting CATED. j) Analysis of other multiple well-characterized lncRNAs levels in tumor tissues derived from the various groups of mice treated with ASO targeting CATED. Dated are shown as mean ± SD. *P* values were determined with two-tailed Student’s *t*-test or ANOVA test. **P < 0.05,* ***P < 0.01,* ****P < 0.001*. All data were from at least three repeats.
