# Supplementary material for: Tumor Small Extracellular Vesicle‐Transmitted LncRNA CATED Promotes Platinum‐Resistance in High‐Grade Serous Ovarian Cancer
Source: Adv Sci (Weinh). 2025 Jun 10;12(31):e05963. doi: 10.1002/advs.202505963 (PMC12376677; doi:10.1002/advs.202505963)
Supplement: Supplementary file 2 — Supporting Information [file ADVS-12-e05963-s001.docx]

**Supporting Information**

**Tumor Small Extracellular Vesicle-Transmitted LncRNA CATED Promotes Platinum-Resistance in High-Grade Serous Ovarian Cancer**

*Yi Liu, Hanyuan Liu, Chenchen Zhu, Yan Yang, Zhen Shen, Ge Shan^*^, Liang Chen*, Ying Zhou^*^*

Y. Liu, H. Liu, C. Zhu, Z. Shen, Y. Zhou

Department of Obstetrics and Gynecology, Core Facility Center, The First Affiliated Hospital of USTC, Division of Life Sciences and Medicine, University of Science and Technology of China, Hefei, 230001, Anhui, China

E-mail: caddiezy@ustc.edu.cn

Y. Yang

Hefei National Laboratory for Physical Sciences at Microscale, School of Basic Medical Sciences, Division of Life Sciences and Medicine, University of Science and Technology of China, Hefei, 230027, China

G. Shan

Department of Obstetrics and Gynecology, The First Affiliated Hospital of USTC, The RNA Institute, School of Basic Medical Sciences, Division of Life Sciences and Medicine, University of Science and Technology of China, Hefei 230027, China

E-mail: shange@ustc.edu.cn

L. Chen

Department of Cardiology, The First Affiliated Hospital of USTC, The RNA Institute, Division of Life Sciences and Medicine, University of Science and Technology of China, Hefei, 230027, China

E-mail: anqingcl@ustc.edu.cn

**Supplemental Table 1. Clinical information of patients used for tumor small extracellular vesicles (sEVs) RNA-seq in this study.**

| Patient | FIGO (2014) | Age | Pathology | Platinum sensitivity |
| --- | --- | --- | --- | --- |
| SE1 | IIIC | 49 | HGSOC | Yes |
| SE2 | IVB | 51 | HGSOC | Yes |
| SE3 | IVA | 57 | HGSOC | Yes |
| SE4 | IIIC | 56 | HGSOC | Yes |
| RE1 | IIIC | 50 | HGSOC | No |
| RE2 | IIIC | 51 | HGSOC | No |
| RE3 | IIIC | 55 | HGSOC | No |

**Supplemental Table 2. The complete transcript of CATED was identified by RACE followed by Sanger sequence**

| 1 | GCTGCTGTAGCCCCTACGTG | GTGAGTAGATCGTCTCCACA | GAAATCAGCGGAGGCTTCCA | CAGTCCCTGGTTGGAATGGA | ACCGGCGGTGAGGATGAGAG |
| --- | --- | --- | --- | --- | --- |
| 101 | GCGCTGAAGACCAGAAGACC | CAGGGCGTGAACGGGGCGTG | TCATCCCTGGAGTTTCAGTA | GAATTTCAGGCCTGTCTGCT | TGAGCAGCAGAGGAGGAGTT |
| 201 | TCTGTGTGCCTGTGAGAGAC | TGTGTGTGTGTGTGTGTGTG | TGTGTGTGTGTGAGATGAAT | GAACAGGGTTGTGTACGTGT | GTGTGCGTTAGACAAATGAA |
| 301 | CAGTGTTGTGTACCTGTGTG | AATTGTCACTGTGACTCCAG | GCTCTAGGCTCTGCAGATTC | CTCAGCCCTATCGCCTCACA | GACACTTGGGATGTGCAGCA |
| 401 | GGTAAAAGAGAGAAGCCAGA | TTCATGGAGGCGTGAAGGAT | CCCAGCAAGAAAACACAGAC | TTGATCTTGAGACGAGATTT | TTGAAATTGGTTGCGGCCTC |
| 501 | AAGGCAGGCTCTGAGAAGAC | TCGATAAAGAGTTCTCAGGA | ATAGGCATCCCTCCTGGATA | TTTCCCCCTTGGAAGCGCCG | AGGAGCCAGCTCTATGTTTG |
| 601 | TAACCATAGCATTTCTAAAG | CTCCAGCCCCAGGAACCTGC | TGTGAATTAGAGACAGATTC | CACCCTAGGCATCACTGATC | TTGGCCTCTCTTGTATTGGG |
| 701 | AAGGTCAACACTGTTCCAGT | TTCTCTCCTACTCCCATCTG | GTTGTGCTCGGTGAAGTAGG | TGAGCAGTGCTACCCTTCTG | TTAAAAAAAAAAAAAAAAAA |
| 801 | AAGATCCTGAAAGAGGAAAG | AATTTTTGCTACACATCTGA | AAGCTAAAGTCCAGTTGAAC | TCCAGAGGAGAACACAGCCC | AGGTAACCCCTCGTTTGCAG |
| 901 | CCTGTGAGACCAGCCTGAGG | ACAGCACCCAGCTAAACTCC | AGGTCCATGGAAACTGTGAG | ATTTTATATATTTATGTTGT | TTTAAGCTAAATTCGTGGTA |
| 1001 | ATTGGTTATGGTAGCAACTG | AAAATTAATGCAGTAAGAAA | GTCTAAGGAGATCGGAAGAG | ACTAAAGAGAAATAACAGCT | AAATGCAACGTAGATTAATG |
| 1101 | TGTGGGATCCTGGAAAAGAA | AAAGGACATTAATGGGAAAA | CATATTTGAATAAGATGTGC | AAATTTTTCGTATTGTACCA | ATGTTAATTTCCTGGTTTTG |
| 1201 | ATAATTATCCTTTGGTTATG | TAAGATAAACGCTAGAATGA | AGGGTATATGAGAACCCTCT | GTACTACTCTTGCAACTTTT | CTCTAAGTCTAAAATAAGTG |
| 1301 | GAAATTAAAAGTTAAAACAA | AAAAAAAGGAAAATTTTATC | TATCCCAAGACCACAAAGTT | ATTTTTCTGTTTCTTCCTCC | AGAATCTTTATTGTTTGTTT |
| 1401 | TCACACTTAGACCTATGATC | CATCTTGAATTTATATCTGT | GTATGTAGTGGGATGAAGTT | CAAAATCATTTTTTTCATAC | AGATATCCAAGTGCACTGGC |
| 1501 | ACCGTTTGTTAAAAACACTG | GCCTTCTCTTAAGCTGCAGT | CAGACTTTTACTGTAAAGCA | TGGGCCCATATATGTGCAGG | AGTCTTTCTACTTTTCCGTT |
| 1601 | GGTCCTCTTTGTCAATCTTT | GTGCCAGCACCTCCATTTTA | ATTCCTGTAGCTTTATATTA | TATCTTGATAATCTGGTAAT | ATAAGTTGAAGAACAACTTT |
| 1701 | GTTCTTCAAAATCACTTTGT | CTATTCCAGGTTCTTTGCAT | TGCTCTATAATTTTAGAATG | AGTTAATTAATTTTCTCAAA | ACAAACAAAACGCCCTGCTG |
| 1801 | ACATTTTGTTGGCAGTACAT | TGAATCAGTAGATAAATTTA | GAGAGAATTATCTCAAAATT | GAGTCTTACAGTTCATGAAC | ATGGAATACATTTCCATTTA |
| 1901 | TTGAATTCATCTTTAATTTC | TTTCCTTAAGGTTTTGTTGT | TTCCTGTCTAAAGGTCTTGC | ACATCTGTTAAATTTATTCC | TGTTTTACATTTTTTATGTT |
| 2001 | TTTACAAACTGTTAAGGTTT | TAAAAATTTTATTTTTCAGT | AGCTTTGCTTTAATATAATT | GTTTTATACTTTTACCCTTT | ATTCAGCAATAAATTATTTA |
| 2101 | ATTTAAAGCTTGTGGAATTA | AAAAATATTTTTATGTACAT | AATCATGTAATCTACACTAT | CTGCAAATAAAGATCATTTT | ACTTCCTTCCA |

**Supplemental Table 3. Clinical information of patients for validation of tissue samples and sEVs in this study.**

| Patient | Age | FIGO (2014) | Pathology | Platinum sensitivity | recurrence | survival time |
| --- | --- | --- | --- | --- | --- | --- |
| 1 | 52 | IV | HGSOC | No | Yes | 8 |
| 2 | 77 | IIIC | HGSOC | No | Yes | 12 |
| 3 | 58 | IVB | HGSOC | Yes | No | 9 |
| 4 | 69 | IIIC | HGSOC | Yes | No | 14 |
| 5 | 72 | IIIC | HGSOC | No | Yes | 4 |
| 6 | 54 | IIIC | HGSOC | Yes | No | 13 |
| 7 | 58 | IIIC | HGSOC | Yes | No | 21 |
| 8 | 54 | IIB | HGSOC | Yes | No | 23 |
| 9 | 53 | IIIC | HGSOC | Yes | No | 13 |
| 10 | 55 | IVB | HGSOC | Yes | No | 28 |
| 11 | 86 | IIIC | HGSOC | Yes | Yes | 29 |
| 12 | 64 | IVB | HGSOC | No | Yes | 9 |
| 13 | 55 | IIIC | HGSOC | Yes | No | 13 |
| 14 | 56 | IIIC | HGSOC | Yes | No | 12 |
| 15 | 52 | IVB | HGSOC | Yes | No | 10 |
| 16 | 45 | IIIC | HGSOC | Yes | Yes | 13 |
| 17 | 55 | IVB | HGSOC | Yes | Yes | 31 |
| 18 | 54 | IIIC | HGSOC | Yes | Yes | 10 |
| 19 | 54 | IIIC | HGSOC | No | Yes | 10 |
| 20 | 63 | IIIC | HGSOC | Yes | No | 24 |
| 21 | 38 | IIIC | HGSOC | Yes | No | 24 |
| 22 | 53 | IIIC | HGSOC | No | Yes | 9 |
| 23 | 56 | IIIC | HGSOC | Yes | Yes | 12 |
| 24 | 54 | IIIC | HGSOC | No | Yes | 8 |
| 25 | 56 | IIIC | HGSOC | Yes | Yes | 9 |
| 26 | 39 | IIIC | HGSOC | No | Yes | 12 |
| 27 | 50 | IIIC | HGSOC | No | Yes | 41 |
| 28 | 87 | IIIC | HGSOC | No | Yes | 7 |
| 29 | 55 | IIIC | HGSOC | Yes | No | 16 |
| 30 | 50 | IIIC | HGSOC | No | Yes | 12 |
| 31 | 53 | IIIC | HGSOC | No | Yes | 11 |
| 32 | 53 | IIIC | HGSOC | Yes | No | 12 |
| 33 | 53 | IIIC | HGSOC | Yes | No | 13 |
| 34 | 48 | IIIA2 | HGSOC | Yes | No | 42 |
| 35 | 51 | IVB | HGSOC | No | Yes | 10 |
| 36 | 55 | IVB | HGSOC | Yes | No | 13 |
| 37 | 45 | IV | HGSOC | No | Yes | 9 |
| 38 | 60 | IIIC | HGSOC | Yes | No | 13 |
| 39 | 59 | IIIC | HGSOC | Yes | No | 12 |
| 40 | 55 | IIIC | HGSOC | Yes | No | 27 |
| 41 | 52 | IIIC | HGSOC | No | Yes | 8 |
| 42 | 55 | IIIC | HGSOC | Yes | No | 13 |
| 43 | 64 | IIIC | HGSOC | Yes | No | 10 |
| 44 | 81 | IIB | HGSOC | Yes | No | 14 |
| 45 | 68 | IIIC | HGSOC | Yes | No | 13 |
| 46 | 51 | IIIC | HGSOC | Yes | Yes | 16 |

**Supplemental Table 4. Top 5 enriched proteins detected in mass spectrometry from CATED pulldown.**

| Name | Peptides | Coverage | MW (kDa) |
| --- | --- | --- | --- |
| DHX36 | 4 | 4.76% | 114.8 |
| ADAMTS2 | 1 | 0.66% | 134.8 |
| DSG1 | 1 | 0.95% | 113.7 |
| MPRIP | 1 | 0.59% | 116.5 |
| PIWIL3 | 1 | 0.79% | 101.1 |

**Supplemental Table 5. SUMOylation-related proteins detected in mass spectrometry of DHX36 co-IP.**

| Name | Peptides | Coverage | MW (kDa) |
| --- | --- | --- | --- |
| SUMO2 | 2 | 23.20% | 10.871 |
| PIAS1 | 15 | 21% | 71.835 |
| SAE1 | 1 | 5.80% | 38.449 |
| UBA2 | 1 | 1.90% | 71.223 |

**Supplemental Table 6. Antibodies used in this study.**

| Antigens | Catalog | Manufacturer | Application |
| --- | --- | --- | --- |
| Alix | 12422-1-AP | Proteintech | WB |
| HSP70 | 10995-1-AP | Proteintech | WB |
| CD9 | 60232-1-AP | Proteintech | WB |
| TSG101 | ab125011 | Abcam | WB |
| Calnexin | 10094-1-AP | Proteintech | WB |
| GM130 | 11308-1-AP | Proteintech | WB |
| cleaved PARP | 9541S | Cell Signaling Technology | WB |
| cleaved caspase 3 | 9661T | Cell Signaling Technology | WB |
| cleaved caspase 3 | 25128-1-AP | Proteintech | IHC |
| Tubulin | 10094-1-AP | Proteintech | WB |
| DHX36 | 13159-1-AP | Proteintech | WB, IP, IHC |
| GFP | HT801 | TRANS | WB |
| DHX36 | PA5-57259 | Thermo | IF |
| FLAG | F1804 | Sigma | WB, IP |
| SUMO-2/3 | 4971T | Cell Signaling Technology | WB |
| His | M20001 | Abmart | WB, IP |
| PIAS1 | 23395-1-AP | Proteintech | WB, IP |
| PIAS1 | sc-365127 | SANTA | IF |
| HA | HT301 | TRANS | WB |
| RAP1A | 68125-1-Ig | Proteintech | WB |
| p-MEK1/2 | 9154S | Cell Signaling Technology | WB |
| p-MEK1/2 | 2338S | Cell Signaling Technology | IHC |
| MEK1/2 | 11049-1-AP | Proteintech | WB, IHC |
| p-ERK1/2 | 28733-1-AP | Proteintech | WB, IHC |
| ERK1/2 | 11257-1-AP | Proteintech | WB, IHC |
| GAPDH | 60004-1-Ig | Proteintech | WB |
| anti-AGO2 | SAB4200085 | Sigma | WB, IP |
| SAE1 | 10229-1-AP | Proteintech | WB |
| UBA2 | 15347-1-AP | Proteintech | WB |
| UBC9 | 10070-1-AP | Proteintech | WB |
| ubiquitin | 10201-2-AP | Proteintech | WB |
| Ki-67 | 27309-1-AP | Proteintech | IHC |
| Goat anti-mouse IgG secondary antibody HRP conjugated | L3032 | Signalway Antibody | WB |
| Goat anti-rabbit IgG secondary antibody HRP conjugated | L3012 | Signalway Antibody | WB |
| Normal rabbit IgG | 2729 | Cell Signaling Technology | IP |
| Normal mouse IgG | B900620 | Proteintech | IP |
| Goat anti-rabbit IgG H&L (Alexa Fluor® 488 | ab150077 | Abcam | IF |
| 647-conjugated goat anti-mouse IgG (H+L) | AS059 | Abclonal | IF |

**Supplemental Table 7. Sequences of primers and probes used in this study.**

| Application | Name | Sequences | |
| --- | --- | --- | --- |
| RNA FISH | CATED FISH probe | 5’-3’ | CTACTGATTCAATGTACTGCCAACA |
| RNA pulldown | CATED Oligo | 5’-3’ | TGCAAAGAACCTGGAATAGACAAAGTGATTTTGAAGAACAAAGTTGTTCT |
|  | Scramble | 5’-3’ | TTCTCCGAACGTGTCACGTTCGAACGTGTC |
| RACE | 5’ RACE Primer | | GATTACGCCAAGCTTGCTGGGTGCTGTCCTCAGGCTGGTCTCA |
|  | 3’ RACE Primer | | GATTACGCCAAGCTTTGAGACCAGCCTGAGGACAGCACCCAGC |
| Northern Blot | Antisense probe | 5’-3’ | TACCTGGGCTGTGTTCTCCTCT |
|  | sense probe | 5’-3’ | GAAGGTCAACACTGTTCCA |
| qRT-PCR | β-actin | Forward (5’-3’) | CTTCGCGGGCGACGAT |
|  |  | Reverse (5’-3’) | CCACATAGGAATCCTTCTGACC |
|  | CATED | Forward (5’-3’) | ACAGATTCCACCCTAGGCAT |
|  |  | Reverse (5’-3’) | GCACTGCTCACCTACTTCAC |
|  | U1 | Forward (5’-3’) | GATACCATGATCACGAAGGTG |
|  |  | Reverse (5’-3’) | CTACCACAAATTATGCAGTCG |
|  | lncRNA MALAT1 | Forward (5’-3’) | GGACTTGAGCTGAGGTGCTT |
|  |  | Reverse (5’-3’) | GCTTCACCACCACATCCGTA |
|  | lncRNA H19 | Forward (5’-3’) | TACAACCACTGCACTACCTG |
|  |  | Reverse (5’-3’) | TGGAATGCTTGAAGGCTGCT |
|  | lncRNA PANDAR | Forward (5’-3’) | TGCACACATTTAACCCGAAG |
|  |  | Reverse (5’-3’) | CCCCAAAGCTACATCTATGACA |
|  | lncRNA UCA1 | Forward (5’-3’) | CAGATCCTTGCCCATGGTGT |
|  |  | Reverse (5’-3’) | CTGAGGCTGGCAAAGAGTGA |
|  | lncRNA TUG1 | Forward (5’-3’) | AGGTAGAACCTCTATGCATTTTGTG |
|  |  | Reverse (5’-3’) | ACTCTTGCTTCACTACTTCATCCAG |
|  | lncRNA PVT1 | Forward (5’-3’) | GGAAAGGATGTTGGCGGTC |
|  |  | Reverse (5’-3’) | CAGAAAATACTTGAACGAAGCTCC |
|  | DHX36 | Forward (5’-3’) | GGTCGAGCTGGAAGAGTTCA |
|  |  | Reverse (5’-3’) | TGCCTCATTTGATGGTGGGT |
|  | RAP1A | Forward (5’-3’) | GGGAAGTCTGCTCTGACAGT |
|  |  | Reverse (5’-3’) | GCAAAACCTTGGCCGTTCTT |
|  | RAP1A (polysome) | mutForward (5’-3’) | ATAGAGTAAGCGCCTGACCG |
|  |  | mutReverse (5’-3’) | actgtcagagcagacttccc |
|  |  | wtForward (5’-3’) | TGGAGGAGGTGGAGGAGGC |
|  |  | wtReverse (5’-3’) | actgtcagagcagacttccc |
|  | SAE1 | Forward (5’-3’) | GGTGGCTGTCTTTGTTCCAG |
|  |  | Reverse (5’-3’) | AGAAGGTGACAAGAGGCTCC |
|  | UBA2 | Forward (5’-3’) | GCTGCCCGAAACCATGTTAA |
|  |  | Reverse (5’-3’) | AGGAAAGGTTCTCTGGGTCG |
|  | UBC9 | Forward (5’-3’) | AATTCTTCGTCCTGAGGCCA |
|  |  | Reverse (5’-3’) | GCTTCCCATCTCTGTCCACT |
|  | PIAS1 | Forward (5’-3’) | GACAGTGCGGAACTAAAGCAAA |
|  |  | Reverse (5’-3’) | TCTCGAAAGCGCTGACTGTT |
|  | SUMO2 | Forward (5’-3’) | GACGAAAAGCCCAAGGAAGG |
|  |  | Reverse (5’-3’) | CATTGATTGGTTGCCCGTCA |

**Supplemental Table 8. Sequences of shRNA, siRNA, and ASO used in this study.**

| Name | Sequence | |
| --- | --- | --- |
| shCATED-1 | sense (5’-3’) | GGTCAACACTGTTCCAGTTTCTTCAAGAGAGAAACTGGAACAGTGTTGACC |
|  | anti-sense (5’-3’) | GGTCAACACTGTTCCAGTTTCTCTCTTGAAGAAACTGGAACAGTGTTGACC |
| shCATED-2 | sense (5’-3’) | GGTTGTGCTCGGTGAAGTATTCAAGAGATACTTCACCGAGCACAACC |
|  | anti-sense (5’-3’) | GGTTGTGCTCGGTGAAGTATCTCTTGAATACTTCACCGAGCACAACC |
| siDHX36-1 | sense (5’-3’) | GUGGGUUAGUAAAGCUAAUTT |
|  | anti-sense (5’-3’) | AUUAGCUUUACUAACCCACTT |
| siDHX36-2 | sense (5’-3’) | CAGGGUUCUAUCUUAUACUTT |
|  | anti-sense (5’-3’) | AGUAUAAGAUAGAACCCUGTT |
| siRAP1A-1 | sense (5’-3’) | GGGCCAGAAUUUAGCAAGATT |
|  | anti-sense (5’-3’) | UCUUGCUAAAUUCUGGCCCTT |
| siRAP1A-2 | sense (5’-3’) | GACCUGGUCAGACAGAUAATT |
|  | anti-sense (5’-3’) | UUAUCUGUCUGACCAGGUCTT |
| siPIAS1-1 | sense (5’-3’) | CUCCAUAUGAACACCUUAUTT |
|  | anti-sense (5’-3’) | AUAAGGUGUUCAUAUGGAGTT |
| siPIAS1-2 | sense (5’-3’) | GACACAAGCUACAUUAAUATT |
|  | anti-sense (5’-3’) | UAUUAAUGUAGCUUGUGUCTT |
| ASO-CATED-1 | 5’-3’ | CUACUTCACCGAGCACAACC |
| ASO-CATED-2 | 5’-3 | UUUCAGTTGCTACCAUAACC |

**Supplemental Methods**

*Transmission Electron Microscopy (TEM):* The small extracellular vesicles (sEVs) were subjected to TEM analysis. 2.5 μL of sEVs suspended in PBS were adhered to a glow-discharged copper grid and stained with a 2% uranyl acetate solution for 90 seconds. The stained sEVs were subsequently observed using a TEM (FEI, the United States).

*Nanoparticle Tracking Analysis (NTA):* The sEVs particle size and concentration were measured using NTA with ZetaView PMX 120 (Particle Metrix, Meerbusch, Germany) and corresponding software ZetaView 8.06.01 SP7. The sEVs samples were diluted with 1× PBS buffer to measure particle size and concentration. NTA measurement was recorded and analyzed at 11 positions. The ZetaView system was calibrated using 100 nm polystyrene particles. The temperature was maintained around 23 °C and 30 °C.

*Cell Lines and Cell Culture:* The ovarian cancer cell lines SKOV3 and COV504 were obtained from American Type Cancer Culture and CELLCOOK (Guangzhou, China), respectively. Both the SKOV3 and COV504 cell lines were cultured under standard conditions in DMEM supplemented with 10% FBS (CLARK, FB25015) and 1% penicillin/streptomycin (Beyotime, C0222) at 37 °C under 5% CO_2_. All the cell lines were routinely checked for mycoplasma contamination using a PCR-based method and DAPI staining to confirm their purity.

*Library Preparation for RNA-Seq:* Total RNA was isolated from sEVs (7 samples) and cells (4 samples) using TRIzol reagent, with RNA quality verified by Bioanalyzer 2100. After Illumina 150-bp paired-end sequencing, raw reads were processed by removing adapters, poly-N, and low-quality sequences, followed by Q20/Q30 and GC content calculation. Clean reads were aligned to GRCh38 using HISAT2 (v2.2.1) with ENSG IDs converted to gene symbols. Differential gene expression analysis was performed using thresholds of p<0.05 and |log2FC|≥1, with results visualized by volcano plots in R.

*Western Blotting:* For western blotting, samples were separated using SDS-PAGE gels and transferred to PVDF membranes (Millipore). The membranes were then processed following the Enhanced Chemiluminescent Western blotting protocol (NCM Biotech, P10100). Images were taken with the ImageQuant LAS4000 Biomolecular Imager (GE Healthcare) and Grayscale analysis of the Western blot images was analyzed by ImageJ. Necessary global adjustments to brightness/contrast were uniformly applied to images using ImageJ for optimal visualization. For sEVs validation, the protein markers (Alix, HSP70, TSG101, CD9) were detected. All antibodies are provided in Supplementary Table S6.

*Nuclear/Cytosolic Fractionation:* Cellular fractionation was performed as previously described. ^[1]^ In brief, cells were washed with PBS twice and then incubated with hypotonic buffer (10 mM Tris-HCl, pH 8.0, 140 mM NaCl, 1.5 mM MgCl_2_, 0.5% NP-40, 1mM DTT, and 0.1 U/μL RNase inhibitor) on ice for 20 minutes. The supernatant was acquired as the cytoplasmic fraction following centrifugation at 1000 × g for 5 min at 4 °C. The pellets were then resuspended in nuclear resuspension buffer (20 mM HEPES, pH 7.9, 400 mM NaCl, 1 mM EGTA, 1 mM EDTA, 1 mM DTT, and 0.1 U/μL RNase inhibitors) followed by incubation at 4 °C for 30 min. The nuclear fraction was collected after removing insoluble membrane debris by centrifugation at 12000 × g for 15 min.

*RNA Extraction, Reverse Transcription, and Quantitative PCR (RT-qPCR):* Total RNA from tissues, sEVs, and cells were extracted using a TRIzol reagent (Invitrogen) according to the manufacturer’s protocol. Reverse transcription was conducted with a reverse transcription kit (AT341-02, Transgen Biotech, Beijing, China) and the RT-qPCR was performed using the Genious 2× SYBR Green Fast qPCR Mix (Abclonal) on a PikoReal 96 real-time PCR system (Thermo) following the protocol. U1 and β-actin were the control. The comparative CT methods were used to identify relative quantification. All primer sequences are listed in Supplementary Table S7.

*RACE assays:* The 5’ and 3’ Rapid-amplification of cDNA ends (RACE) assays were performed with the SMARTer RACE 5’/3’ Kit (Takara, 634858）following the manufacturer’s instructions. Gene-specific primers for touchdown PCR are listed in Supplementary Table S7.

*Vectors, Retroviral Infection, and Transfection:* For overexpression with CATED, DHX36, and RAP1A, they were cloned into the pCDH vector. For knockdown with CATED, it was cloned into pLV3 vector. The His-tagged SUMO2, Flag-tagged SUMO2, DHX36, and its truncated and mutated forms were cloned into the pcDNA3.0 vector.

The 293T cells were plated in 6 cm plates and incubated overnight. The CATED overexpression and knockdown plasmids and a corresponding control plasmid, along with their packaging vectors pMD2.G and pSPAX2, were co-transfected into the 293T cells using Lipofectamine 2000 (Invitrogen) following the manufacturer’s instructions. The resulting viral supernatant was then used to infect SKOV3 and COV504 cells. Stable cell lines in SKOV3 and COV504 were established by selecting with 2 μg/ml puromycin for two weeks.

The SKOV3 and COV504 cells were seeded in six-well plates and then grown to 50–60% confluency before being transfected with plasmids, siRNAs targeting DHX36, RAP1A, and PIAS1 (20 μM, GenePharma Technology, Shanghai, China) or ASO targeting CATED (Tsingke) using Lipofectamine 2000 (Invitrogen) for 36 h. The specific siRNAs and ASO used are listed in Supplementary Table S8.

*Cell Viability and Colony Formation:* 2,000 cells were seeded in 100 μL of complete culture media in 96-well plates and a gradient of cisplatin concentrations diluted with complete media was added after 24 h. The cell viability was detected using the Cell Counting Kit-8 assay (NCM Biotech, C6005) at 450 nm. For colony formation, cells were seeded into 6-well plates (500 cells per well) and treated with cisplatin, then fixed and stained with 0.1% crystal violet for 10 min after two weeks.

*EdU Cell Proliferation and TUNEL Assay:* The proliferation of cells was detected using EdU cell proliferation assay (Beyotime, C0071S), and the One-step TUNEL cell apoptosis assay kit (red TRITC labeled fluorescence) (KGA7061, KeyGen Biotech, China) was used to detect the apoptosis according to the instructions with modifications to adapt to our requirements. For the EdU assay, 2 × 10^5^ cells were seeded into 6-well plates and treated with cisplatin (5 μg/ml) for 36 hours. The EdU reagent (10 µM) was added to each well and incubated for 2.5 hours to label the cells. After washing three times with PBS, cells were fixed in 4% paraformaldehyde solution for 15 min, permeabilized with 0.3% Triton X-100 for an additional 15 min, and then incubated with the click-reaction reagent for 30 min at room temperature in the dark. Nuclei were counterstained with DAPI. For the TUNEL assay, the cells in 6-well plates were washed, fixed, permeabilized, and incubated with TUNEL staining reagent. We have reduced the cell exposure time (from 36 hours to 12 hours) and increased the concentration/incubation time of the permeabilization solution to increase the sensitivity of early apoptotic events and reduce the background signal. Both the staining results were observed using a confocal microscope (Zeiss LSM980 with Airyscan), and the number of positive cells was counted and calculated using ImageJ.

*Flow Cytometric Cell Death Assay:* We plated cells in each well of a six-well plate and cultured them overnight until they reached 70–80% confluency. The cells were treated with cisplatin for 36 h, then collected and washed twice in binding buffer. After discarding the supernatant, we added 500 µL of binding buffer, 5 µL of Annexin V-FITC, and 5 µL of propyl iodide (PI) solution (KeyGEN BioTECH), mixing gently. The mixtures were incubated in the dark at room temperature for 20 min before analysis by flow cytometry. The percentage of cells in normal, early apoptotic, and late apoptotic phases was evaluated using CytExpert software (Version 2.3, Beckman, United States).

*Immunohistochemistry (IHC):* The formalin-fixed and paraffin-embedded tissues from patients with HGSOC and nude mice were analyzed by IHC. The tissue sections were initially incubated at 60°C for 2 h, dewaxed with dimethylbenzene, and then rehydrated through a series of alcohol baths (100%, 90%, 80%, 70%). After antigen retrieval was performed, endogenous peroxidase activity was quenched with 3% hydrogen peroxide. Subsequently, the sections were blocked with 5% BSA for 30 min and then incubated with primary antibodies overnight at 4 °C. At last, the sections were treated with secondary antibodies, and DAB was applied for antigen visualization, with hematoxylin used for nuclear counterstaining. Images were acquired by an upright microscope (Olympus, Tokyo, Japan) and analyzed by ImageJ. All antibodies are listed in Supplementary Table S6.

*GO Analysis:* The GO analysis was conducted by Metascape web-server with default parameters and the data was visualized by the ggplot2 package in R software.

*Coding Potential Calculator (CPC):* To assess the protein-coding potential of CATED, the coding potential calculator tool was used to follow the guideline (https://cpc.gao-lab.org/). ^[2]^

*Kaplan-Meier Plotter Database:* To analyze the association between the progress-free survival of ovarian cancer patients and CATED levels, the Kaplan-Meier Plotter Database was used (https://www.kmplot.com/analysis/).

*Dual-Luciferase Reporter Assay:* The 5’ UTR sequence of RAP1A mRNA was used as the 5’ UTR of firefly luciferase in the pmirGLO. The renilla luciferase was employed as a loading control. Following a 24 h transfection in SKOV3 and COV504 cells, relative luciferase activities were assessed using the Dual-luciferase Reporter Assay System (Beyotime, RG028) according to the manufacturer’s guidelines.

**References for Supporting Information**

1. H. Liu, S. Deng, X. Yao, Y. Liu, L. Qian, Y. Wang, T. Zhang, G. Shan, L. Chen, Y. Zhou. Ascites exosomal lncRNA PLADE enhances platinum sensitivity by inducing R-loops in ovarian cancer. *Oncogene* 2024, 43(10):714-728.

2. L. Kong, Y. Zhang, Z. Q. Ye, X. Q. Liu, S. Q. Zhao, L. Wei, G. Gao. CPC: assess the protein-coding potential of transcripts using sequence features and support vector machine. *Nucleic Acids Res* 2007;35(Web Server issue):W345-9.
