# Supplementary material for: Tumor Small Extracellular Vesicle‐Transmitted LncRNA CATED Promotes Platinum‐Resistance in High‐Grade Serous Ovarian Cancer
Source: Adv Sci (Weinh). 2025 Jun 10;12(31):e05963. doi: 10.1002/advs.202505963 (PMC12376677; doi:10.1002/advs.202505963)
Supplement: Supplementary file 3 — Supporting Information [file ADVS-12-e05963-s003.pdf]

ADVANCED SCIENCE
Open Access

Supporting Information

for *Adv. Sci.*, DOI 10.1002/advs.202505963

Tumor Small Extracellular Vesicle-Transmitted LncRNA CATED Promotes Platinum-Resistance in High-Grade Serous Ovarian Cancer

Yi Liu, Hanyuan Liu, Chenchen Zhu, Yan Yang, Zhen Shen, Ge Shan, Liang Chen* and Ying Zhou**

Figure S1

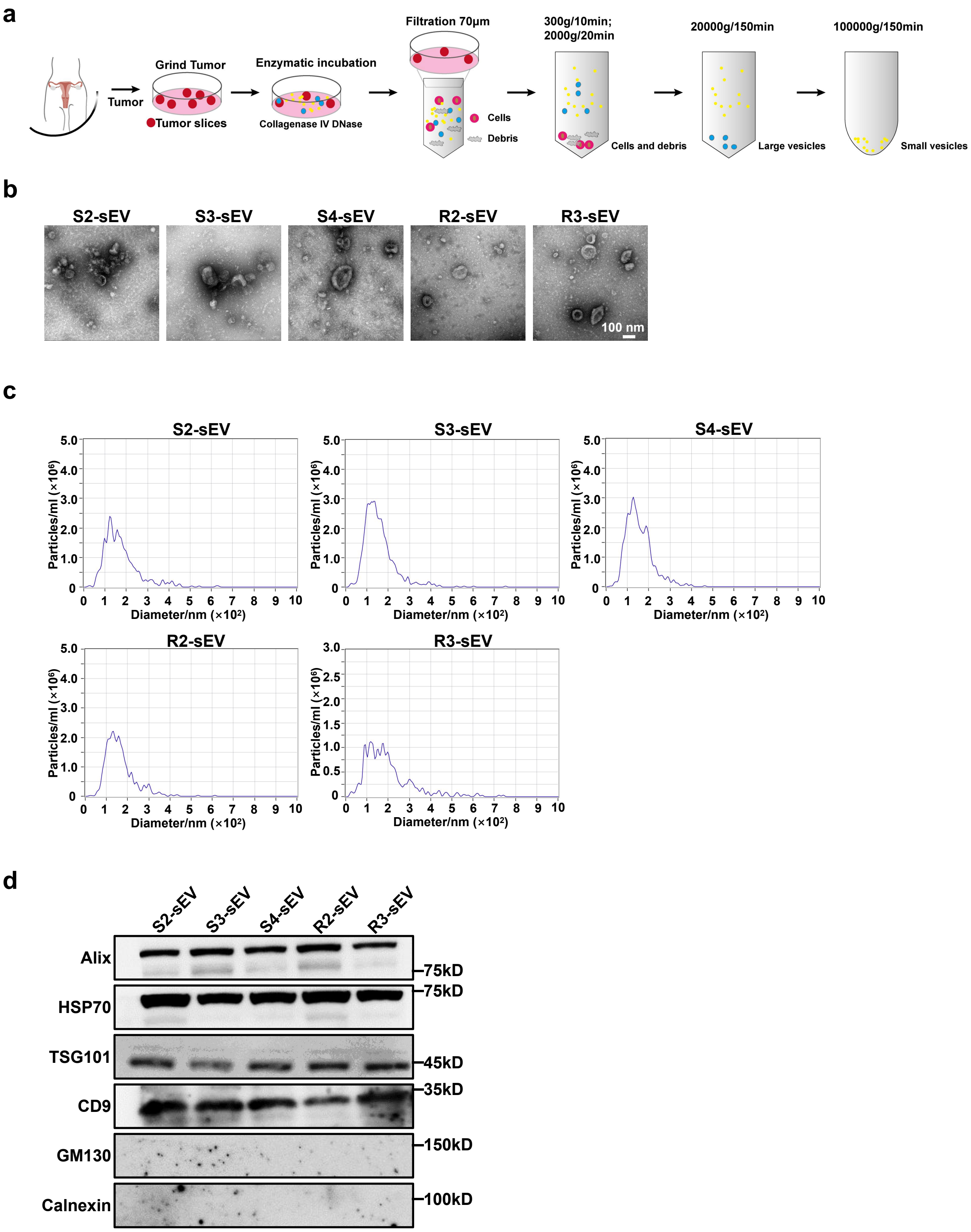
a
Tumor
Grind Tumor
Tumor slices
Enzymatic incubation
Collagenase IV DNase
Filtration 70μm
Cells
Debris
300g/10min;
2000g/20min
Cells and debris
20000g/150min
Large vesicles
100000g/150min
Small vesicles
b
S2-sEV
S3-sEV
S4-sEV
R2-sEV
R3-sEV
100 nm
c
S2-sEV
S3-sEV
S4-sEV
R2-sEV
R3-sEV
Particles/ml (×10⁶)
Diameter/nm (×10²)
d
S2-sEV
S3-sEV
S4-sEV
R2-sEV
R3-sEV
Alix
HSP70
TSG101
CD9
GM130
Calnexin
75kD
75kD
45kD
35kD
150kD
100kD

Figure S2

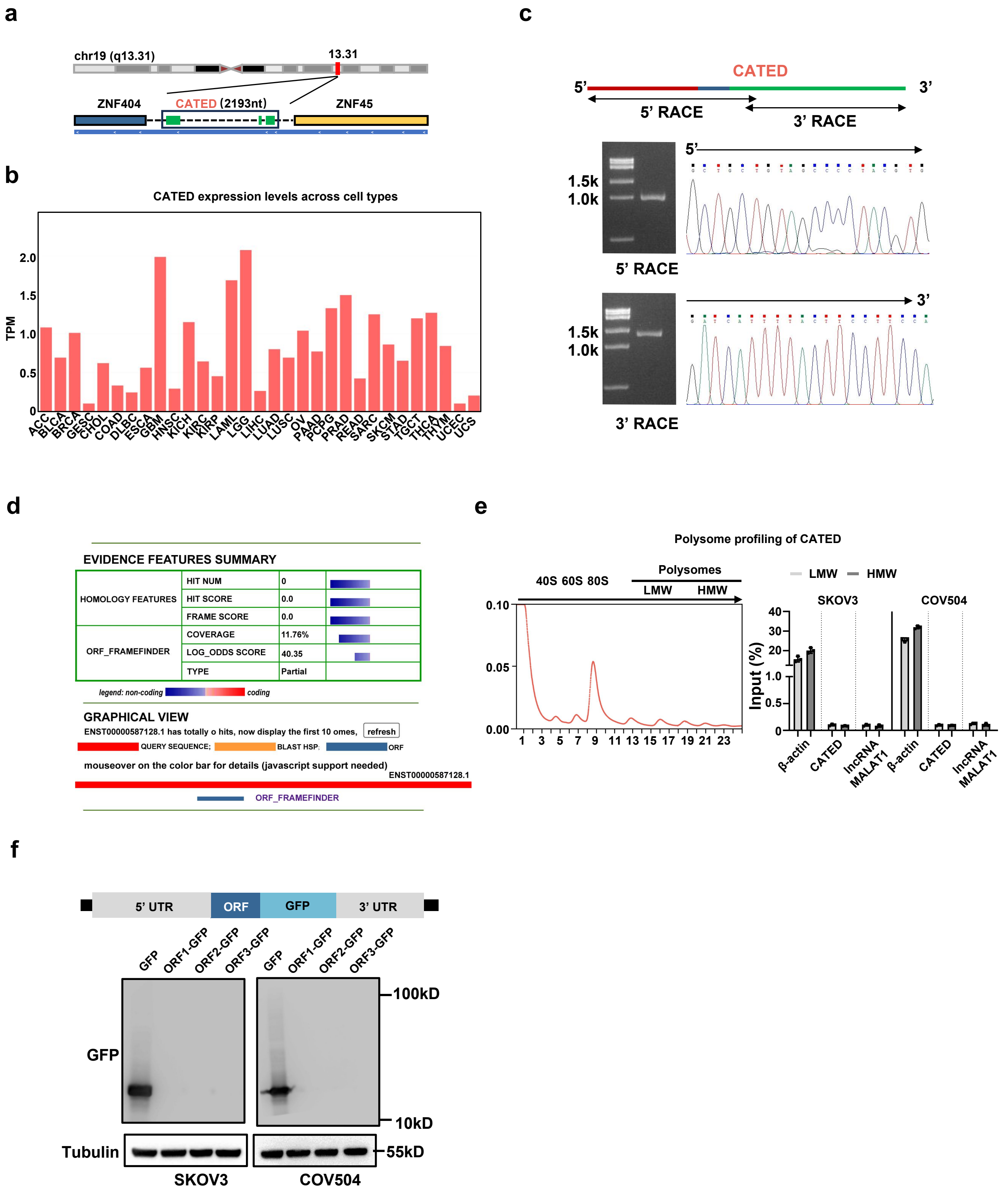

a
chr19 (q13.31)
13.31
ZNF404
CATED (2193nt)
ZNF45
b
CATED expression levels across cell types
TPM
2.0
1.5
1.0
0.5
0
ACC
BLCA
BRCA
CESC
CHOL
COAD
DLBC
ESCA
GBM
HNSC
KICH
KIRC
KIRP
LAML
LGG
LIHC
LUAD
LUSC
OV
PAAD
PCPG
PRAD
READ
SARC
SKCM
STAD
TGCT
THCA
THYM
UCEC
UCS
c
CATED
5’
3’
5’ RACE
3’ RACE
1.5k
1.0k
5’ RACE
3’ RACE
d
EVIDENCE FEATURES SUMMARY
HOMOLOGY FEATURES
HIT NUM 0
HIT SCORE 0.0
FRAME SCORE 0.0
ORF_FRAMEFINDER
COVERAGE 11.76%
LOG_ODDS SCORE 40.35
TYPE Partial
legend: non-coding coding
GRAPHICAL VIEW
ENST00000587128.1 has totally o hits, now display the first 10 omes,
refresh
QUERY SEQUENCE;
BLAST HSP:
ORF
mouseover on the color bar for details (javascript support needed)
ENST00000587128.1
ORF_FRAMEFINDER
e
Polysome profiling of CATED
40S 60S 80S
Polysomes
LMW
HMW
0.10
0.05
0.00
1 3 5 7 9 11 13 15 17 19 21 23
LMW
HMW
SKOV3
COV504
Input (%)
40
30
20
1.0
0.5
0
β-actin
CATED
lncRNA MALAT1
f
5’ UTR
ORF
GFP
3’ UTR
GFP
ORF1-GFP
ORF2-GFP
ORF3-GFP
100kD
10kD
55kD
Tubulin
SKOV3
COV504

Figure S3

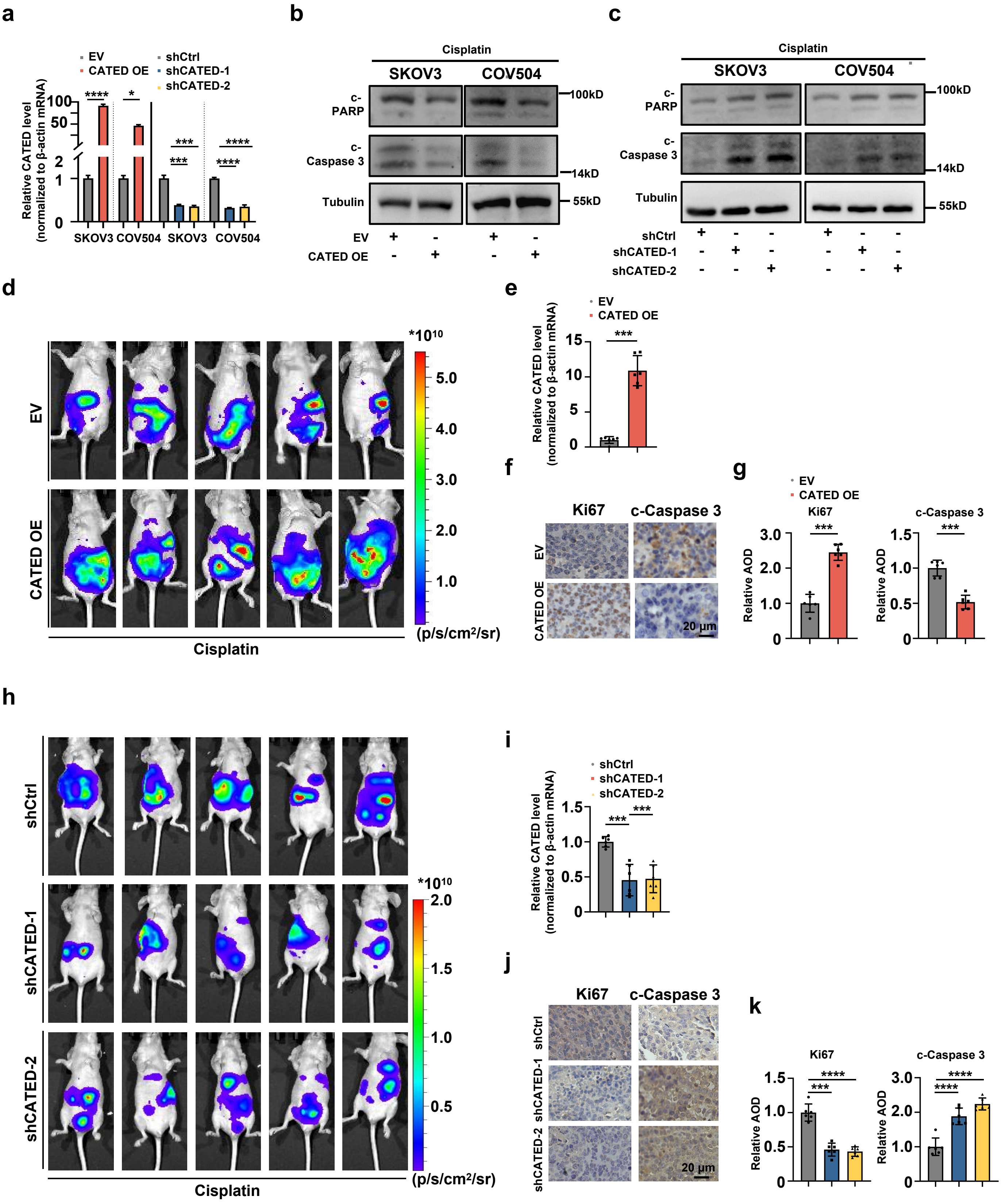

a
EV
CATED OE
shCtrl
shCATED-1
shCATED-2
Relative CATED level (normalized to β-actin mRNA)
SKOV3 COV504 SKOV3 COV504
b
Cisplatin
SKOV3 COV504
c-PARP
c-Caspase 3
Tubulin
100kD
14kD
55kD
EV
CATED OE
c
Cisplatin
SKOV3 COV504
c-PARP
c-Caspase 3
Tubulin
100kD
14kD
55kD
shCtrl
shCATED-1
shCATED-2
d
EV
CATED OE
*10^10
(p/s/cm²/sr)
Cisplatin
e
EV
CATED OE
Relative CATED level (normalized to β-actin mRNA)
f
Ki67
c-Caspase 3
EV
CATED OE
20 μm
g
EV
CATED OE
Ki67
c-Caspase 3
Relative AOD
h
shCtrl
shCATED-1
shCATED-2
*10^10
(p/s/cm²/sr)
Cisplatin
i
shCtrl
shCATED-1
shCATED-2
Relative CATED level (normalized to β-actin mRNA)
j
Ki67
c-Caspase 3
shCtrl
shCATED-1
shCATED-2
20 μm
k
Ki67
c-Caspase 3
Relative AOD

Figure S4

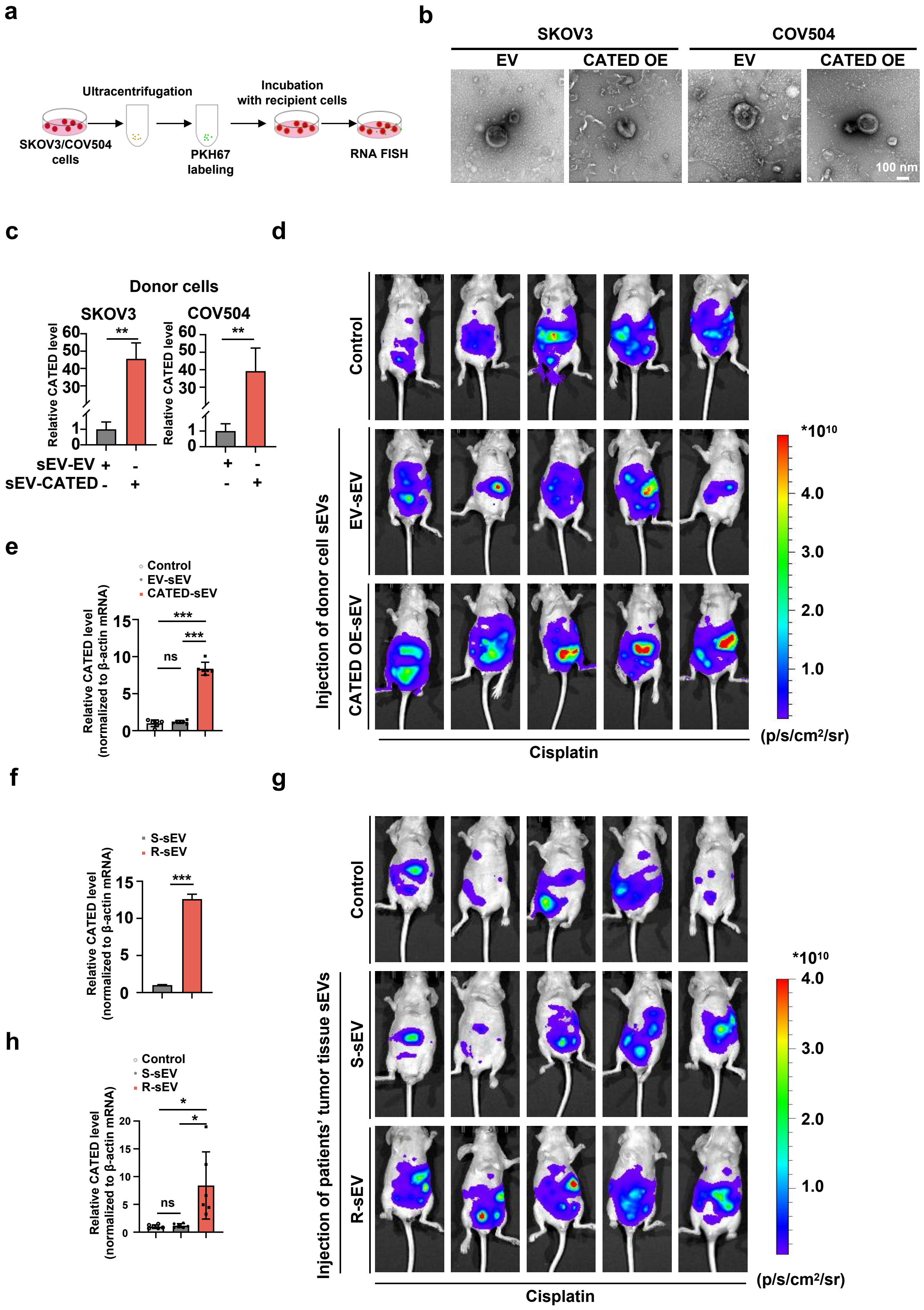

a
Ultracentrifugation
Incubation
with recipient cells
SKOV3/COV504
cells
PKH67
labeling
RNA FISH
b
SKOV3
COV504
EV
CATED OE
EV
CATED OE
100 nm
c
Donor cells
SKOV3
COV504
Relative CATED level
sEV-EV
sEV-CATED
d
Control
EV-sEV
CATED OE-sEV
Injection of donor cell sEVs
Cisplatin
*10^10
(p/s/cm2/sr)
e
Control
EV-sEV
CATED-sEV
Relative CATED level
(normalized to β-actin mRNA)
ns
f
S-sEV
R-sEV
Relative CATED level
(normalized to β-actin mRNA)
g
Control
S-sEV
R-sEV
Injection of patients' tumor tissue sEVs
Cisplatin
*10^10
(p/s/cm2/sr)
h
Control
S-sEV
R-sEV
Relative CATED level
(normalized to β-actin mRNA)
ns

Figure S5

a

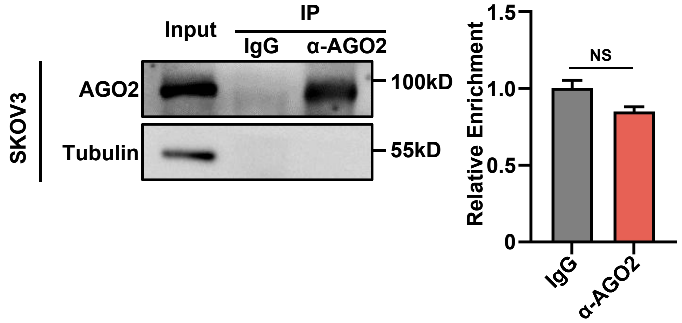

c

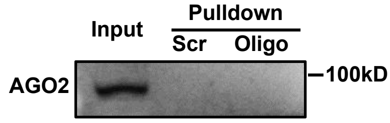

b

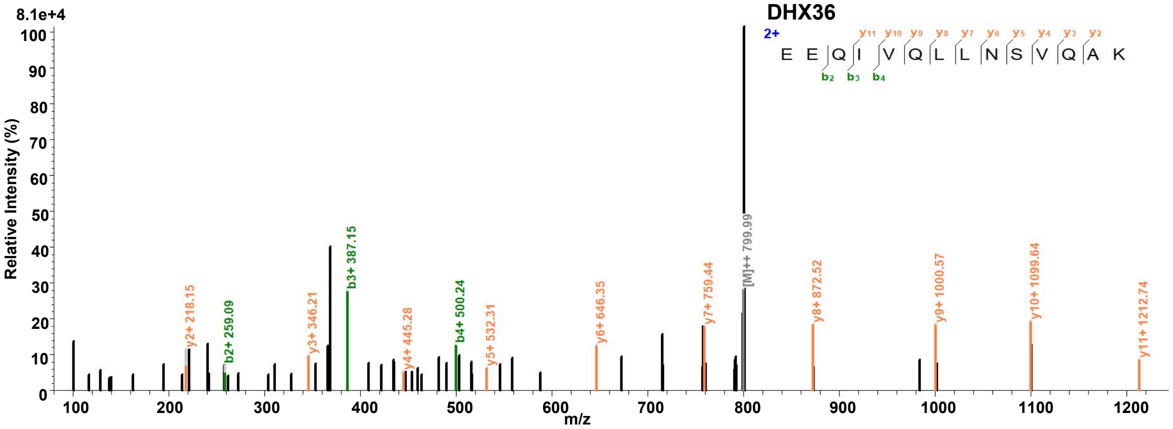

d

Rank	Motif	P-value	Log P-pvalue	% of Targets	% of Background
1	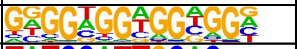	1e-4693	-1.081e+04	18.02%	4.08%
2	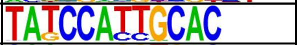	1e-4363	-1.005e+04	2.04%	0.00%
3	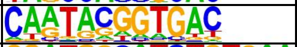	1e-1470	-3.386e+03	0.81%	0.00%
4	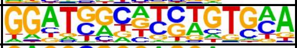	1e-1403	-3.232e+03	0.77%	0.00%
5	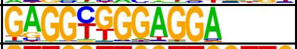	1e-1275	-2.936e+03	2.91%	0.33%
6	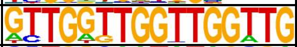	1e-1255	-2.891e+03	0.71%	0.00%
7	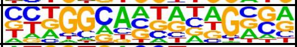	1e-1149	-2.647e+03	0.74%	0.00%
8	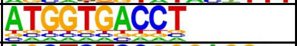	1e-1108	-2.551e+03	0.71%	0.00%
9	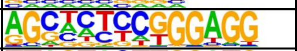	1e-1014	-2.337e+03	1.33%	0.05%
10	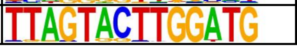	1e-1011	-2.329e+03	0.66%	0.00%

e

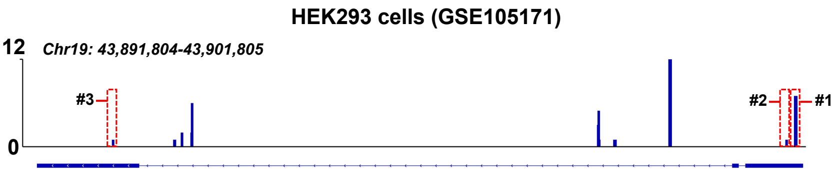

CATED

#1: ATGAGAGGCGCTGAAGACCAGAAGACCCAGGGCGT

Motif 8 Motif 6

Motif 3

#1^{mut}: ATGTCTCTTAGGTTGGACCAGAACTGTGTCTTGAC

#2: ACTGTGTGTGTGTGTGTG

Motif 8

#2^{mut}: ACCTACATGCGCCTGCGC

#3: TAATTTCCTGGTTTTGATAATTATC

Motif 7

#3^{mut}: TAATTTGGACCGGCCTTTAATTATC

f

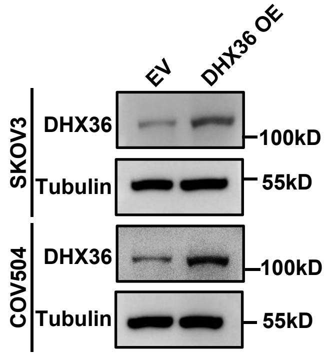

Figure S6

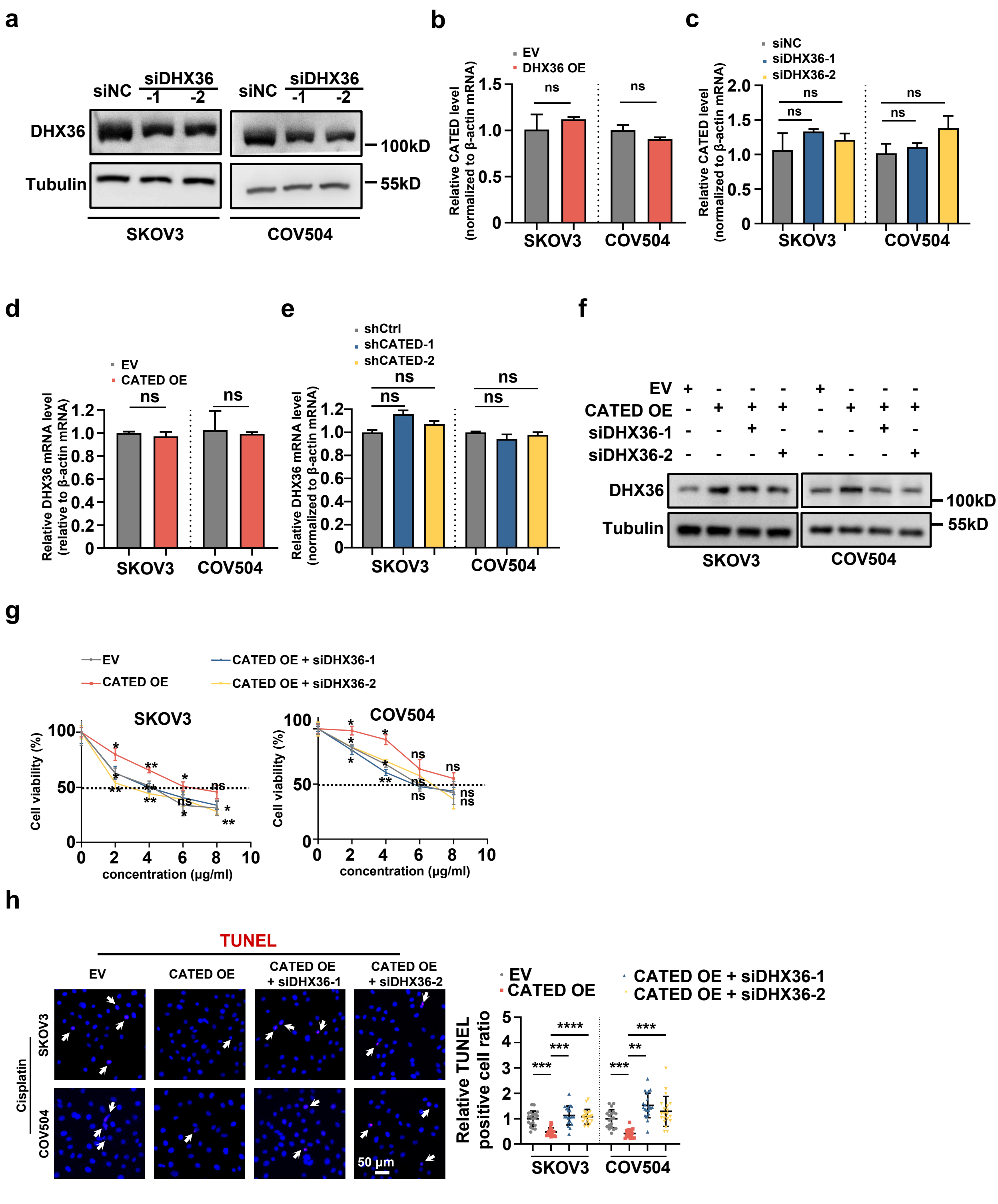

a
siNC
siDHX36 -1 -2
DHX36
Tubulin
100kD
55kD
SKOV3
COV504
b
EV
DHX36 OE
Relative CATED level (normalized to β-actin mRNA)
ns
SKOV3
COV504
c
siNC
siDHX36-1
siDHX36-2
Relative CATED level (normalized to β-actin mRNA)
d
EV
CATED OE
Relative DHX36 mRNA level (relative to β-actin mRNA)
e
shCtrl
shCATED-1
shCATED-2
Relative DHX36 mRNA level (normalized to β-actin mRNA)
f
EV
CATED OE
siDHX36-1
siDHX36-2
g
EV
CATED OE
CATED OE + siDHX36-1
CATED OE + siDHX36-2
Cell viability (%)
concentration (μg/ml)
h
TUNEL
CATED OE + siDHX36-1
CATED OE + siDHX36-2
Cisplatin
50 μm
Relative TUNEL positive cell ratio

Figure S7

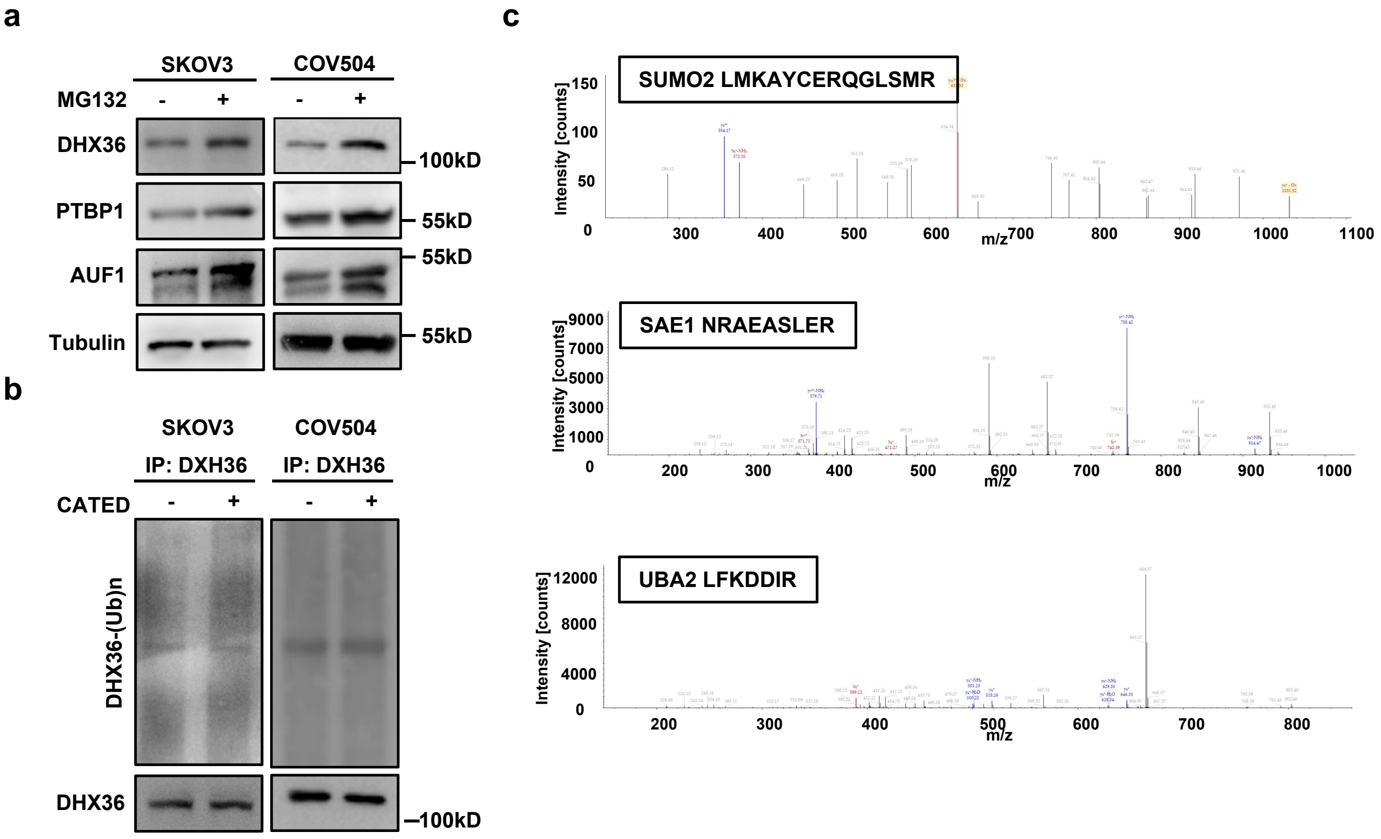

d

SUMOylaiton Sites Prediction on DHX36

No.	Position	Peptide
1	105	LLNSVQAKNDKESEA
2	139	NTPCSENKLDIQEKK
3	68	EIGMWYAKKQGQKNK
4	69	IGMWYAKKQGQKNKE
5	68	EIGMWYAKKQGQKNK
6	69	IGMWYAKKQGQKNKE
7	146	KLDIQEKKLINQEKK
8	183	PDGTLDQKLLEDLQK
9	419	KEHRSQFKRGFMQGH
10	432	GHVNRQEKEEKEAIY

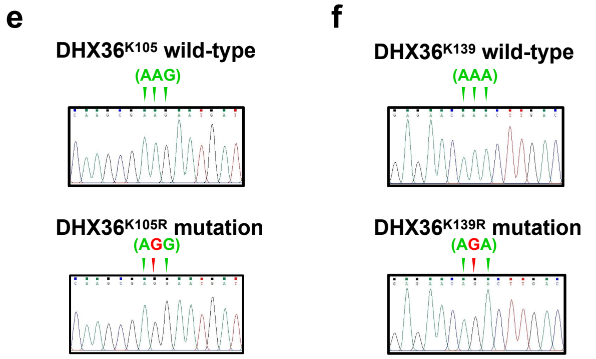

g

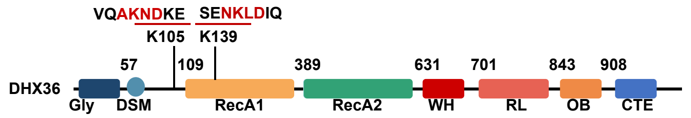

Figure S8

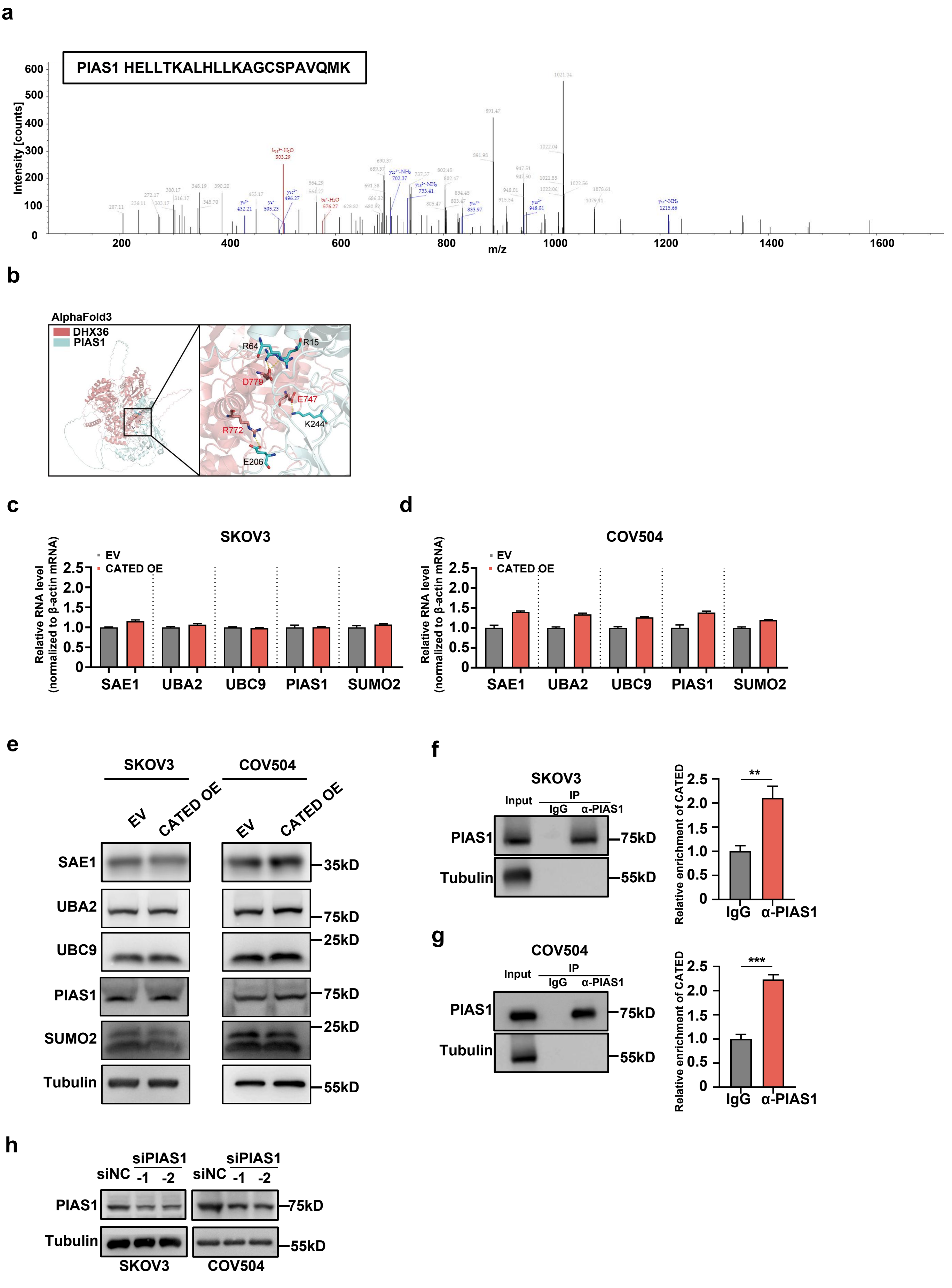

a
PIAS1 HELLTKALHLLKAGCSPAVQMK
Intensity [counts]
m/z
b
AlphaFold3
DHX36
PIAS1
R64
R15
D779
E747
K244
R772
E206
c
SKOV3
EV
CATED OE
Relative RNA level (normalized to β-actin mRNA)
SAE1
UBA2
UBC9
PIAS1
SUMO2
d
COV504
EV
CATED OE
Relative RNA level (normalized to β-actin mRNA)
SAE1
UBA2
UBC9
PIAS1
SUMO2
e
SKOV3
COV504
EV
CATED OE
SAE1
UBA2
UBC9
PIAS1
SUMO2
Tubulin
35kD
75kD
25kD
75kD
25kD
55kD
f
SKOV3
Input
IP
IgG
α-PIAS1
PIAS1
Tubulin
75kD
55kD
Relative enrichment of CATED
**
IgG
α-PIAS1
g
COV504
Input
IP
IgG
α-PIAS1
PIAS1
Tubulin
75kD
55kD
Relative enrichment of CATED
***
IgG
α-PIAS1
h
siNC
siPIAS1
-1
-2
PIAS1
Tubulin
75kD
55kD
SKOV3
COV504

Figure S9

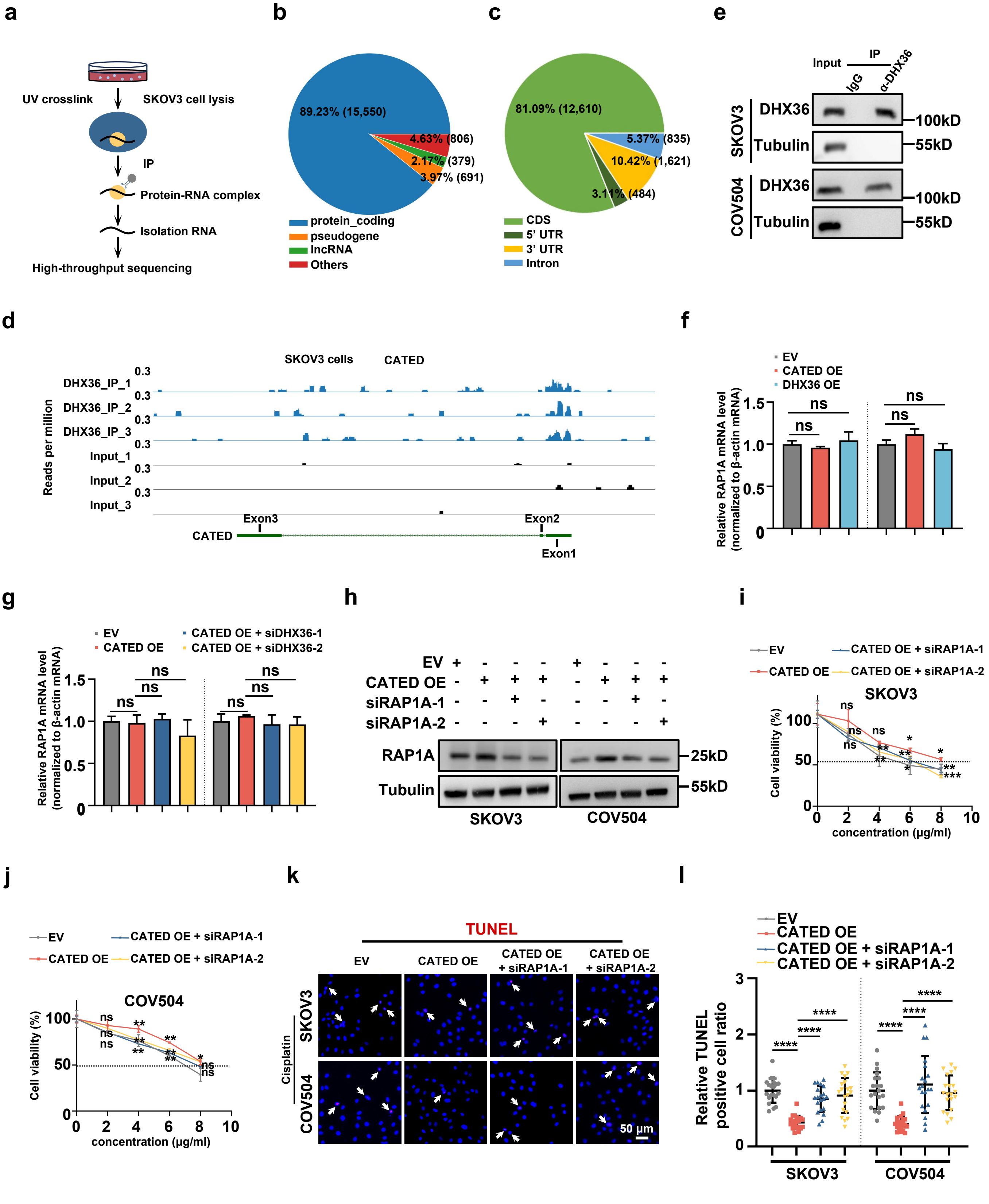

a
UV crosslink
SKOV3 cell lysis
IP
Protein-RNA complex
Isolation RNA
High-throughput sequencing
b
89.23% (15,550)
4.63% (806)
2.17% (379)
3.97% (691)
protein_coding
pseudogene
lncRNA
Others
c
81.09% (12,610)
5.37% (835)
10.42% (1,621)
3.11% (484)
CDS
5' UTR
3' UTR
Intron
e
Input
IP
IgG
α-DHX36
SKOV3
COV504
DHX36
Tubulin
100kD
55kD
d
SKOV3 cells
CATED
Reads per million
DHX36_IP_1
DHX36_IP_2
DHX36_IP_3
Input_1
Input_2
Input_3
0.3
Exon3
Exon2
Exon1
f
EV
CATED OE
DHX36 OE
Relative RAP1A mRNA level (normalized to β-actin mRNA)
ns
g
EV
CATED OE
CATED OE + siDHX36-1
CATED OE + siDHX36-2
Relative RAP1A mRNA level (normalized to β-actin mRNA)
ns
h
EV
CATED OE
siRAP1A-1
siRAP1A-2
RAP1A
Tubulin
25kD
55kD
SKOV3
COV504
i
EV
CATED OE
CATED OE + siRAP1A-1
CATED OE + siRAP1A-2
SKOV3
Cell viability (%)
concentration (μg/ml)
j
EV
CATED OE
CATED OE + siRAP1A-1
CATED OE + siRAP1A-2
COV504
Cell viability (%)
concentration (μg/ml)
k
TUNEL
EV
CATED OE
CATED OE + siRAP1A-1
CATED OE + siRAP1A-2
Cisplatin
SKOV3
COV504
50 μm
l
EV
CATED OE
CATED OE + siRAP1A-1
CATED OE + siRAP1A-2
Relative TUNEL positive cell ratio
****
SKOV3
COV504

Figure S10

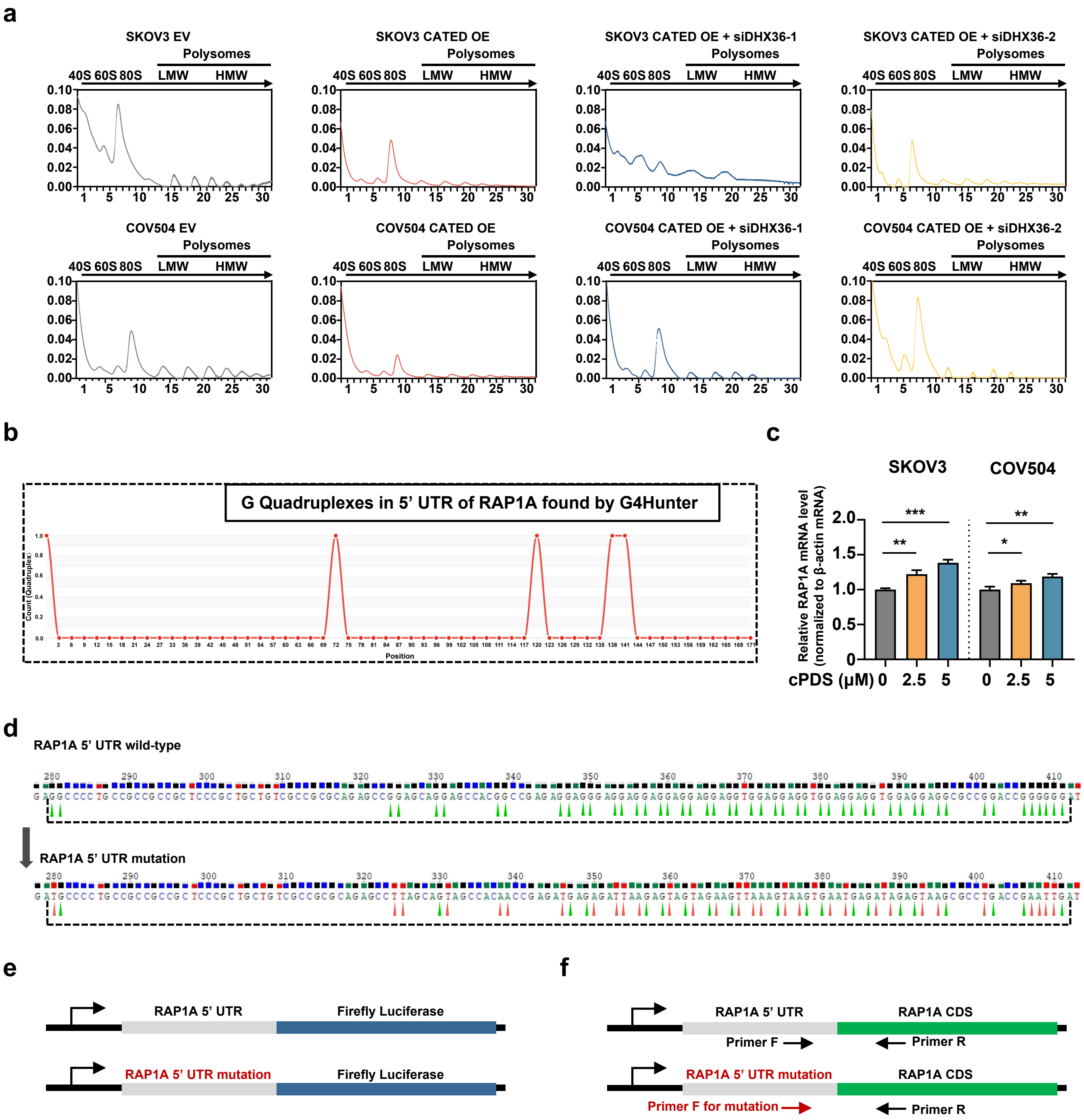
a
SKOV3 EV
SKOV3 CATED OE
SKOV3 CATED OE + siDHX36-1
SKOV3 CATED OE + siDHX36-2
COV504 EV
COV504 CATED OE
COV504 CATED OE + siDHX36-1
COV504 CATED OE + siDHX36-2
Polysomes
40S 60S 80S
LMW
HMW
b
G Quadruplexes in 5' UTR of RAP1A found by G4Hunter
Count (Quadruplex)
Position
c
SKOV3
COV504
Relative RAP1A mRNA level (normalized to β-actin mRNA)
cPDS (μM)
d
RAP1A 5' UTR wild-type
RAP1A 5' UTR mutation
e
RAP1A 5' UTR
Firefly Luciferase
RAP1A 5' UTR mutation
Firefly Luciferase
f
RAP1A 5' UTR
RAP1A CDS
Primer F
Primer R
RAP1A 5' UTR mutation
RAP1A CDS
Primer F for mutation
Primer R

Figure S11

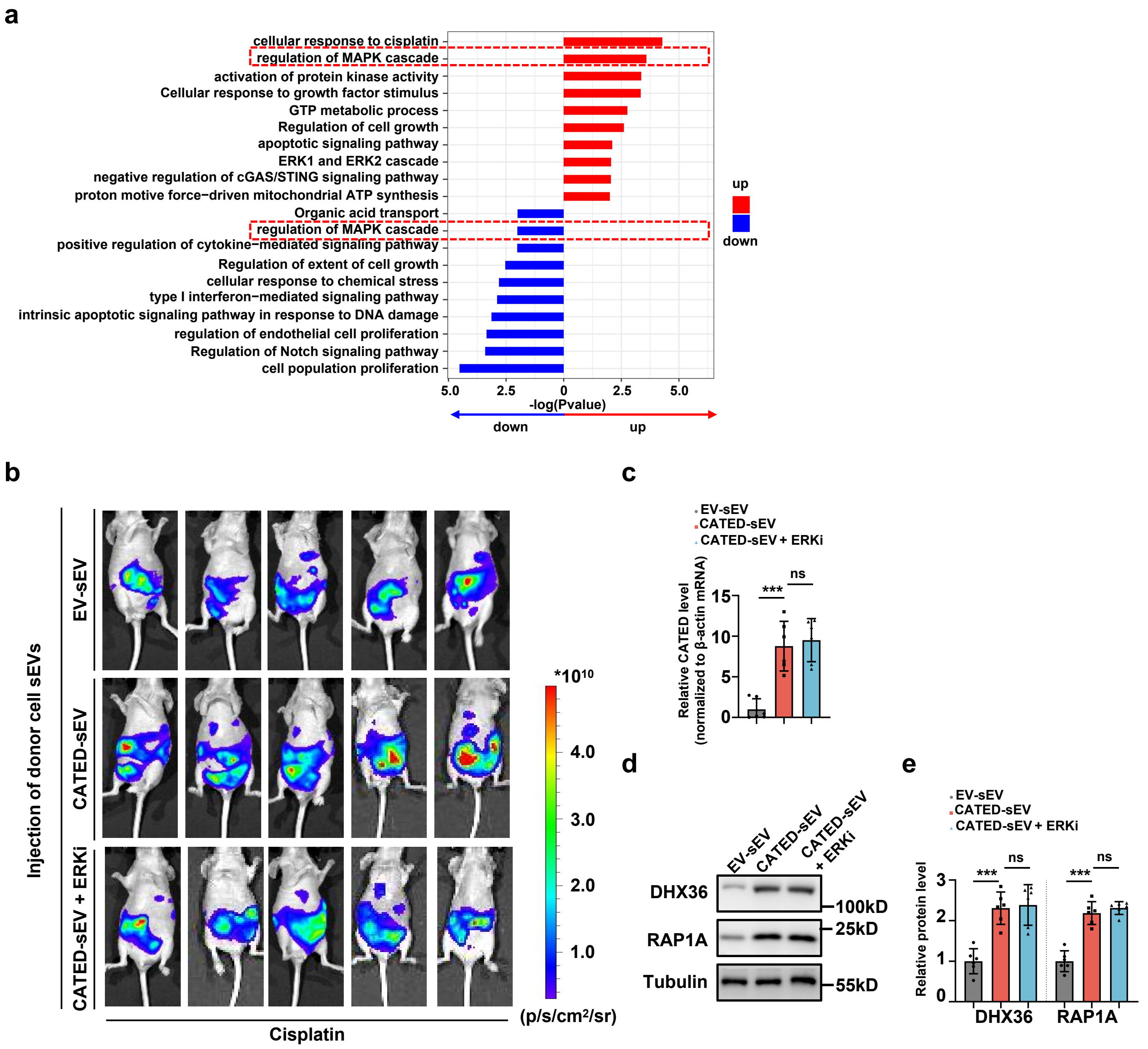
a
cellular response to cisplatin
regulation of MAPK cascade
activation of protein kinase activity
Cellular response to growth factor stimulus
GTP metabolic process
Regulation of cell growth
apoptotic signaling pathway
ERK1 and ERK2 cascade
negative regulation of cGAS/STING signaling pathway
proton motive force−driven mitochondrial ATP synthesis
Organic acid transport
regulation of MAPK cascade
positive regulation of cytokine−mediated signaling pathway
Regulation of extent of cell growth
cellular response to chemical stress
type I interferon−mediated signaling pathway
intrinsic apoptotic signaling pathway in response to DNA damage
regulation of endothelial cell proliferation
Regulation of Notch signaling pathway
cell population proliferation
5.0 2.5 0 2.5 5.0
-log(Pvalue)
down
up
b
Injection of donor cell sEVs
EV-sEV
CATED-sEV
CATED-sEV + ERKi
Cisplatin
*10^10
4.0
3.0
2.0
1.0
(p/s/cm²/sr)
c
EV-sEV
CATED-sEV
CATED-sEV + ERKi
Relative CATED level (normalized to β-actin mRNA)
***
ns
d
EV-sEV
CATED-sEV
CATED-sEV + ERKi
DHX36
RAP1A
Tubulin
100kD
25kD
55kD
e
EV-sEV
CATED-sEV
CATED-sEV + ERKi
Relative protein level
DHX36
RAP1A

Figure S12

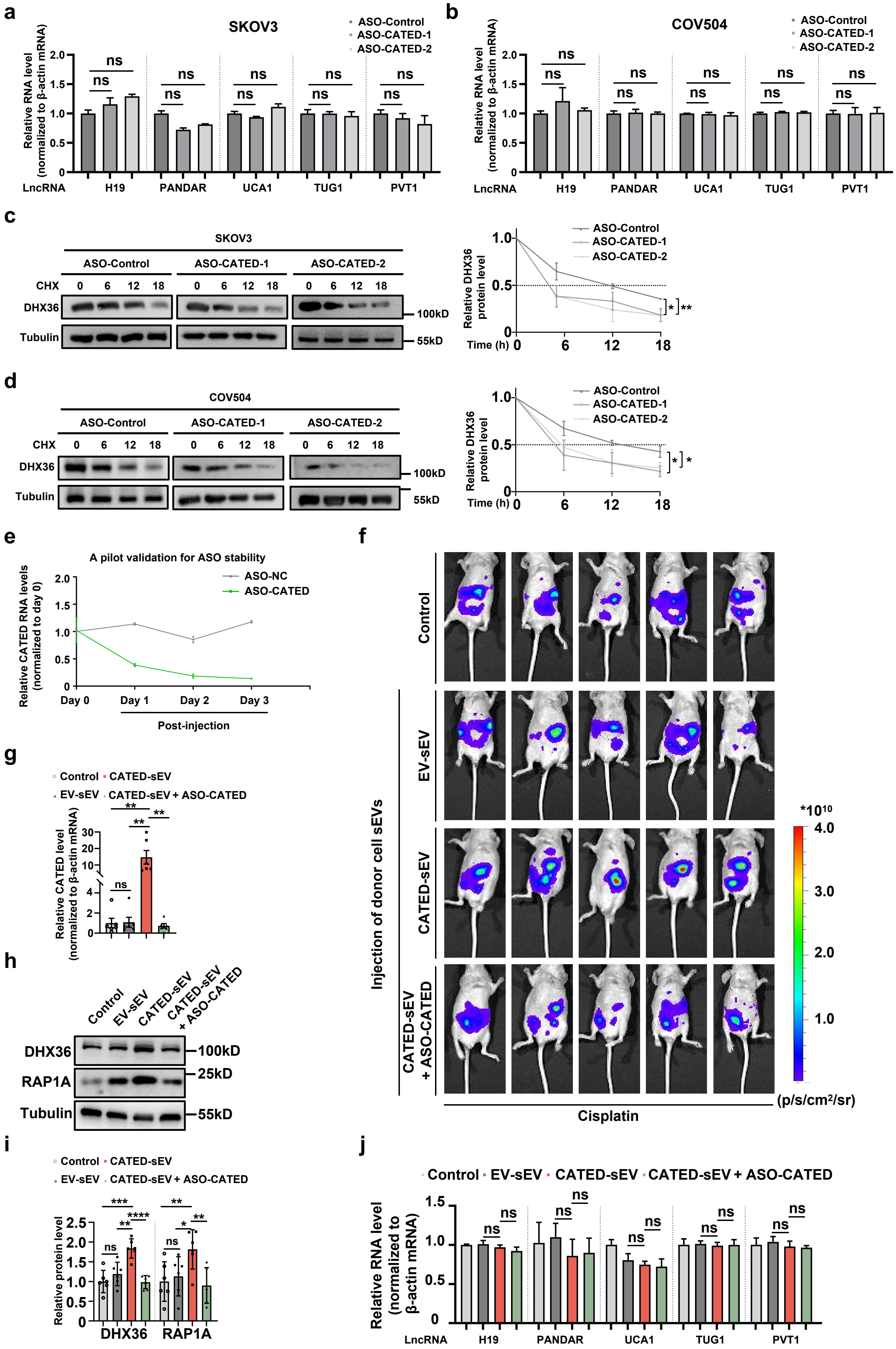

a
SKOV3
ASO-Control
ASO-CATED-1
ASO-CATED-2
Relative RNA level (normalized to β-actin mRNA)
LncRNA
H19
PANDAR
UCA1
TUG1
PVT1
b
COV504
c
SKOV3
CHX
DHX36
Tubulin
100kD
55kD
Relative DHX36 protein level
Time (h)
d
COV504
e
A pilot validation for ASO stability
ASO-NC
ASO-CATED
Relative CATED RNA levels (normalized to day 0)
Day 0
Day 1
Day 2
Day 3
Post-injection
f
Control
EV-sEV
CATED-sEV
CATED-sEV + ASO-CATED
Injection of donor cell sEVs
Cisplatin
(p/s/cm²/sr)
g
Relative CATED level (normalized to β-actin mRNA)
h
RAP1A
25kD
i
Relative protein level
j
